# Dynamically Interacting Protein Networks Provide a Mechanism to Overcome the Enormous Intrinsic Barrier to Orotidine 5′-Monophosphate Decarboxylation

**DOI:** 10.1021/acscentsci.5c00590

**Published:** 2025-07-11

**Authors:** Pankaj Dubey, Anish Somani, Jessica Lin, Anthony T. Iavarone, Judith P. Klinman

**Affiliations:** † California Institute for Quantitative Biosciences, 1438 University of California Berkeley, Berkeley, California 94720, United States; ‡ Department of Chemistry, University of California Berkeley, Berkeley, California 94720, United States; § Department of Bioengineering, University of California Berkeley, Berkeley, California 94720, United States; ∥ Department of Molecular and Cell Biology, University of California Berkeley, Berkeley, California 94720, United States

## Abstract

Orotidine 5′-monophosphate decarboxylase (OMPDC) is among the most efficient enzymes known, accelerating the decarboxylation of the OMP by ∼17 orders of magnitude, primarily by lowering the enthalpy of activation by ∼28 kcal/mol. Despite this feature, OMPDC from *Methanothermobacter thermautotrophicus* requires ∼15 kcal/mol of activation energy following ES complex formation. This study applies temperature-dependent hydrogen–deuterium exchange mass spectrometry (TDHDX) to detect site-specific thermal protein networks that channel energy from solvent collisions to the active site. Comparative TDHDX of native OMPDC and a single-site variant (Leu123Ala) that alters the activation enthalpy for catalytic turnover reveals region-specific changes in protein flexibility, connecting local scaffold unfolding enthalpy to the activation barrier of catalysis. The data implicate four spatially resolved, thermally sensitive networks that originate at distinct protein–solvent interfaces and converge near the substrate phosphate-binding region (R203), the ribose-binding region (K42), and a catalytic loop (S127). These networks are proposed to act synergistically to optimize substrate positioning and active site electrostatics for the activated complex formation. The complexity of the identified thermal activation pathways distinguishes Mt-OMPDC from other TIM barrel enzymes previously studied by TDHDX. The findings highlight the essential role of scaffold dynamics in enzyme function with broad implications for designing efficient biocatalysts.

## Graphical abstract

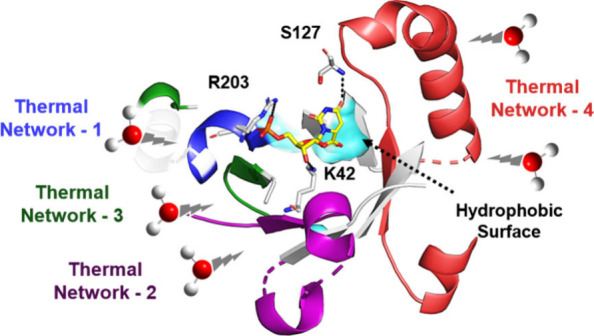

## Introduction

Orotidine 5′-monophosphate decarboxylase (OMPDC) is an extraordinarily efficient enzyme that accelerates the unimolecular decarboxylation of orotidine 5′-monophosphate (OMP) to uridine 5′-monophosphate (UMP) by an average of 17 orders of magnitude (*k*_cat_/*k*_non_,), corresponding to a reduction in the free energy barrier for decarboxylation of ca. 22 kcal/mol.^1,2^ This substantial reduction is largely due to a decrease in the observed activation enthalpy (Δ*H* ^‡^), with minimal contribution from entropy changes (*T*Δ*S* ^‡^), cf. Figure 1a.^3–5^ The majority of other highly efficient enzymes^6,7^ also achieve their catalytic power by significantly lowering their activation enthalpies, underscoring the crucial role of reduced enthalpic barriers to effect high catalytic rate enhancements. OMPDC functions exclusively in its dimeric form, with all known crystal structures exhibiting a homodimeric TIM barrel fold.^8,9^ A comparison of crystal structures for the apo-form of *Methanothermobacter thermautotrophicus* (Mt-OMPDC) to the enzyme bound with 6-azaUMP, a tight-binding competitive inhibitor described as a transition state analog (TSA),^10,11^ reveals a regional conformational change (RMSD of 0.5 Å) that includes a loop7 closure and shielding of the active site from solvent exposure, Figure 1b. The active site of Mt-OMPDC in the presence of 6-azaUMP further indicates the importance of a tetrad of charged residues, a phosphate-binding residue (R203), and a cluster of hydrophobic residues within the barrel cavity (Figure 1c).

**Figure 1:**
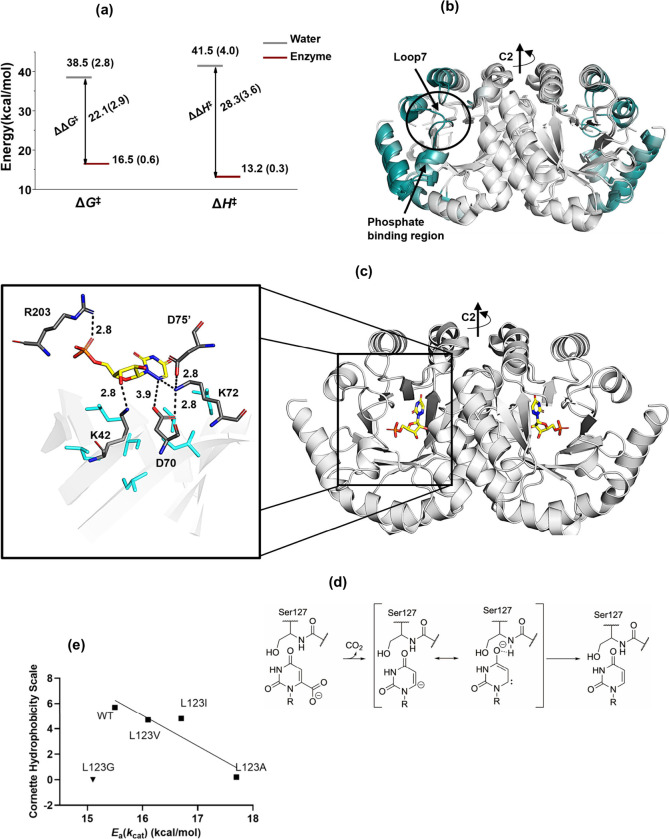
**Structural and energetic insights into OMP decarboxylation catalyzed by OMP decarboxylases.** (a) Free energy (Δ*G* ^‡^) and activation enthalpy (Δ*H* ^‡^) for the reaction for the OMP decarboxylation, showing the energy differences between the reaction in water and in the presence of enzyme. Data for the reaction in water were derived from OMP analogue decarboxylation in aqueous solution,^4^ followed by averaging, while enzyme data were based on the averaged reaction energetics of *Escherichia coli* OMP decarboxylase (Ec-OMPDC), *Saccharomyces cerevisiae* OMP decarboxylase (Sc-OMPDC), and *Methanothermobacter thermautotrophicus* OMP decarboxylase (Mt-OMPDC) under *k*_cat_ conditions.^5^ (b) Crystal structure overlay of Mt-OMPDC in its open (apo) form (PDB: 3G18) and ligand-bound (6-azaUMP) closed form (PDB: 3G1A). Residue-specific RMSD values were calculated to identify structural changes upon ligand binding and visualized on the crystal structure using the PyMOL script ColorbyRMSD,^51^ with a white-gray-deep teal color gradient. Regions showing minimal structural differences are depicted in white, while regions with increasing RMSD values are colored deep teal. Loop7 (residues 180–188), highlighted by a circle, is unstructured in the apo form and adopts a well-ordered closed conformation over the ligand in the bound form. Due to its unstructured nature in the apo form, RMSD values for this loop could not be calculated, and it was manually colored deep teal for visual clarity. (c) Crystal structure of the Mt-OMPDC homodimer bound to the tight binding analogue 6-azaUMP (PDB: 3G1A), where 6-azaUMP is shown in yellow stick representation. The active site of Mt-OMPDC is highlighted, featuring the catalytic tetrad (K42, D70, K72, and D75′) along with R203, shown in dark gray with labeled interaction distances between the substrate and these residues. Hydrophobic residues from the β-strands (I16, I68, I96, L123, V155, I178, I200), which project into the barrel, are depicted in aqua stick representation but are not labeled. These hydrophobic residues form van der Waals contacts with neighboring hydrophobic residues. (d) Mechanism of OMP decarboxylation through the formation of a vinyl carbanion. (e) Linear relationship between the Cornette hydrophobicity scale^58^ of the residue at position 123 of Mt-OMPDC and the activation energy (*E*_a_(*k*_cat_)) for the reaction (*R* ^2^ = 0.85), excluding the L123G mutant, which is represented as an inverse triangle and shown for comparison only.

The consensus mechanism for OMP decarboxylation involves a rate-limiting formation of a vinyl carbanion-carbene intermediate, followed by protonation from residue K72 (Figure 1d).^12–16^ A p*K*_a_ analysis of the C-6 proton of enzyme bound product UMP shows a 10-unit reduction when compared to water, consistent with enhanced stabilization of a carbanion-carbene intermediate.^13^ While the proximal K72 moiety may contribute to electrostatic stabilization, D70 and D75′ groups will introduce destabilization. Interestingly, the K72A mutation reduces specific activity by about 5 orders of magnitude but also significantly lowers the dissociation constant (*K*_d_) for UMP by the same extent, indicating that the reduced activity is likely due to product inhibition rather than a loss of transition state stabilization.^17^ One of the most intriguing aspects of the active site is its lack of functional groups that would typically stabilize a local carbanion intermediate. This led to the proposed carbene intermediate that is stabilized by hydrogen bonding from a remote S127 backbone amide in loop 5. However, modifying the amide group of S127 to an ester resulted in only a two orders of magnitude decrease in *k*_cat_, indicating that this single interaction contributes only a small fraction to the overall catalytic enhancement.^18^ As demonstrated in human OMPDC,^19^ substrate distortion results from active-site geometric constraints and hydrogen bonding stabilization between the OMP carboxylate group and the D70 side chain, producing an out-of-plane bending of the carboxylate. Similar distortion has been observed in the binding of analogs such as 6-CN-UMP and 6-N_3_-UMP.^20^ However, structural and mutational analyses indicate that this distortion contributes only ∼3–4 kcal/mol to the overall catalytic enhancement.^10,21^

Investigations based on X-ray derived structures have focused on the roles of the substrate phosphate, ribofuranosyl and pyrimidine moieties in OMPDC’s catalytic proficiency ((*k*_cat_/*K*_m_)/*k*_non_).^21–24^ For instance, the R203A mutation in the phosphate-binding region increases *K*_m_ by approximately 3 orders of magnitude with minimal impact on *k*_cat_.^25^ Similarly, decarboxylation of the truncated substrate 1-(β-D-erythrofuranosyl)­orotic acid (EO) in the presence of phosphite dianion shows a three orders of magnitude increase in *K*_m_, with little effect on *k*_cat_.^26^ These findings suggest that the phosphate group in the OMP primarily enhances substrate binding by lowering *K*_m_, with minimal effect on the unimolecular reaction rate *k*_cat_.

Despite a significant reduction in activation enthalpy in the enzyme-catalyzed reaction compared to that in water, the Mt-OMPDC catalyzed decarboxylation still faces an enthalpic activation barrier of 15.5 kcal/mol after the formation of the enzyme–substrate complex. Crystal structure comparison between the substrate-analog (SA) bound form and the TSA-bound form shows minimal structural differences, with a global RMSD of 0.3 Å (). The interaction distances between key residues and bound molecules differ by ca. ± 0.3 Å in the SA and TSA-bound forms, suggesting that the enzyme undergoes only slight structural change in the presence of a tight-binding inhibitor and arguing against “tight transition state binding” as the explanation of catalysis.

Theories of the reaction rate in the condensed phase differ significantly from gas phase kinetics, ultimately being dependent on the structure and dynamics of the solvent. In particular, the kinetic and potential energy of the solvent must undergo dynamical transfer to the reactant(s), a process that takes place via transient environmental perturbations that create local changes in bond distances, angles and electrostatics required for barrier crossings.^27–29^ Extending these ideas to enzyme catalysis requires the incorporation of the protein scaffold together with its bound and surrounding water molecules into the dynamical solvation sphere.^30,31^ The first step in reaching transition state energies in enzymes is generally attributed to a conformational landscape; comprised of broadly distributed and rapidly interconverting protein substates (ES) with energies for exchange close to the ambient temperature (∼0.6 kcal/mol at RT). The much larger experimental enthalpic barrier for Mt-OMPDC of 15.5 kcal/mol raises the question of how such a large energy gap can be overcome to create the millisecond time scale reactivity of both OMPDC and the majority of thermally activated enzymes.^6,32^

Temperature-dependent hydrogen–deuterium exchange mass spectrometry (TDHDX), when coupled with site-specific mutation that impairs the activation energy of catalysis (*E*_a_(*k*_cat_)), serves as a powerful tool for identifying spatially resolved protein networks, connecting the enzyme’s active site to the solvent bath. These networks are proposed to undergo thermal activation through collisions with solvent, leading to rapid structural and vibrational changes that modulate the active site environment and facilitate barrier crossings of the enzyme–substrate (ES) complex.^31,33,34^ This behavior offers a fundamental mechanism by which enzymes integrate physical processes within their scaffold and solvent interactions to drive chemical transformations at secluded active sites.^34^ TDHDX has been used across various enzyme systems, such as soybean lipoxygenase,^35,36^ dihydrofolate reductase,^37^ murine-adenosine deaminase,^38,39^ enolase,^40^ and catechol O-methyl transferase,^41,42^ to identify reaction-specific networks for thermal energy transmission from solvent to active site. In this study, we report a detailed analysis of TDHDX on a ligand-bound form of wild-type (WT) Mt-OMPDC and a mutant that impairs the activation enthalpy of *k*_cat_, identifying four spatially resolved networks that connect their respective protein–water interfaces to interior positions that surround the bound substrate.

## Results

### Kinetic Analyses of Wild-Type and Mutant Forms of Mt-OMPDC

WT and mutant forms of Mt-OMPDC were expressed and purified following established protocols^5^ with modifications (SI, ). The mutational strategy introduced subtle packing defects by replacing a hydrophobic residue with hydrophobic variants of altered shape and volume. This approach enables us to modify protein flexibility or motion without a significant alteration of electrostatics within the active site. The goal is to evaluate possible correlations between introduced changes in protein flexibility and the primary kinetic parameters *k*_cat_ and *K*_m_ together with the activation energy for *k*_cat_, *E*_a_(*k*_cat_). Enzyme kinetics were performed by spectroscopic monitoring of the product formation (SI, ). A range of mutants were generated to identify the most effective function-impairing mutation, i.e., defined as one that minimally impacts *k*_cat_ (where *k*_cat_ is limited by the rate of the chemical step) and *K*_m_ but significantly alters *E*_a_(*k*_cat_). In the case of Mt-OMPDC, we have targeted residues forming a hydrophobic patch (Figure 1c) within van der Waals contact of the substrate and with each other.

We first interrogated a range of single site hydrophobic side chain alterations, such as Ile68, Ile96, and Val155 (), which in all instances lead to small changes in *E*_a_(*k*_cat_) (ca. ± 1 kcal/mol) along with decreases in *k*_cat_ that for the majority (>80%) were no more than 4-fold, . The generally minor impact on *E*_a_(*k*_cat_) at these sites indicates that the activation energy of Mt-OMPDC is robust and relatively insensitive to random mutations within the hydrophobic side chains throughout the core. We then turned to targeting the residues within van der Waals contact of the substrate and with each other (cf. Figure 1c), identifying informative mutations at Leu123. Conservative changes to the side chain L123 lead to very modest changes in *k*_cat_ and *K*_m_ (2–3 fold), a correlation between *E*_a_(*k*_cat_) and side chain hydrophobicity (Figure 1e), and a 2.2 kcal/mol increase in the *E*_a_(*k*_cat_) for L123A, Table 1 and . The rise in activation energy shows a linear correlation with the hydrophobicity of the substituted residue (L123A, L123I, and L123V) with the exception of the Gly substitution that resembles WT in behavior. The correlation between *k*_cat_ and hydrophobicity is less pronounced (). Residue L123 is part of the hydrophobic patch near the substrate, situated at the start of loop5 and within van der Waals distance of the K72 residue. Based on the presented kinetic analyses, WT and the L123A variant of Mt-OMPDC were selected for an in-depth investigation using single temperature HDX-MS followed by TDHDX.

**Table 1:** Kinetic Parameters of Wild-Type and L123 Mutants of Mt-OMPDC^a^

	*k*_cat_ (s^–1^) at 25 °C	*K*_m_ (μM) at 25 °C	*E*_a_(*k*_cat_) (kcal/mol)
WT	4.3(0.4)	1.4(0.2)	15.5 (0.3)
L123A	1.4(0.1)	3.1(0.3)	17.7(0.3)
L123V	2.3(0.2)	1.4(0.3)	16.1(0.5)
L123I	2.6(0.1)	1.1(0.1)	16.7(0.5)
L123G	3.6(0.1)	2.2(0.2)	15.1(0.3)

a Standard deviation from triplicate measurements in parentheses.

### Single-Temperature HDX Analysis

Single-temperature HDX has been widely used to gain insights into protein–ligand, and protein–protein interactions.^43,44^ HDX measurements at 35 °C were analyzed for Mt-OMPDC, to assess changes in deuterium uptake due to mutation and ligand binding. Detailed protocols for HDX and data collection are provided in the SI, . Briefly, WT­(apo) and L123A­(apo) were directly incubated with deuterated buffer, while WT­(L) and L123A­(L) were preincubated with saturating concentrations of the tight-binding inhibitor 6-azaUMP (), introduced as a TSA,^10,11^ for 30 min prior to exposure to D_2_O. The HDX experiments were conducted across 14 time points (10 s to 4 h) followed by quenching, proteolytic digestion and liquid chromatography–mass spectrometry (LC-MS) analysis. The D-uptake was analyzed using 19 nonoverlapping peptides providing 94% protein sequence coverage ( and ). Experiments were conducted using biological replicates, and the EX-2 condition for HDX was verified by inspection of time-dependent mass envelopes; data were corrected for back-exchange ().

shows comparative, time-dependent D-uptake for peptides from both apo- and ligand-bound forms of WT and L123A at 35 °C. The variant L123A­(apo) is seen to be greatly destabilized in the region at or close to the mutational site (peptides 122–133 and 133–141). Additionally, two peptides at the dimer interface (94–110 and 71–88) show a marked increase in HDX after 20 min, suggesting increased dissociation of the dimeric form of L123A­(apo). The implied destabilization of L123A­(apo) is consistent with *T*_m_ measurements that indicate a *T*_m_ of 60 °C compared to *T*_m_ of 75 °C for WT­(apo) (). With this degree of instability, L123A­(apo) was concluded to be a poor candidate for comparative HDX studies across a wide temperature range.

By contrast, the comparative HDX analysis of L123A and WT in the presence of 6-azaUMP is seen to eliminate the extreme effects observed for apoprotein, bringing the behavior of L123A­(L) at 35 °C closer to WT­(L) for virtually every peptide analyzed (). These observations are supported by an observed 15 °C increase in the *T*_m_ of L123A­(L) to 74 °C, that can be compared to a *T*_m_ of 81 °C for WT­(L) (). On this basis, it was decided to restrict detailed temperature variable HDX comparisons of L123A to WT to the protein–ligand complexes (see Temperature Dependent HDX (TDHDX) Analysis of WT­(L) and L123A­(L) below).

#### Quantitative Analyses of the Impact of Mutation and Ligand Binding on HDX at 35 °C

Differences between D-uptake for L123A and WT as well as the impacts of ligand binding were assessed by estimating the normalized percentage change in D-uptake (Δ*D*(%)) using eq 1:

1 $$\Delta D\left(\%\right)=\frac{D_{L123A\left(apo\right)}-D_{WT\left(apo\right)}}{N_{T}}\times 100$$

where *D*_L123A(apo)_ and *D*_WT(apo)_ represent the D-uptake within a given peptide in L123A­(apo) and WT­(apo), respectively, at given time. *N*_T_ is the total number of exchangeable amides in that peptide. The impact of ligand binding on enzyme (both WT and L123A) was estimated by comparing *D*_E(L)_ and *D*_E(apo)_, which represent the D-uptake within a specific peptide in the presence or absence of a ligand, respectively. Representative Δ*D*(%) values between L123A­(apo) and WT­(apo) after 10 min (intermediate time regime for HDX) and 120 min (slow time regime for HDX) are summarized in and mapped onto the crystal structure for peptides with Δ*D*(%) at a 3σ or higher level of significance in Figure 2a–b; visualization is represented by blue-white-red heatmaps, showing lower (blue) or higher (red) D-uptake, in mutant, respectively. An increase in protein flexibility for L123A­(apo) near the site of mutation is seen at 10 min (Figure 2a) in peptides 122–133 and 133–141, along with some decreased flexibility around the periphery of the protein. While the regions of protein coverage that conform to 3σ differences are somewhat different at the 120 min incubation (Figure 2b), a clear-cut trend of increased exposure at longer times is observed for L123A-derived peptides near the dimer interface (peptides 94–110, 111–121 and 71–88). These results corroborate the conclusions from *T*_m_ measurements of reduced stability for L123A, with the pattern of changes in HDX consistent with the reduced stability at the dimer interface.

**Figure 2:**
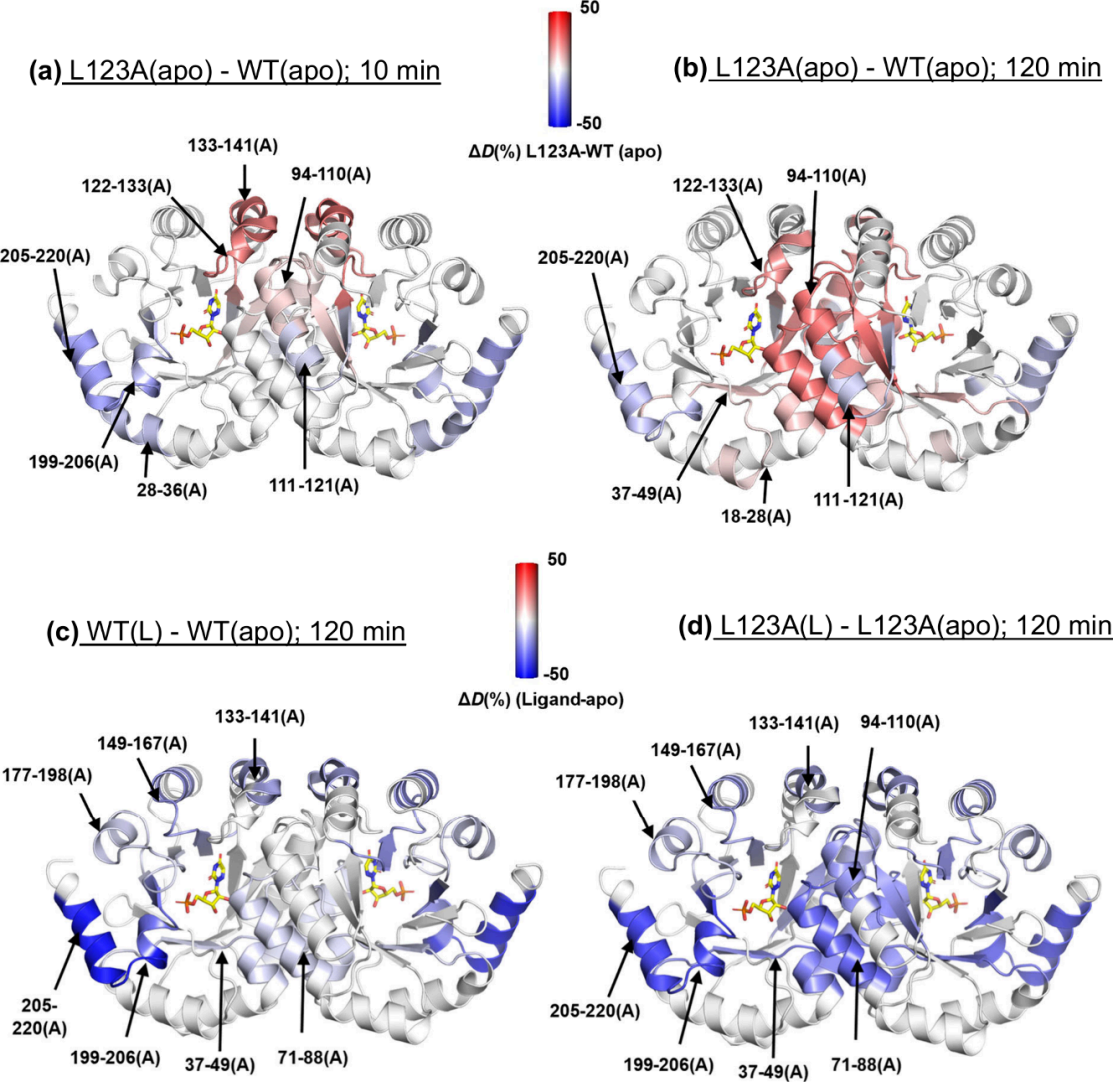
**Single-temperature HDX analysis at 35 °C to assess the impact of mutation and ligand binding on thermal stability of Mt-OMPDC.** Panels (a) and (b) show the effects of the L123A mutation in the apo form, with panel (a) representing normalized percentage change in D-uptake, Δ*D*(%), after 10 min of HDX and panel (b) after 120 min. The Δ*D*(%), calculated using eq 1 (see the text), quantifies differences in D-uptake for each peptide due to the mutation. These changes were mapped onto the Mt-OMPDC crystal structure (PDB: 3G1A) using a blue-white-red gradient, where blue and red indicate decreased and increased D-uptake, respectively, in the mutant (L123A) compared to the wild type (WT). Only changes greater than 0.5 Da and 3σ, were considered, and peptides showing significant changes are marked with arrows only on the A monomer to avoid overcrowding. Panels (c) and (d) display the effects of ligand binding on WT and L123A after 120 min of HDX. These Δ*D*(%) changes were similarly mapped using the blue-white-red gradient, with blue indicating decreased D-uptake (enhanced structuring) and red indicating increased D-uptake (reduced structuring) in the ligand-bound form compared to the apo form.

The direct impact of ligand binding on protection against HDX at a 3σ level of significance is also very informative in this regard (see for the complete tabulation of data for WT and L123A). In Figure 2c–d, we focus on HDX changes at 120 min for WT­(L)-WT­(Apo) and L123A­(L)-L123A­(apo). In both enzyme forms, there is a similar degree of protection from binding of ligand at the periphery of the protein. By contrast, there is a marked increase in protection seen for L123A­(L)–L123­(apo) at the dimer interface peptides 94–110 and 71–88. Despite the absence of definitive 3σ data for comparison to peptides 94–110 in WT­(L)-WT­(apo), protection by 6-azaUMP against structural disruption at the dimer interface in L123A­(apo) is very apparent from the behavior of peptide 71–88.

#### Comparative HDX Patterns for L123A­(L) vs WT­(L) at Single Temperature

The TSA-bound form of WT reflects the conformational dynamics of the catalytically relevant closed states of OMPDC. When HDX differences between L123A­(L) and WT­(L) were analyzed at 35 °C (, ), most peptides showed minimal changes. In the case of peptide 37–49, there is a slight increase at 10 min that disappears after two h, and peptides 60–71 and 122–133 indicate ∼10% increase at 2 h. Similar trends were observed at 50 °C (near *T*_opt_ = 65 °C), with only minor differences after 10 min and 2 h (, ). This analysis highlights the limitation of comparative single-temperature HDX in capturing changes in conformational dynamics from site-specific mutation. The use of TDHDX provides a far more insightful connection between the protein scaffold motion and enzyme activity.

### Temperature Dependent HDX (TDHDX) Analysis of WT­(L) and L123A­(L)

The application of TDHDX in protein structure dynamics has been described.^45^ The goal of TDHDX is to determine local, site-specific changes in activation energies, *E*_a_(*k*_HDX_), for the protein scaffold of a chosen mutant that can be directly compared to the impact of mutation on *E*_a_(*k*_cat_). Sample preparation and HDX time points used in TDHDX analysis are the same as the single-temperature HDX, extended to seven temperatures (SI, ). The D-uptake as a function of time for peptide is analyzed using a three-exponential equation,

2 $$Daltons=N_{T}-Ae^{{-k}_{1}t}-Be^{{-k}_{2}t}-Ce^{{-k}_{3}t}-N_{NE}$$

where *k*_1_, *k*_2_, and *k*_3_ define the fast, intermediate and slow HD exchange rate regimes, respectively, with *A*, *B*, and *C* indicating their respective amplitudes. *N*_T_ represents the total number of exchanging amides, and *N*_NE_ the nonexchanging amides. Boundary conditions for the different HD exchange regimes were determined by initially fitting the D-uptake vs ln­(*T*) data using standard time regimes.^40^ Subsequent manual curation of these exponential fits was used to refine the time-dependent boundaries for slow, intermediate, and fast exchange. The peptide-specific fitting parameters were determined as detailed in , and summarized in . The final fitting parameters obtained for WT­(L) and L123A­(L) are provided in . Due to time constraints in manual quenching of HDX, precise measurement of *k*_1_ is not feasible, and the present studies are focused on the reliably obtained exchange time regimes, *k*_2_ and *k*_3_. The rate constant for HDX, *k*_HDX_, is generally well determined using a weighted average rate constant^38–40^ (eq 3), although in some instances the data may be better analyzed using the temperature dependence of the individual rate constants.^38^

3 $$k_{HDX}=\left(Bk_{2}+Ck_{3}\right)/N_{T}$$

Temperature-dependence of *k*_HDX_ was assessed by performing HDX at seven temperatures (between 15 and 55 °C) for both WT­(L) and L123A­(L). Arrhenius plot analysis of *k*_HDX_ yields *E*_a_(*k*_HDX_) = Δ*H* ^‡^(*k*_HDX_) + RT. Under EX-2 conditions (*k*_closed_ *≫ k*_int_), Δ*H* ^‡^(*k*_HDX_) is comprised of the sum of Δ*H*°_open_ (enthalpy of opening-closing equilibrium; *K*_open_) and Δ*H* ^‡^_int_ (activation enthalpy of the intrinsic HDX rate constant, *k*_int_ *)*. Since *k*_int_ is typically unaffected by a single-site mutation (with the possible exception of the mutation-containing peptide), subtraction of Δ*H* ^‡^(*k*_HDX_) for WT from L123A leads to a direct determination of the impact of mutation on Δ*H*° for *K*_open_.

In this manner, time-averaged TDHDX analysis of WT and the *E*_a_(*k*_cat_)-impaired mutant (L123A) provides a probe for the sought-after relationship between mutation-induced changes in local scaffold flexibility (Δ*H*°) and activation energies for the chemistry at the active site (*E*_a_ *(k*_cat_)). The full set of time and temperature dependencies of D-uptake in WT­(L) and L123A­(L) can be found in . The full set of comparative Arrhenius plots are presented in and *E*_a_(*k*_HDX_) values for each peptide are listed in . In almost every instance, fittings that used weighted average rate constants or individual *k*_*2*_ values yielded comparable results. Out of the 19 peptides, 16 showed significant values for *E*_a_(*k*_HDX_), while the remaining 3 did not exhibit an Arrhenius dependence of the rate. Among these 16 peptides, 8 yielded statistically significant (>2σ) values for Δ*E*_a_(*k*_HDX_).

#### Illustrative Comparison of TDHDX of L123A­(L) to WT­(L) for Peptides 37–49 and 94–110

Figures 3a–b displays HDX data in WT­(L) and L123A­(L) as a function of temperature for peptide 37–49 (representing substrate ribose-binding region). Figure 3c shows the Arrhenius plot for this peptide with *E*_a_(*k*_HDX_) increasing from 9.0 ± 0.9 kcal/mol in WT­(L) to 15.9 ± 1.2 kcal/mol in L123A­(L). The Δ*E*_a_(*k*_HDX_) of 6.9 ± 1.1 kcal/mol, calculated by subtracting the *E*_a_(*k*_HDX_) of WT­(L) from L123A­(L), indicates that mutation increases protein’s rigidity in this region. Similarly, Figures 3d–e shows the D-uptake traces for peptide 94–110 (representing the dimer interface), with the Arrhenius plots presented in Figure 3f. The *E*_a_(*k*_HDX_) is 12.3 ± 1.3 kcal/mol for WT­(L) and 13.5 ± 1.2 kcal/mol for L123A­(L), leading to a statistically insignificant Δ*E*_a_(*k*_HDX_), confirming no impact of mutation on local unfolding dynamics at the dimer interface when a ligand is present.

**Figure 3:**
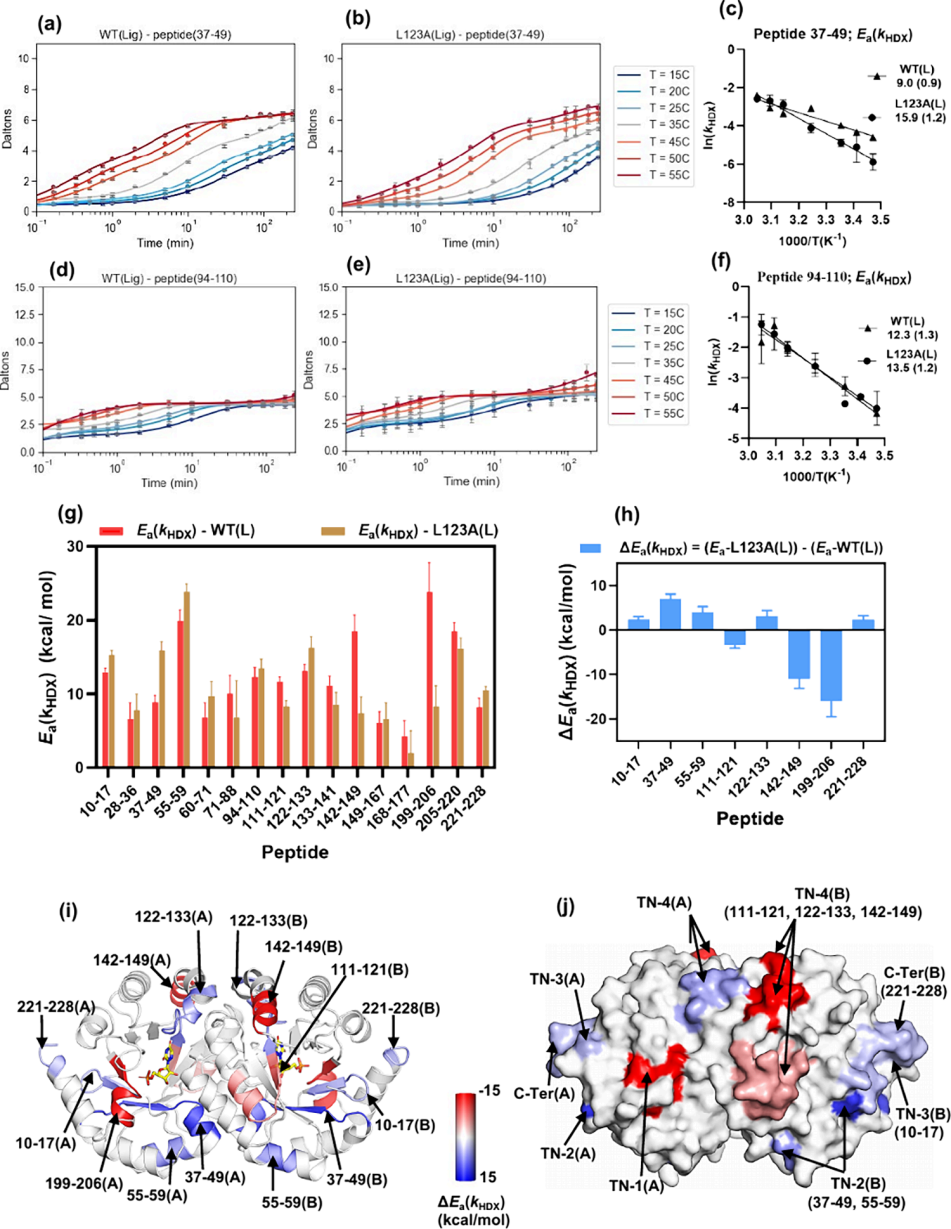
**Temperature-dependent HDX-MS analysis of WT­(L) and the** ***E***_**a**_ **(*k***_**cat**_ **) impairing mutant L123A­(L) variants of Mt-OMPDC.** Panels (a) and (b): D-uptake as a function of time and temperature for peptide 37–44 (representing ribose-binding motif) in the TSA-bound forms of native WT­(L) and mutant L123A­(L), respectively. (c) Arrhenius plot of ln­(*k*_HDX_) vs 1000/T for peptide 37–44. Panels (d) and (e): D-uptake as a function of time and temperature for peptide 94–110 (representing the dimer interface) in the TSA-bound forms of WT­(L) and L123A­(L), respectively. (f) Arrhenius plot of ln­(*k*_HDX_) vs 1000/T for peptide 94–110. (g) Bar graph representing the HDX activation energy, *E*_a_(*k*_HDX_), values of peptides in WT­(L) and L123A­(L). Error analysis for panels (c), (f), and (g) was performed by calculating activation energies (*E*_a_(*k*_HDX_)) from Arrhenius plots (ln­(*k*_HDX_) vs 1000/*T*) across seven temperatures using data from biological replicates; standard deviations from linear regression fits were used to assess statistical significance. (h) Bar graph representing Δ*E*_a_(*k*_HDX_), calculated as *E*_a_(*k*_HDX_) for (L123A­(L)) minus *E*_a_(*k*_HDX_) for WT­(L)). Only peptides showing Δ*E*_a_(*k*_HDX_) > 2σ, are shown, where σ is the standard propagated error from replicate-based activation energy difference. (i) Cartoon representation of the Mt-OMPDC homodimer, highlighting peptides with significant Δ*E*_a_(*k*_HDX_). Regions with changes in Δ*E*_a_(*k*_HDX_) are shown using a red-white-blue gradient, with blue indicating positive Δ*E*_a_(*k*_HDX_) and increased rigidity in mutant, and red indicating negative Δ*E*_a_(*k*_HDX_) and increased flexibility in L123A­(L) relative to WT­(L). Peptides with significant changes in Δ*E*_a_(*k*_HDX_) are marked with arrows. (j) The space-filled model highlights regions with Δ*E*_a_(*k*_HDX_) changes using a red-white-blue gradient, revealing dynamic thermal networks (TN) connecting solvent-exposed regions to the active site. Thermal Network 1 (TN-1) corresponds to the phosphate-binding loop. Thermal Network 2 (TN-2) represents the sugar-binding region. Thermal Network 3 (TN-3) links TN-1 and TN-2 to facilitate synergistic interactions. Thermal Network 4 (TN-4) represents the catalytic loop. It is worth noting that TN-1 is present in both monomers but only shown in one monomer (TN-1 in A) as is located on the backside of the surface of monomer B.

#### Distributed Differences in Δ*E*_a_(*k*_HDX_) for WT­(L) and L123A­(L)

The 16 peptides with measurable *E*_a_(*k*_HDX_) values are summarized in Figure 3g and the Δ*E*_a_(*k*_HDX_) values for 8 peptides exceeding 2σ are shown in Figure 3h. These peptides are mapped onto the structure of Mt-OMPDC using a blue-white-red gradient heatmap (Figure 3i), highlighting spatially resolved regions with altered enthalpy of local unfolding due to the *E*_a_(*k*_cat_)-impairing mutant. In this heatmap, blue indicates increased rigidity in the mutant relative to WT, while red indicates increased flexibility. Table 2 summarizes the secondary structure composition and key catalytic residues for peptides with significant changes.

**Table 2:** HDX Activation Energy (*E*_a_(*k*_HDX_)) for WT and L123A Mutant Mt-OMPDC along with Their Differences Δ*E*_a_(*k*_HDX_) and Associated Structural Features, Thermal Network Assignments, and Key Interactions^a^

Peptide	*E*_a_(*k*_HDX_); WT(L)	*E*_a_(*k*_HDX_); L123A(L)	Δ*E*_a_(*k*_HDX_); L123A(L) – WT(L)	Properties
10–17 (TN-3)	12.9 (0.6)	15.3 (0.6)	2.4 (0.6)	The β1 strand of this peptide forms backbone H-bonds with the β8 strand of peptide 199–206, TN-1 (R14···I199, I16···I199 and I16···V201) and with the β2 strand of peptide 37–49, TN-2 (I17···T40 and I17···K42). It also undergoes a side-chain to backbone H-bond with the C-terminal peptide (R14···L225). See Figure 4a.
37–49 (TN-2)	9.0 (0.9)	15.9 (1.2)	6.9 (1.1)	This peptide contains K42 that H-bonds to the ribose ring of substrate; it also forms backbone hydrogen bonds to TN-3 (see above). A van der Waals interaction (L47···I55) connects peptide 37–49 to peptide 55–59.
55–59 (TN-2)	19.9 (1.4)	23.8 (1.0)	4.0 (1.3)	This solvent-exposed peptide contains α2 and undergoes a van der Waals contact with peptide 37–49 (see immediately above).
111–121 (TN-4)	11.7 (0.6)	8.3 (0.8)	–3.4 (0.7)	The β5 strand of TN-4 extends downward toward the α4 region, and lies adjacent to the mutation site (L123) in peptide 122–133. See Figure 3i.
122–133 (TN-4)	13.1 (0.9)	16.3 (1.5)	3.2 (1.3)	The loop5 of peptide 122–133 contains the mutation site L123 and the intermediate-stabilizing residue S127. See Figure 4b. It is also in van der Waals contacts with peptide 142–149 (see immediately below).
142–149 (TN-4)	18.5 (2.4)	7.4 (2.2)	–11.1(2.2)	This peptide includes the α5 region that forms van der Waals contacts with peptide 122–133 (I142···L122) as well as across the dimer interface (I142···F134′). See Figure 4b.
199–206 (TN-1)	23.9 (4.0)	8.4 (2.8)	–15.5 (3.5)	This peptide contains the phosphate-binding residue R203 and forms backbone H-bonds with peptide 10–17, TN-3 (see above).
221–228 (C-terminus)	8.1 (1.2)	10.5 (0.5)	2.3(0.9)	The solvent-exposed C-terminus forms an H-bond with peptide 10–17, TN-3 (see above). Since TN-3 connects TN-1 and TN-2, this interaction effectively links the C-terminus to the active site.

a Values in parentheses indicate standard deviation obtained from biological replicates using linear regression of Arrhenius plots. The table includes only peptides with Δ*E*_a_(*k*_HDX_) > 2σ.

Peptide 199–206, containing the R203 side-chain that interacts with the phosphate group of the substrate (phosphate-binding region), shows the largest reduction in *E*_a_(*k*_HDX_) (−15.5 kcal/mol), implying significantly increased flexibility in this region due to mutation. While mutations within the phosphate-binding region typically raise *K*_m_ for substrate by 2–3 orders of magnitude,^25^ the L123A mutation causes only a 2-fold increase, suggesting minimal impact of this mutation on the ground state ensemble of ES complexes. By contrast, the observed impact of L123A on *E*_a_(*k*_HDX_) within this region indicates a mutation-induced disruption of protein packing that is expected to reduce the level of efficient thermal energy transfer from the solvent to the bound substrate that is intrinsic to the WT enzyme. Peptide 199–206 is seen to be exposed to the solvent surface, connecting the solvent bath to the active site (Figure 3j) and is thus identified as thermal network 1 (TN-1) in Mt-OMPDC.

The structurally adjacent peptide 10–17 at the N-terminus shows a small decrease in flexibility, Δ*E*_a_(*k*_HDX_) = 2.6 kcal/mol, as does the C-terminal peptide 221–228, Δ*E*_a_(*k*_HDX_) = 2.3 kcal/mol. The β1-strand in peptide 10–17 forms backbone H-bonds with the β8-strand in peptide 199–206 (TN-1), creating a structural link between these regions. Additionally, peptide 10–17 engages in H-bonding with the C-terminal peptide 221–228, thus establishing a connection between TN-1 and the solvent-exposed regions at the N and C- termini of the protein (Figure 4a).

**Figure 4:**
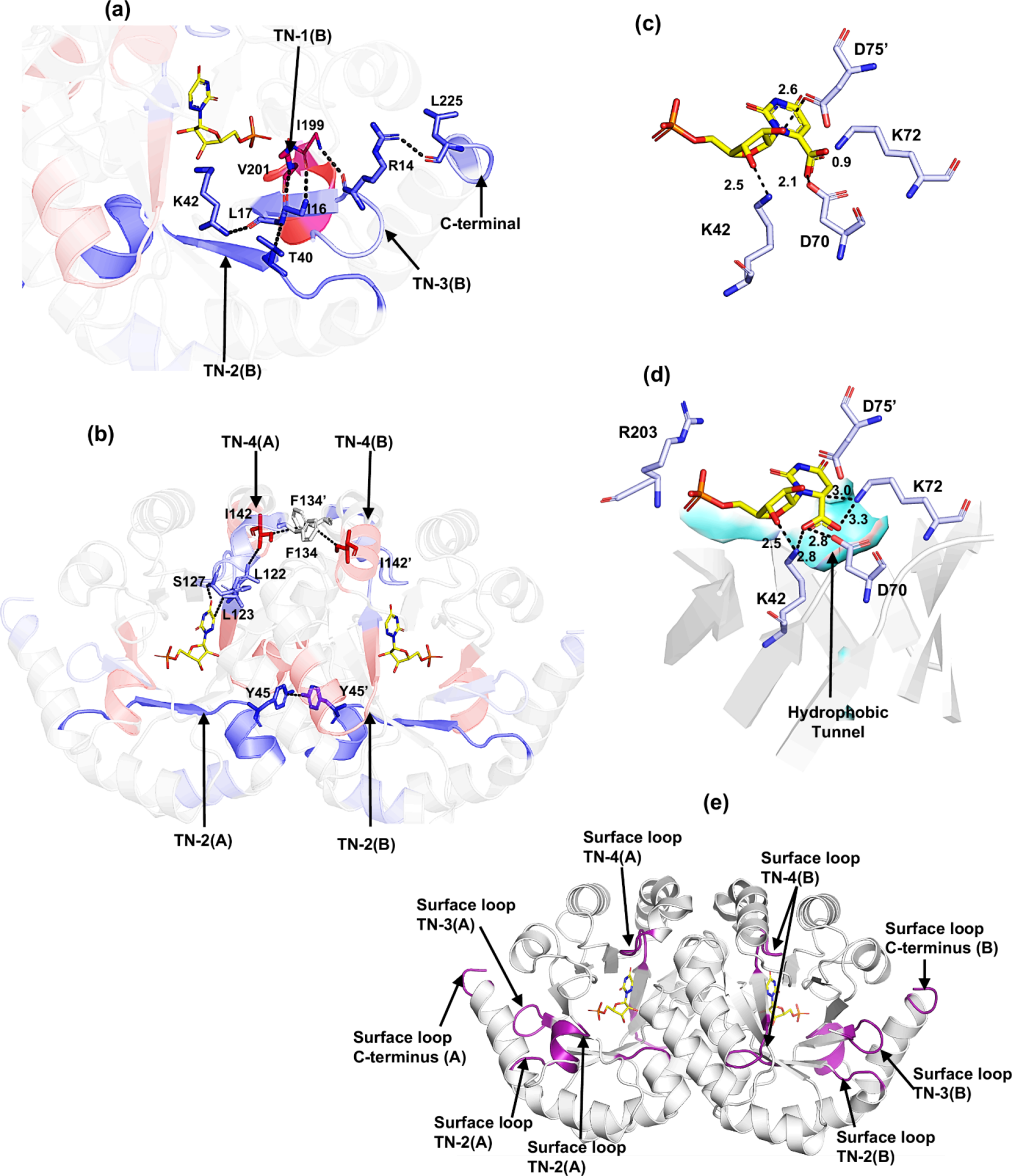
**Contributions from intra- and intersubunit thermal networks drive thermal activation of catalysis and barrier crossing in Mt-OMPDC.** (a) Intrasubunit interactions between thermal-networks: Peptide 10–17 (TN-3) bridges TN-1 and TN-2 through H-bonding, enabling synergistic activation. Hydrogen bonding between R14 and L225 connects the N- and C-termini. (b) Intersubunit interactions, F134–I142′ (linking TN-4 and TN-4′) and Y45–Y45′ (connecting TN-2 and TN-2′), may facilitate coordinated action of thermal networks between subunits, alongside intrasubunit connectivity of TN-2 and TN-4. The color code in (a, b) is the same as described in Figure 3 for changes in Δ*E*_a_(*k*_HDX_) (red to blue goes from negative to positive). (c) The active site of OMPDC was modeled using the 6-azaUMP bound crystal structure (PDB: 3G1A). To represent the substrate-bound active site, OMP was positioned in place of 6-azaUMP into this structure. In this initial configuration, where the carboxylate group is in the plane of the pyrimidine group, it sterically clashes with catalytic residue K72 (0.9 Å) and undergoes a very close proximity to D70. (d) Modeling of the bound OMP in OMPDC to avoid steric repulsion with K72. The C6–C7 bond was rotated while keeping other residue positions fixed, with the criterion that the side chains K42, D70, K72, and D75′ remain > 2 Å apart. This distorts the carboxylate out of the pyrimidine ring plane into a hydrophobic patch, while retaining a close (H-bonding) distance for D70.^19^ (e) Cartoon representation highlighting the spatial distribution of loop elements (purple) within the identified thermal networks in Mt-OMPDC. Surface-exposed loops of the C-terminus, TN-3 (N-terminus), and TN-2 are interconnected. Loops associated with TN-1 are solvent-exposed but connect to TN-2 through internal interactions rather than surface interactions.

Peptide 37–49 contains K42, a key residue for substrate binding and catalysis, that forms H-bonds with the substrate ribose 3′OH, and D70. K59A mutagenesis in Sc-OMPDC, equivalent to K42 in Mt-OMPDC, decreases *k*_cat_ by 100-fold and increases *K*_m_ by 1000-fold.^46,47^ A Δ*E*_a_(*k*_HDX_) of 6.9 kcal/mol for peptide 37–49 following the introduction of L123A indicates increased rigidity near the sugar-binding region. Decreased flexibility is expected to perturb the ability of enzyme to achieve activated-complex configurations in a manner analogous to the perturbation that arises from increased flexibility within the phosphate-binding region. Enhanced rigidity of K42 (37–49) is also likely to disrupt the dynamic positioning of D70, a key residue that contributes to catalysis by enforcing substrate carboxylate distortion through a productive hydrogen bond with the carboxylate group. Disruption in this position may be a major source of the increased value of *E*_a_(*k*_cat_) for L123A. Peptide 55–59 is in van der Waals contact with peptide 37–49 and also shows increased rigidity (Δ*E*_a_(*k*_HDX_) = 4.0 kcal/mol). Though not directly interacting with the substrate, its dynamics may impact peptide 37–49 and indirectly influence the thermal initiation of bond cleavage. Space filling representation reveals that peptides 37–49 and 55–59 connect the solvent bath to the active site (Figure 3j) and are ascribed to thermal network 2 (TN-2) in Mt-OMPDC. Note that the marked rigidification of peptide 37–49 in L123A was apparent from single-temperature analysis (). The β1-strand in peptide 10–17 at the N-terminus, which interacts with peptide 199–206 (TN-1) and the C-terminus, also forms backbone H-bonds with the β2-strand in peptide 37–49 (TN-2) (Figure 4a). This establishes a connection between TN-2 and the exposed C- and N-termini, leading to the designation for the peptide 10–17 as a separate thermal network 3 (TN-3) that coordinates the behavior of TN-1 and TN-2.

Peptide 122–133 includes loop5 and the mutational site L123, which is part of a hydrophobic cluster, a pathway for CO_2_ release, and includes S127 that is proposed to stabilize the vinyl carbanion/carbene intermediate.^18^ TDHDX shows a 3.2 kcal/mol increase in *E*_a_(*k*_HDX_) for L123A­(L) relative to that for WT­(L), indicating increased local rigidity that may impair thermally initiated vinyl intermediate stabilization and contribute to the elevated *E*_a_(*k*_cat_) for L123A. Peptide 142–149 is in van der Waals contact with 122–133 in both monomers (Figure 4b). Very significantly, this region shows a large decrease in the Δ*E*_a_(*k*_HDX_) by −11.1 kcal/mol. A second region adjacent to L123, peptides 111–121, also shows a decrease in Δ*E*_a_(*k*_HDX_) by −3.4 kcal/mol. The increased flexibility of 142–149 and 111–121 is also expected to impair contributions of thermally activated dynamics within loop5. All three peptides are solvent-exposed (Figure 3j) and constitute the thermal network 4 (TN-4) in Mt-OMPDC.

## Discussion

### Impact of Temperature on Mt-OMPDC Stability

The original goal of performing a full TDHDX comparative analysis of WT­(apo) and L123A­(apo) forms of Mt-OMPDC proved untenable due to instability of the variant at elevated temperatures. This is supported by a large reduction in *T*_m_ for L123A­(apo) to 60 °C relative to a *T*_m_ of 75 °C for WT­(apo). As anticipated, addition of tight-binding 6-azaUMP elevated *T*_m_ for both WT­(L) and L123A (L), to values of 74° and 81 °C, respectively, where the highest temperature used for TDHDX was 55 °C.

Comparisons of patterns of HDX at 35 °C and 120 min for L123A­(apo) and WT­(apo) (Figure 2b) indicate the regional loss of WT structure at the dimer interface of L123A, implicating an enhanced proclivity to dimer dissociation as a primary source of thermal instability in L123A. This is supported by the multiple temperature HDX analyses of apo forms (), where the L123A­(apo) variant shows a continuous rise in D-uptake as a function of both time and temperature for the peptides representing the dimer interface (71–88, 94–110 and 142–149). By contrast, the WT­(apo) shows none of this behavior, consistently plateauing in D-uptake with increased time and elevated temperature (). Significantly, thermally induced, functionally correlated changes in protein flexibility for L123A­(L) relative to WT­(L) are found to lie almost exclusively outside of the dimer interface, with the exception of a single amino acid within peptides 142–149 and 37–49 respectively (Figure 3i and Figure 4b), implicating different origins of observed temperature responsive behavior for enzyme stability vs function. A full understanding of the molecular origins of thermostability in WT Mt-OMPDC will require further investigation.

### Understanding the Temperature-Dependent Activation of OMPDC from the Perspective of Environmental Reorganization

OMPDC achieves a ca. 17-order catalytic enhancement of the unimolecular decarboxylation of OMP, and it is considered one of the most challenging enzyme reactions to fully rationalize. The literature indicates extensive analyses, with a focus on the contribution of interactions between specific residues and components of substrate on the enhancement of *k*_cat_ and the corresponding reduction in the free energy of activation, Δ*G* ^‡^.^19,48–50,52^ This focus on Δ*G* ^‡^ derives from Pauling’s proposal that differential binding affinity of a transition state relative to substrate drives enzymatic reactions.^53^ However, X-ray crystallography reveals only slight conformational differences between the ES and ETS complexes of Mt-OMPDC (), indicating that static structures are unable to capture the physics that underlies the conversion of the ground state structure to its activated complex.^54,55^

In this work, we turn our focus to the dramatic decrease in the Δ*H* ^‡^ of the OMPDC reaction relative to its solution reaction (Figure 1a), further noting the dominant importance of reduced enthalpic barriers for the majority of enzyme-catalyzed reactions.^3,6,7^ Earlier studies have shown that studies of TDHDX, when performed with enzyme variants that display changes to *E*_a_(*k*_cat_), provide a spatial map of networks that participate in thermal energy transport from solvent to the reactive bond(s) of bound substrates.^35–41^ It is generally recognized that the catalytic competence of ES complexes will be critically dependent on the inherent flexibility of protein scaffolds that support a family of equilibrating protein substates with differing abilities to undergo barrier crossings. This property is most commonly attributed to a conformational landscape composed of a high density of protein substates with small energetic differences (RT ∼ 0.6 kcal/mol). This formalism recognizes the inherent importance of the surrounding protein scaffold in accelerating reaction rates, while leaving open the question of the physical process whereby enzymes are able to overcome enthalpic barriers that are dominantly in the range of 10 kcal/mol.^3,6,7^ This value for enthalpic barriers is further elevated in the case of thermophilic enzymes, as seen in the case of Mt-OMPDC where Δ*H* ^‡^ = 15.5 kcal/mol.

In condensed phase reactions, activated complex formation depends on diffusional encounters of reactants with each other together with subsequent alterations in solvent structure and dynamics that transfer energy to preorganized ground state encounter complexes. The role of environmental reorganization for chemical activation in the condensed phase has been formalized by Marcus theory, which recognizes solvent reorganization (λ) as the primary kinetic barrier, creating an environment complementary to and capable of stabilizing changes in bond distances, bond angles and electrostatics that are prerequisites for functional barrier crossings.^27,28^ Marcus theory also differentiates environmental reorganization from the preorganization process that brings reactants into close proximity. In enzymatic reactions, the latter is provided by a sequestered active site that is susceptible to sampling among a large number of equilibrating ES ground states.

Critically, the transitioning of this family of stable ground state ES complexes to activated complexes is distinctive, relying on high-energy structures that occur transiently and repeatedly within selective regions of the protein scaffold and its accompanying shell of bound water.^56,57^ Over the past decade, carefully designed experimental studies of a number of different enzyme systems have uncovered both the spatial and temporal properties of such protein scaffold reorganization, formulating methods to filter out nonessential dynamics from functionally relevant protein motions that promote activated complex formation.^31,34^ The first step in such studies involves spatial resolution of function-related protein motions, as afforded by TDHDX.

### Complex Thermal Networks Function in Mt-OMPDC

From the TDHDX comparison of WT Mt-OMPDC to its *E*_a_(*k*_cat_) altering variant L123A, we have identified regions of protein that connect separate protein/solvent interfaces to key elements of the enzyme’s active site structure, Figure 3i–j. Four distinct regions have emerged as spatially unique thermal networks (TNs): TN-1, terminating at the substrate phosphate-binding position, TN-2, terminating at the substrate ribose-binding region, TN-3 that connects TN-1 and TN-2, and TN-4 that includes loop5 and contains Ser127 that stabilizes the decarboxylated carbene-carbanion intermediate, Figure 1d. Each is represented in Figure 4a–b.

**TN-1**: Substrate binding induces loop7 closure through a Q185/phosphate and additional interactions (), isolating the active site from solvent and achieving a catalytically competent state. Loop7 also interacts noncovalently with loop5 and the phosphate-binding region (R203). Although previous studies suggest that loop7 length affects the catalytic activation barrier in mesophilic vs thermophilic forms,^5^ our TDHDX analysis of Mt-OMPDC does not identify loop7 as part of a thermal network. In an earlier TDHDX study of the enzyme enolase, the behavior of an active site loop closure was similarly differentiated from regions of the protein susceptible to mutation-induced changes in protein flexibility.^40^

Active site analysis indicates that a planar carboxylate group within the OMP faces steric constraints from D70, and K72. (Figure 4c). To minimize these clashes, the carboxylate group undergoes some distortion from the pyrimidine-ring plane, positioning itself near a hydrophobic pocket that provides an ideal site for CO_2_ release,^19^ (cf. Figure 4d). Similar out-of-plane bending is observed in crystal structures of OMPDC with other substrate analogs, suggesting a conserved mechanism that depends on effective substrate alignment to enhance catalytic activation.^20,59^ We propose that thermal activation in the phosphate-binding region (TN-1), modulated by R203-phosphate interactions, further enhances this distortion, straining the adjacent C6–C7 bond and guiding the carboxylate into the adjacent polar-hydrophobic interface (Figure 4d) as a key step in facilitating bond cleavage.

**TN-2**: Similarly, thermal activation in the substrate ribose-binding region (TN-2) influences the K42-ribose ring interaction, thereby reinforcing the substrate’s carboxylate group dynamic positioning at a polar-hydrophobic interface. Additionally, K42 in TN-2 may form a hydrogen bond with D70 in the catalytic tetrad, with the expectation that TN-2 activation will adjust the positioning of D70, K72, and D75′ to fine-tune the active site’s electrostatic environment (Figure 4d). The action of TN-1 and TN-2 in each monomeric unit of Mt-OMPDC is coordinated through their interaction with the β1-strand of peptide 10–17, assigned as TN-3 that also interacts with C-terminal peptide 221–228 residing at a protein/water surface.

**TN-3**: TN-3 connects TN-1 and TN-2 through hydrogen bonding interactions (Figure 4a and Table 2), forming an extended network that links two distinct regions of the substrate, the phosphate (TN-1) and ribose (TN-2) moieties, with an additional solvent interface. We note that while TN-3 itself is near the solvent surface, it lacks a direct interaction with the bound substrate itself. We propose that networks TN-1 and TN-2, undergo more coordinated dynamic activation as a result of a synergistic interaction with TN-3, that increases the probability of optimal positioning of multiple regions of substrate for C–C bond cleavage. The resulting high energy, transient positioning of the substrate facilitates the release of CO_2_ into a hydrophobic pocket at a polar-hydrophobic surface, to form the proposed carbanion-carbene intermediate (Figure 1d).

**TN4**: The carbene intermediate is stabilized by a H-bond from the backbone amide of S127 in loop5 that is identified within TN-4. Thermal activation of TN-4 will modulate the H-bonding distance between the S127 N–H and O4 of the carbene, directly governing the stabilization energy of this catalytic intermediate (Figure 4b). In Mt-OMPDC, hydrophobic residues from almost all β-strands (except β2) extend into the central barrel, forming a continuous hydrophobic tunnel (aquasurface, Figure 4d). This tunnel, built from strategically arranged hydrophobic residues, establishes a network of isoleucine, leucine, and valine (ILV) side chains, which are involved in van der Waals contacts and identified through a contact-based structural unit algorithm^60^ (). The ILV network within the hydrophobic tunnel intricately connects all four identified thermal networks: TN-1 (containing I200), TN-3 (containing I16), and TN-4 (containing L123). Notably, TN-2 (containing K42) engages in van der Waals interactions with I16 of TN-3 and I200 of TN-1. Thus, the ILV network establishes a cohesive pathway that can facilitate a concerted action of all four thermal networks to generate activated complexes. In addition to intrasubunit connections between thermal networks, interactions such as F134–I142′ (connecting TN-4 and TN-4′) and Y45–Y45′ (connecting TN-2 and TN-2′) may further contribute some intersubunit motions to the activation process (Figure 4b).

We conclude that the ability of the OMPDC to reduce its enthalpic barrier for C–C bond cleavage by ca. 28 kcal/mol relative to the corresponding solution reaction (Figure 1a) is a result of a protein scaffold that has evolved a preorganized active site in close proximity to multiple, interconnected thermal energy networks; the latter drive a thermally activated environmental reorganization process that creates transient complementary of the enzyme’s active site to the altered bond angles and electrostatic characteristics of substrate at its position of barrier crossing.

### Comparison of Thermal Networks in OMPDC to Other TIM Barrel Enzymes

The protein networks identified in Mt-OMPDC provide a fourth comparative example within the TIM barrel family of enzymes.^61^ The first distinctive feature of Mt-OMPDC is a mixed pattern of “more flexible/more rigid” in the regions of loop 5 and the substrate phosphate-binding region following insertion of the L123A (cf. Figure 3i–j). A second feature is the large number of peptides, eight out of a total of 16 analyzed, that are altered in TDHDX by a single site substitution of Leu to Ala (Table 2). A third property is the detection of four distinctive thermal networks, differentiating OMPDC from the other TIM barrel enzymes thus far characterized by TDHDX. As summarized in Figure 5 the TIM barrel enzymes murine adenosine deaminase, yeast enolase, and human catechol-O-methyltransferase, each reveal two thermal networks, separated by ca. 180°, 0° and 90°, respectively; in all cases, these converge at the reactive carbon center within the active site.^38–41^

**Figure 5:**
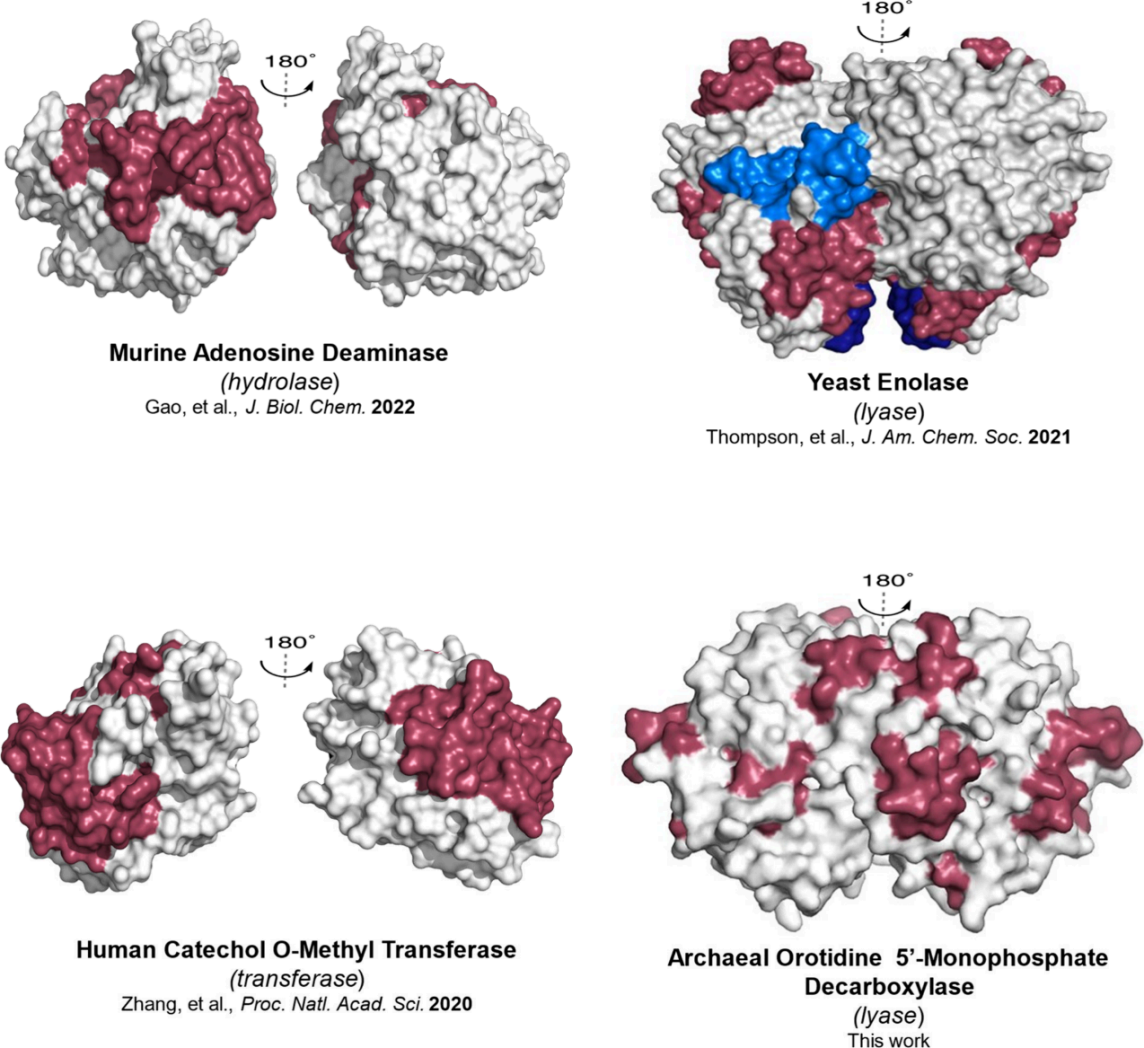
**Thermal energy network comparison among TIM barrel enzymes.** The final TDHDX-derived energy transfer pathways in murine adenosine deaminase (m-ADA),^39^ yeast enolase (Sc-ENO),^40^ and human catechol-O-methyltransferase (Hs-COMT)^41^ each consist of two distinct networks. In contrast, for archaeal OMP decarboxylase (Mt-OMPDC), which catalyzes one of biology’s most challenging reactions, four synergistic thermal energy networks have been identified. In every instance, these networks converge at the reactive bond of the substrate within the respective active site. The thermal energy network figure for m-ADA adapted from ref 39. Available under a CC-BY 4.0 license. Copyright 2022 Gao et al. The thermal energy network figure for Hs-COMT adapted from ref 41. Copyright 2020 PNAS. The thermal energy network figure for Sc-ENO adapted from ref 40. Copyright 2021 American Chemical Society. The network for Mt-OMPDC is the result of this work.

We propose that the extensive thermally activated regions identified in Mt-OMPDC relative to the other TIM barrel enzymes studied are the result of enzyme adaptation to accommodate an enzyme reaction that requires such an enormous enthalpic reduction from the uncatalyzed reaction. The studied members of the TIM barrel family are further differentiated from the enzyme lipoxygenase, comprised of a different two domain structure.^30^ Lipoxygenases have been shown to catalyze a relatively “simple” C–H bond cleavage through quantum mechanical H-tunneling. In this instance, a single thermal energy network appears sufficient to achieve a simultaneous transient reduction of the hydrogen donor–acceptor distance and alteration of Δ*G*° needed to support efficient wave function overlap.^36^

### Integrating Thermal Networks into Dynamic Models for Enzyme Activation

A major mechanistic question is how motions at protein–solvent interfaces may propagate through enzyme networks to promote active site environments capable of rapid barrier crossings. Previous work from this lab on SLO^56^ and m-ADA^57^ extended TDHDX studies to include temporal probes of functionally relevant protein motions. In these instances, dynamic Stokes shift analyses, using fluorophores appended to the protein surface at spatially identified networks revealed a 1:1 relationship between the thermal activation barrier for reorganization at a protein/solvent interface (subnanosecond to picosecond time scale) and the active site reorganization that transforms reactant to product on a slower ms scale. Time differences between the measured kinetic parameters are ascribed to different probabilities of a shared protein restructuring supporting the different reaction outcomes.^61^

In SLO, its single thermal network facilitates long-range communication over ∼20 Å, channeling the entire activation energy for catalysis from the solvent to the active site. In m-ADA, enzymatic activation relies on more complex interactions from two networks originating on opposite faces of the protein structure and converging at the active site. In both instances, protein surface loops are identified and proposed as the dynamic feature that initiates rapid conformational shifts in response to interactions of bulk solvent with the protein/solvent interface. Loop dynamics have previously been shown to govern critical aspects of enzyme function, including substrate binding, active site desolvation, intermediate stabilization, turnover rates, specificity, allostery, and thermal adaptation between mesophilic enzymes and their psychrophilic and thermophilic homologues.^62–70^ In this context, Figure 4e highlights the loops identified to be present at the termini of TN’s1–4 in Mt-OMPDC. Ongoing studies of Mt-OMPDC are focused on obtaining temporal resolution of ns-ps motions through Stokes shift studies of loop-specific chromophores, analogous to the approaches pursued with SLO^56^ and m-ADA.^57^

To conclude, while the importance of protein dynamics in enzyme function is well recognized, identifying functionally relevant motions amidst numerous other movements remains challenging. During the past decade, developed experimental methods have led to the identification of protein networks as a primary component in anisotropic energy transfer to effect productive reaction barrier crossings in enzyme-catalyzed reactions.^34,56,57^ Extending this approach to Mt-OMPDC, which catalyzes one of biology’s most challenging reactions, has uncovered an unusually complex network for thermal energy transfer. The results strongly suggest a correlation between the difficulty of the catalyzed reaction and the number of multiple site-specific conduits within the corresponding protein scaffold.

*De novo* enzyme design, which has traditionally focused on refining active site interactions, has struggled to match the efficiency of natural enzymes^71,72^ due to the limited integration of dynamical components into design principles. Incorporating protein dynamics into enzyme design is challenging when functionally relevant motions are dispersed across the scaffold. However, the identification of spatially resolved thermal networks across enzyme systems offers a promising strategy to target and tune site-specific scaffold dynamics in relation to active site residue and substrate positionings. One possible strategy will be to integrate thermal networks into de novo designed enzymes with established preorganized active sites, thereby reducing the reorganization energy of a catalyzed reaction through efficient energy flow enabled by the coordinated action of thermal networks. Alternatively, identified intrinsic thermal networks within a given scaffold can be utilized to guide the placement of new active site residues with the capability to act on altered substrates and to effect different chemical outcomes. This integrated approach couples active site preorganization with scaffold-encoded dynamic energy flow, reducing the energetic cost of reorganization and facilitating barrier crossing during catalysis.

## Supplementary Material





## Notes

Experimental methods, structural comparisons, enzyme kinetics, single- and temperature-dependent HDX data on OMPDC, including figures, tables, and Python scripts for analysis (PDF)
HDX summary table, WT­(L) data, L123A­(L) data, L123A­(apo) data (XLSX)
