# Supplementary material for: Dynamically Interacting Protein Networks Provide a Mechanism to Overcome the Enormous Intrinsic Barrier to Orotidine 5′-Monophosphate Decarboxylation
Source: ACS Cent Sci. 2025 Jul 11;11(8):1377–90. doi: 10.1021/acscentsci.5c00590 (PMC12395307; doi:10.1021/acscentsci.5c00590)
Supplement: Supplementary file 1 [file oc5c00590_si_001.pdf]

Supplementary Information

Dynamically Interacting Protein Networks Provide a Mechanism to Overcome the Enormous Intrinsic Barrier to Orotidine 5'-Monophosphate Decarboxylation

Pankaj Dubey,[1,2] Anish Somani,[2] Jessica Lin,[3] Anthony T. Iavarone,[1,2] Judith P. Klinman[1,2,4*]

[1]California Institute for Quantitative Biosciences, University of California Berkeley; Berkeley, California 94720, United States.
[2]Department of Chemistry, University of California Berkeley; Berkeley, California 94720, United States.
[3]Department of Bioengineering, University of California Berkeley; Berkeley, California 94720, United States.
[4]Department of Molecular and Cell Biology, University of California Berkeley; Berkeley, California 94720, United States.

*Corresponding author. Email: klinman@berkeley.edu

Table of Contents

Materials and Methods

Section-1) Materials

Section-2) Mt-OMPDC's Expression and Purification

- S2a) Recombinant construction of wild-type and variants of Mt-OMPDC
- S2b) Expression and purification of wild-type and variants of Mt-OMPDC

Section-3) Enzyme Kinetics, Inhibition and Thermal Stability Assays

- S3a) Enzyme kinetics
- S3b) Inhibition constant (K_i) measurement of 6-azaUMP
- S3c) Melting temperature (T_m) measurement of wild-type and L123 constructs of Mt-OMPDC

Section-4) HDX-MS Method and Data Collection

- S4a) Peptide library generation using tandem mass spectroscopy
- S4b) HDX sample preparation
- S4c) HDX measurement using liquid chromatography-mass spectrometry (LC-MS)
- S4d) Back-exchange experiment
- S4e) HDX mass spectrometry raw data analysis

Section-5) HDX Data Analysis at Single-temperature and Multiple-temperature.

- S5a) Single-temperature HDX
- S5b) TDHDX of WT(L) and L123A(L); $E_a(k_{HDX})$ analysis
- S5c) TDHDX of WT(apo) and L123A(apo) forms; T_{melt} HDX analysis

Figures

Figure S1 Structural Comparison of Substrate-Analog and Transition-State-Analog Bound Forms of Mt-OMPDC

Figure S2. Arrhenius plot ($E_a(k_{cat})$) of wild-type Mt-OMDPC and L123 mutants.

Figure S3. Relationship between hydrophobicity scale and catalytic rate (k_{cat}).

Figure S4. Hydrophobic mutations showing only a slight change in the $E_a(k_{cat})$.

Figure S5. HDX coverage map of Mt-OMPDC using a set of non-overlapping peptides.

Figure S6. HDX traces comparison at 35°C between substrate-free (WT(apo), L123A(apo)) and 6-azaUMP bound states of wild-type Mt-OMPDC, WT(L), and L123A mutant, L123A(L).

Figure S7. Circular dichroism spectra measuring melting temperature (T_m) for WT(apo), L123A(apo), WT(L), and L123A(L).

Figure S8. D-uptake and change in protection against HDX between WT(L) & L123A(L) at 35°C.

Figure S9. Temperature and time-dependent HDX traces of WT(L) and L123A(L) bound with 6-azaUMP.

Figure S10. Arrhenius-like plot of k_{HDX} versus 1000/T(K^{-1}), illustrating the activation energy for HDX ($E_a(k_{HDX})$) for peptides in WT(L) and L123A(L) of Mt-OMPDC.

Figure S11. Temperature and time-dependent HDX traces of WT(apo) and L123A(apo) to identify regions linked to thermal stability.

Figure S12. Substrate binding induces loop7 closure and interactions with loop5 and phosphate-binding region.

Figure S13. The ILV network identified in Mt-OMPDC connects all three thermal networks.

Figure S14. Intact mass spectra of wild-type and L123A mutants of Mt-OMPDC.

Tables

Table S1. Kinetic parameters of wild-type Mt-OMDPC and mutants.

Table S2. Inhibition constant (K_i) of 6-azaUMP for the OMP decarboxylation reaction.

Table S3. Non-overlapping set of peptides used for HDX-MS analysis.

Table S4. Peptide-specific back-exchange values (%) used for back-exchange correction in HDX.

Table S5A. Change in D-uptake for L123A(apo) vs WT(apo) at 10. The changes are presented in form of normalized percentage change in D-uptake (ΔD(%)) at 35°C.

Table S5B. Change in D-uptake for L123A(apo) vs WT(apo) at 120 min. The changes are presented in form of normalized percentage change in D-uptake (ΔD(%)) at 35°C.

Table S6A. Change in protection against HDX in form of normalized percentage change in D-uptake (ΔD(%)) due to ligand (6-azaUMP) binding to WT and mutant L123Aat 35°C, after 10 minutes of HDX.

Table S6B. Change in protection against HDX in form of normalized percentage change in D-uptake (ΔD(%)) due to ligand (6-azaUMP) binding to WT and mutant L123A at 35°C, after 120 minutes of HDX.

Table S7A. Change in D-uptake for L123A(L) vs WT(L) at 10 min of HDX at 35°C presented in form of normalized percentage change in D-uptake (ΔD(%)).

Table S7B. Change in D-uptake for L123A(L) vs WT(L) at 120 min of HDX at 35°C presented in form of normalized percentage change in D-uptake (ΔD(%)).

Table S7C. Change in D-uptake for L123A(L) vs WT(L) at 10 min of HDX at 50°C presented in form of normalized percentage change in D-uptake (ΔD(%)).

Table S7D. Change in D-uptake for L123A(L) vs WT(L) at 120 min of HDX at 50°C presented in form of normalized percentage change in D-uptake (ΔD(%)).

Table S8. Peptide and temperature-specific initial fitting parameter ($A, B, C, k_1, k_2, k_3, N_T$) along with boundary conditions used for three-exponential fitting of D-uptake vs. ln(*Time*) plot for each peptide in the TD-HDX of WT(L).

Table S9. Peptide and temperature-specific initial fitting parameter ($A, B, C, k_1, k_2, k_3, N_T$) along with boundary conditions used for three-exponential fitting of D-uptake vs. ln(*Time*) plot for each peptide in the TD-HDX of L123A(L).

Table S10-11. Peptide-specific final fitting parameters obtained after three-exponential fitting of D-uptake vs. ln(*Time*) plot for each peptide in WT(L) across various temperatures, shown for replicates 1 and 2, respectively.

Table S12-13. Peptide-specific final fitting parameters obtained after three-exponential fitting of D-uptake vs. ln(*Time*) plot for each peptide in WT(L) across various temperatures, shown for replicates 1 and 2, respectively.
Table S14. Activation energy for HDX ($E_a(k_{HDX})$) for peptides in WT(L) and L123A(L) of Mt-OMPDC.
Table S15. List of primers used.

Materials and Methods

Section-1) Materials

Water was purified using a Milli-Q Plus gradient ultrapure water system (Millipore). Deuterium oxide (99.9% D), orotidine 5-monophosphate (OMP), and pepsin from porcine gastric mucosa were obtained from Sigma-Aldrich. 6-Azauridine-5-monophosphate (6-azaUMP) was sourced from BOC Sciences. The QAI Spin Miniprep kit was purchased from Qiagen. Q5 master mix, DpnI, T4 DNA ligase, T4 polynucleotide kinase, and prestained protein ladder were acquired from New England Biolabs. Mini-PROTEAN® TGXTM gels were obtained from Bio-Rad. *E. coli* strains XL1-Blue and BL21-CodonPlus-RIL were used for plasmid amplification and protein expression, respectively. Competent cells and TEV protease were provided by Macro Lab, University of California, Berkeley.

Section-2) Mt-OMPDC's Expression and Purification

S2a) *Recombinant construction of wild-type and variants of* Mt-*OMPDC:* The OMPDC gene from *Methanothermobacter thermoautotrophicus* strain delta H (gi: 15678157) was cloned into a pET-28a vector with an N-terminal TEV-cleavable linker downstream of a 6x-His tag. The wild-type plasmid served as the template for mutant generation using the NEB site-directed mutagenesis workflow. The primers used for mutant generation are listed in Table S14. All mutants were verified by sequencing at the University of California genomics sequencing facility (Berkeley, CA).

S2b) *Expression and purification of wild-type and variants of* Mt-*OMPDC:* Expression and purification were performed based on a literature protocol,[1] with a specific modification of using the reverse Ni-affinity method instead of size-exclusion chromatography. Wild-type Mt-OMPDC and mutant plasmids were transformed into BL21-CodonPlus-RIL competent cells and grown in Luria-Bertani (LB) medium at 37°C (1.5 L media volume). Once the bacterial culture reached an OD_{600} of 0.6-0.8, protein expression was induced by adding IPTG to a final concentration of 13.3 µM. The cells were then grown at 25°C for 16 hours, followed by harvesting the pellet through centrifugation at 4000 rpm for 45 minutes. The pellet was dissolved in lysis buffer (20 mM Tris, 5 mM imidazole, 0.5 mM TCEP, pH 8.0) for protein purification. The lysate was sonicated for 20 minutes (40% amplitude, 3.2 repetition frequency) and then centrifuged at 13,000 rpm for 45 minutes to pellet the cell debris. The supernatant was passed through a Ni-NTA affinity column at a flow rate of 2 mL/min. Non-specifically bound bacterial proteins were washed away with 50 mL of wash buffer (20 mM Tris, 50 mM imidazole, 0.5 mM TCEP, pH 8.0), and the target protein was eluted using 50 mL of elution buffer (20 mM Tris, 200 mM imidazole, 0.5 mM TCEP, pH 8.0). All elution fractions were pooled and dialyzed overnight in 4L of dialysis buffer (20 mM Tris, 0.5 mM TCEP, pH 7.9). SDS-PAGE analysis of the collected fractions showed the presence of the target protein along with some impurity bands.

The next day, the dialyzed protein was concentrated using a 10-kDa centrifuge filter (Amicon) and further buffer exchanged twice with dialysis buffer to reduce the imidazole concentration. Protein concentration was determined by measuring absorbance at 280 nm (A_{280}) using a Thermo Scientific Nanodrop One. TEV protease (with an N-terminal His-tag) was added to the solution in a 5:1 stoichiometric ratio (protein: TEV protease) and incubated overnight at room temperature with subtle rocking. The solution after TEV-cleavage was then passed through a Ni-NTA affinity column, and the target protein (now lacking the His-tag) was eluted by passing 15 mL of dialysis buffer (without imidazole). The pooled protein fractions were buffer-exchanged using a centrifuge filter into storage buffer (20 mM HEPES, 150 mM NaCl, 1.0 mM TCEP, pH 7.5), and the protein concentration was determined by A280 measurement. The concentration values obtained from the Nanodrop were calibrated using the extinction coefficient calculated online (https://web.expasy.org/protparam/). The protein purity was confirmed by intact mass spectrometry (Fig S14).

Section-3) Enzyme Kinetics, Inhibition and Thermal Stability Assays

S3a) *Kinetics:* Enzyme kinetics for wild-type Mt-OMPDC and mutants were performed using a Cary50Bio ultraviolet-visible spectrophotometer (Varian). The cuvette temperature was controlled by a temperature-sensitive Peltier device, backed by a water bath. The enzymatic reaction rate was measured by utilizing the difference in molecular extinction coefficients between UMP (product) and OMP(substrate) at 279 nm ($\Delta\varepsilon_{UMP\text{-}OMP}$), with values of 2330 $M^{-1}cm^{-1}$ at 15°C, 2380 $M^{-1}cm^{-1}$ at 20°C, 2420 $M^{-1}cm^{-1}$ at 25°C, 2490 $M^{-1}cm^{-1}$ at 35°C, and 2520 $M^{-1}cm^{-1}$ at both 40°C and 45°C.[1] All decarboxylation reactions were conducted in 10 mM MOPS, pH 7.1, using cuvettes with a 1-cm path length. The turnover number (k_{cat}) was determined using 60 µM substrate, with enzyme concentrations varying from 1-20 nM. Accurate determination of K_m (1-2 µM range) using the Michaelis-Menten plot is challenging for OMPDC due to the low molar extinction coefficient of substrate, as shown above. Therefore, K_m was determined by fitting the decarboxylation of [OMP] << K_m, to a first-order equation

$$A_t = A_f + (A_i - A_f)*e^{(-k*t)} \qquad \textbf{(1)}$$

where A_i is the absorbance at a given time, A_f is the final absorbance, A_i is the initial absorbance, t is time, and k is equal to k_{cat}/K_M. Alongside an independent assessment of k_{cat} in saturated substrate conditions, this allows for the extraction of an accurate value for the K_m. The turnover number (k_{cat}) determined using saturated substrate concentrations, was measured across five to seven temperatures in the range of 15 to 45 °C. The data were then fitted using the Arrhenius equation to calculate the activation energy of catalysis ($E_a(k_{cat})$) for the wild-type and mutant forms of Mt-OMPDC.

S3b) *Inhibition constant (K_i) measurement of 6-azaUMP:* The inhibition constant (K_i) for 6-azaUMP in wild-type (WT) and L123A construct of Mt-OMPDPC were estimated at 55°C, the highest temperature used in HDX experiments. K_i was determined by performing OMP decarboxylation in the presence of varying concentrations of 6-azaUMP from 0-1.5 µM. 6-azaUMP acts as a competitive inhibitor, and decrease the rate as described by Equation 2. K_i was calculated using Equation 3, where the slope of the dependence of $K_{m,app}$ on inhibitor concentration is equal to K_m/K_i.

$$V_0 = \frac{Vmax * [S]}{Km + (Km * I/Ki) + [S]} \quad \textbf{(2)}$$

$$K_{m,app} = K_m(1+[I]/K_i) \quad \textbf{(3)}$$

S3c) *Melting temperature (T_m) measurement of wild-type and L123A construct of* Mt-*OMPDC:* The melting temperature (T_m) of WT and L123A mutant of Mt-OMPDC, both in their apo and bound forms, was determined using circular dichroism (CD). For the apo form, 5 µM of the enzyme in 5 mM MOPS buffer was placed in a Starna Cells cuvette (21-Q-10/CD, 1-cm path length) with stirring, and CD was monitored at 225 nm. For the ligand-bound form of WT(L) and L123A(L), the enzyme was mixed with a saturated concentration of 6-azaUMP (a transition state analog; K_i = 0.58 µM).[2] Specifically, 100 µM of 6-azaUMP (99.9% bound form) was added to the 5 µM enzyme solution, and CD was monitored at 225 nm. The same Starna Cells cuvette with stirring was used for all measurements. Data were collected from 10 to 90 °C at 2 °C intervals, with an equilibration time of 5 minutes at each temperature.

Section-4) HDX-MS method and data collection

S4a) *Peptide library generation using tandem mass spectrometry:* Peptide fragments of WT and L123A construct of Mt-OMPDC after pepsin digestion were identified using an LTQ Orbitrap XL mass spectrometer equipped with an electrospray ionization (ESI) source (Thermo Fisher Scientific, Waltham, MA), as described previously.[3] Xcalibur (version 2.0.7, Thermo) and Proteome Discoverer software (version 1.3, SEQUEST, Thermo) were used for data acquisition and peptide identification, respectively.

S4b) *HDX sample preparation:* WT and L123A construct of Mt-OMPDC (with >99% purity) were thawed on ice. For HDX experiments with protein in apo forms (WT(apo) and L123A(apo)), 5-µL aliquots containing 100 µM enzyme were prepared and stored on ice. For HDX experiments in the ligand-bound forms (WT(L) and L123A(L)), the thawed protein was mixed with a saturated

concentration of 6-azaUMP and incubated for 30 minutes to ensure that >99.9% of the enzyme population remained in the ligand-bound [EL] form. The required ligand concentration was calculated based on a dissociation constant of 0.58 μM. In the ligand-bound HDX experiments, the concentrations of enzyme and 6-azaUMP after mixing, but before D_2O addition, were 100 μM and 1 mM, respectively. As with the apo experiments, 5-μL aliquots of the ligand-bound enzyme solution were prepared and stored on ice. The procedure for mixing the protein with D_2O buffer was identical for both apo and bound forms. It involved thermal equilibration of 5 μL aliquot for 30 seconds at the required temperature (water bath), followed by adding 45 μL of pre-equilibrated D_2O buffer to the protein solution to initiate H-D-exchange reaction. The addition of D_2O buffer resulted in a final protein concentration of 10 μM for all experiments. After the HDX reaction, the sample was quenched in an ice-salt bath (-15 °C) for 10 seconds. Then, 20 μL of acid (0.32 M citric acid, pH 2.4) was added and mixed thoroughly by pipetting 20 times to quench the reaction by lowering pH. Next, 20 μL of guanidine chloride (2.0 M citric acid, pH 2.4) was quickly added to initiate protein unfolding, with the solution again pipetted 20 times for thorough mixing. Protein digestion into smaller peptides was then initiated by adding 20 μL of 0.3 mg/mL pepsin, followed by 20 more pipetting cycles. The reaction mixture was kept on ice for 2 minutes to complete digestion, after which 60 μL of the sample was placed into 250-μL polypropylene vial inserts (Agilent) and rapidly frozen in liquid nitrogen. To minimize systematic errors, the same set of pipettes and an identical number of pipetting cycles were used for all samples. The HDX reactions were conducted over multiple time periods (0, 10, 20, 30, 45, 60, 120, 300, 600, 1800, 3600, 5400, 7200, 10,800, and 14,400 seconds), using the same set of mixing times for all samples. Each experiment was run in biological duplicates for all steps, except for H-D exchange which was performed on an ice bath. Temperature-dependent HDX measurements were carried out at seven different temperatures (15, 20, 25, 35, 45, 50, and 55 °C).

S4c) *HDX measurement using liquid chromatography-mass spectrometry (LC-MS):* HDX samples were analyzed using a 1200 series LC system (Agilent Technologies, Santa Clara, CA) that was connected in line with the LTQ Orbitrap XL mass spectrometer (Thermo), as described previously.[3]

S4d) *Back-exchange experiment:* The extent of back-exchange of deuterium to hydrogen during analysis was quantified for all peptides. First, a 50-μL aliquot containing 10 μM enzyme was lyophilized for 48 hours to remove all water. The lyophilized samples were then dissolved in 50 μL of 100% D_2O buffer and incubated at 50 °C (for wild-type Mt-OMPDC) and 40 °C (for L123A mutant of Mt-OMPDC) for 24 hours to facilitate complete exchange of protein hydrogen to deuterium. The 50-μL reaction mixture was then treated with quenching buffer and pepsin, following the same procedure as for normal HDX sample preparation, and flash frozen. Deuterium incorporation was

determined by LC-MS in the same way as for other HDX samples. Back-exchange (BE) was calculated using the formula: BE (%) = 100% - (deuterium incorporation observed as a percentage). The column used for LC-MS analysis of apo forms (WT(apo) and L123A(apo)) was replaced a few months after those experiments. Subsequent LC-MS analysis of the ligand-bound forms (WT(L) and L123A(L)) was performed using a different column. Separate back-exchange measurements were performed for both columns. Table S4 lists the peptide-specific back-exchange (%) for samples analyzed using column 1 (used for LC-MS of WT(apo) and L123A(apo)), and back-exchange (%) for samples analyzed using column 2 (used for LC-MS of WT(L) and L123A(L)). The following formula was applied for back-exchange correction: Corrected value (%) = observed deuterium incorporation percentage / (1 - BE(%)).

S4e) *HDX mass spectrometry raw data analysis:* HDX Workbench[4] was used for analyzing the mass spectrometry data. A curated, non-overlapping set of 19 distinct peptides from both WT and L123A, in apo (WT(apo) and L123A(apo)) and ligand-bound forms (WT(L) and L123A(L)), was selected as the peptide set. Data were analyzed across seven temperatures and two replicates (see Extended Data: HDX Data Table and HDX Summary Table). The curated peptide set generated an HDX coverage map for Mt-OMPDC (both wild-type and mutant L123A), with an average peptide length of 12 residues (±4.4), achieving 94.4% protein sequence coverage and 83.0% amide coverage. Peptides were chosen based on resolution, signal-to-noise ratio, occurrence in all data sets (WT and L123A mutant), and their position within the sequence, with a preference for shorter peptides. Each peptide in every data set was manually evaluated in HDX Workbench at every time point as part of the curation process. This curation ensured no interference from other peptides with similar *m*/*z* and retention times.

Section-5) HDX data analysis at single-temperature and multiple-temperature

S5a) *Single-Temperature HDX:* HDX measurements at 35 °C were analyzed to examine changes in HDX protection resulting from mutation and ligand binding in both wild-type and L123A constructs. HDX protection changes were evaluated by comparing peptide D-uptake in apo forms (WT(apo) and L123A(apo)) with their respective ligand-bound forms (WT(L) and L123A(L)). Data curation was performed using HDX Workbench, followed by the use of a Python script to extract and analyze %D-uptake changes. Furthermore, single-temperature HDX analysis was extended to assess the impact of mutation on the conformational distribution of Mt-OMPDC. This was achieved by comparing the D-uptake of peptides between WT(L) and L123A(L).

S5b) *TDHDX of WT(L) and L123A(L).* The temperature-dependent HDX-MS (TDHDX) method developed in our laboratory is a powerful tool for estimating the activation energy of HDX ($E_a(k_{HDX})$) and its variation with a mutant that impairs activation energy of catalysis ($E_a(k_{cat})$). Thus, TDHDX correlates variation in enthalpy of scaffold's local unfolding with activation enthalpy of catalysis in

active site. The TDHDX method first uses a time-dependent D-uptake measurements for peptides to calculate the rate of HDX (k_{HDX}), and extend the measurement to multiple temperature to estimate activation energy of HDX ($E_a(k_{HDX})$). The k_{HDX} values are determined by fitting the D-uptake data over time for each peptide using a three-exponential fitting model (equation 4). This model captures the complex kinetics of HDX by accounting for distinct exchange regimes (fast, medium, and slow).

$$\mathbf{Daltons} = N_T - Ae^{-k_1 t} - Be^{-k_2 t} - Ce^{-k_3 t} - N_{NE} \quad \mathbf{(4)}$$

The constant k_1, k_2, and k_3 represent the distinct rate regimes of exchange i.e., fast, medium, and slow. A, B, and C correspond to the amplitude of exchange in each regime, indicating their contribution to the overall process. N_T refers to the total number of exchangeable amides, while N_{NE} represents the number of non-exchanging amides. In TDHDX, as temperature increases, the population that shows exchange in slower time-regime (k_3) shifts to a medium time-regime of exchange (k_2), and the amide population exchanging in medium-time regime (k_2) shifts to a fast-time regime (k_1), a pattern consistently observed across all peptides. Therefore, it is crucial to establish accurate boundary conditions for each exchange regime in multi-exponential fitting, for all peptides at each temperature. The boundary conditions for fast (k_1), medium (k_2), and slow (k_3) exchange rates were specifically determined by analyzing the nature of the exponential curve. The procedure began with an initial fitting of all peptides to a three-exponential equation using fixed and standard time-regimes to obtain the initial three-exponential curvature. The boundary conditions for each regime were set as follows: k_1 (100 $min^{-1} \geq k_1 \leq 2.5$ min^{-1}), k_2 (2.5 $min^{-1} \geq k_2 \leq 0.05$ min^{-1}), and k_3 (0.05 $min^{-1} \geq k_3$). Initial guess values for A, B, C, and N_{NE} were set at 25% of N_T (0.25 x N_T).

The resulting D-uptake curved plotted as function of time for each peptide was then manually examined to identify inflection points in the three-exponential curve. The first inflection point in the three-exponential fitting indicates the saturation of the first exponential function (fast exchange- k_1) and the onset of the second exponential process (medium exchange- k_2). Similarly, the second inflection point marks the saturation of HDX in the medium rate regime and the beginning of the slow exchange regime. These inflection time points correspond to the lifetimes of H-D exchange in different regimes. Based on these lifetimes, the upper and lower limits of exchange rates (or boundary conditions) for each regime were determined. As the temperature increases, the inflection points between the fast (k_1) and medium (k_2) exchange regimes shift to earlier time points, resulting in an increase in the lower limit of k_1 and the upper limit of k_2. The observed boundary conditions from these transition regions were then used to determine the initial guess values for A, B, C, k_1, k_2, and k_3. The peptide-specific boundary conditions used for the final fitting in WT(L) and L123A(L) are listed in

Tables S8 and S9, respectively. The final fitting parameters, including k_1, k_2, k_3, A, B, C, and N_{obs} for each peptide at various temperatures in replicates 1 and 2 of WT(L), are detailed in Tables S10 and S11. For L123A(L), the final fitting parameters obtained in replicates 1 and 2 are provided in Tables S12 and S13, respectively.

Finally, the rate of HDX (k_{HDX}) for a peptide at any given temperature was determined using weighted average methods developed previously in our laboratory (Equation 5)

$$\boldsymbol{k_{HDX} = (Bk_2 + Ck_3) / N_T} \quad \textbf{(5)}$$

The constants of Equation 5 are defined in Equation 4. Due to the time constraints of our manual HDX experiments, precise measurement of k_1 is not feasible, so our rate study is limited to the measurable range, which includes k_2 and k_3. We used a weighted average rate constant, k_{HDX} (Equation 5) to capture the overall patterns of amide exchange, as shown in our laboratory's previous work.[5–7] As discussed, with increasing temperature, non-exchanging amides may begin to contribute to k_3, and similarly, slow-exchanging amides may shift to contribute to k_2. To accommodate this transition of amide exchange from one regime to another as a function of temperature, each rate constant is normalized by the number of amides exchanged in that regime (A, B, and C). High-throughput data analysis of this large-scale dataset was conducted using Python code originally developed by Emily J. Thompson (https://github.com/ejt-hdx/2020_HDX_eno). The code was significantly modified to incorporate features for manual data curation, data fitting, and the definition of boundary conditions. The updated workflow has been uploaded to (https://github.com/pankaj-db/HDX-Data-Analysis).

S5c) *T_{melt}-HDX analysis:* Traditionally, HDX experiments combined with denaturant mixing, such as urea or guanidinium-induced unfolding, have been widely used to study spatial and temporal basis of protein stability and unfolding mechanisms. By measuring D-uptake across a range of denaturant concentrations, these experiments identify protein regions with varying protection levels, often highlighting regions of proteins critical for stability and resistance to unfolding. In this study, we employed an alternative approach using temperature-melt HDX (T_{melt}-HDX) to investigate the stability of Mt-OMPDC. By analyzing the thermally stable WT(apo) and the thermally unstable mutant L123A(apo), T_{melt}-HDX enabled us to directly observe how specific protein regions respond to temperature changes, providing detailed insights into areas that are essential for thermal stability.

As shown in Fig. S7, the L123A mutation reduces the melting temperature (T_m) of Mt-OMPDC from 75 to 60 °C. For WT(apo), HDX was performed across a temperature range of 15-55 °C, while for L123A(apo), HDX was limited to 15-50 °C, as the mutant sample precipitated during incubation at 55

°C. The D-uptake as a function of time was plotted using a D-uptake vs. ln(time) plot, as shown in Fig. S11. In the temperature range of our HDX measurements, WT(apo) remains fully in its dimeric form. However, for L123A(apo), with a T_m of 60 °C, a significant portion of the population would dissociate into monomers at higher temperatures. Thus, the k_{HDX} analysis that was used for the ligand-bound form could not be used here for comparison of HDX of the apo form. However, inspection of the D-uptake vs. ln(T) plots showed sharp increases of D-uptake with increasing time and temperature for only three peptides. These three peptides, namely, 71-88, 94-110, and 142-149, are all located at the dimer interface, indicating that the thermal stability of Mt-OMPDC is strongly linked to dimer stability.

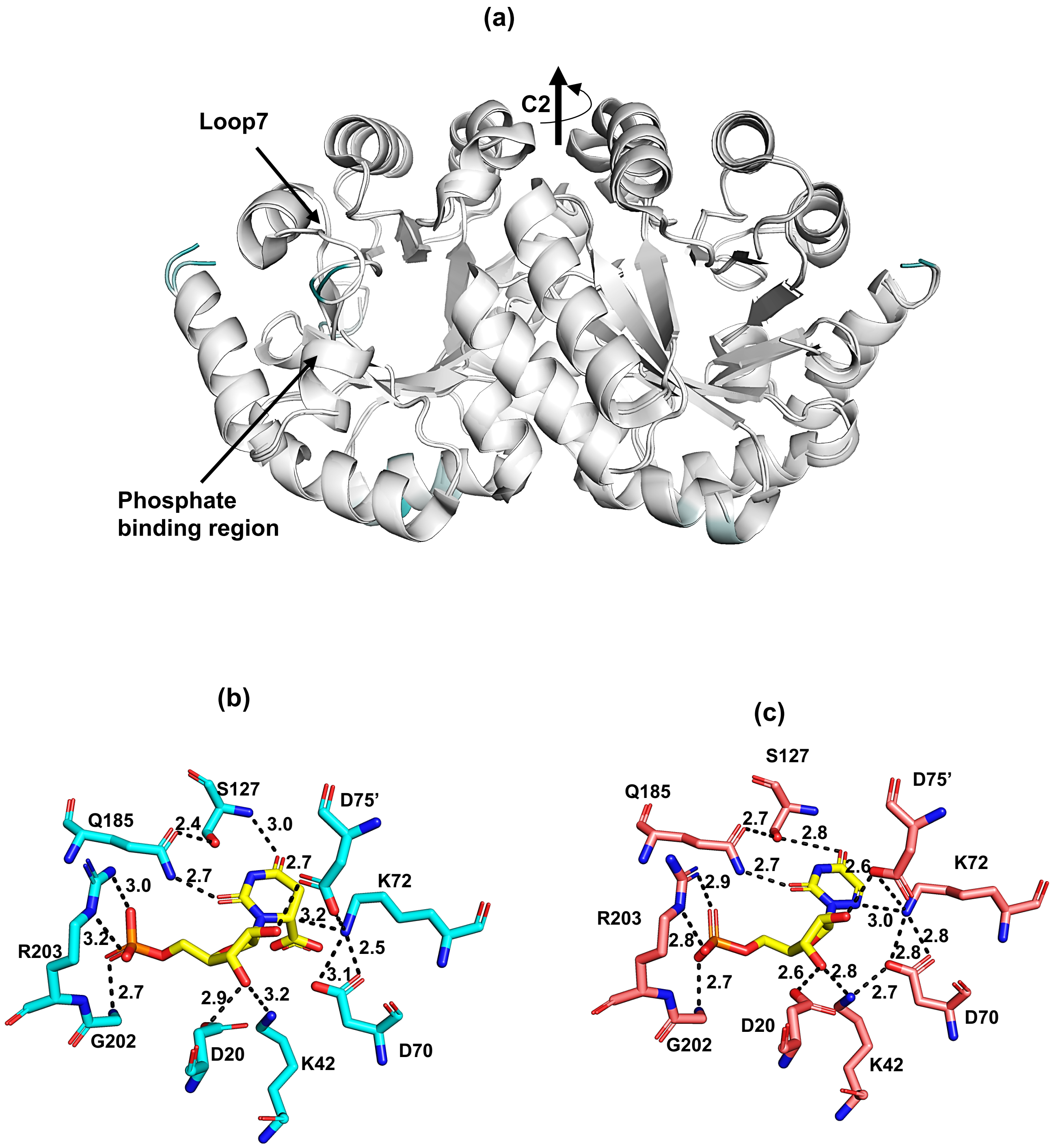

Figure S1. Structural Comparison of Substrate-Analog and Transition-State-Analog Bound Forms of Mt-OMPDC. (a) Crystal structure overlay of Mt-OMPDC in its substrate-analog (SBA)-bound form (PDB: 3G1F) and transition state analog (TSA, 6-AzaUMP)-bound form (PDB: 3G1A) was performed to identify structural changes between the SBA- and TSA-bound states. Residue-specific root mean square deviation (RMSD) values were calculated and visualized on the crystal structure using the PyMOL script ColorbyRMSD.[8] A white-gray-deep teal color gradient was applied, with regions showing minimal structural differences depicted in white and regions with increasing RMSD values progressively colored deep teal, highlighting areas of significant conformational changes. The RMSD of 0.3 Å between the two structures highlights their high degree of similarity. (b) Active site of Mt-OMPDC bound with SBA, displaying distances between key residues. (c) Active site of Mt-OMPDC bound with TSA, with key residue distances indicated. This analysis reveals minimal conformational change (~±0.3 Å) in the active site between SBA and TSA binding.

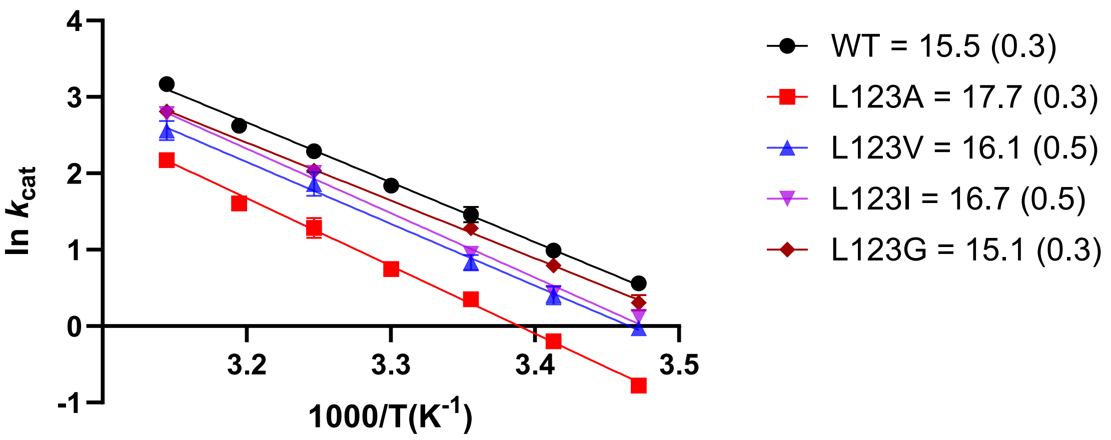

Figure S2. Arrhenius plot of wild-type Mt-OMDPC and L123 mutants. Activation energy ($E_a(k_{cat})$) of WT and mutant are in kcal/mol, with standard deviations in parentheses. Errors represent standard deviations from linear regression fits of biological triplicate measurements.

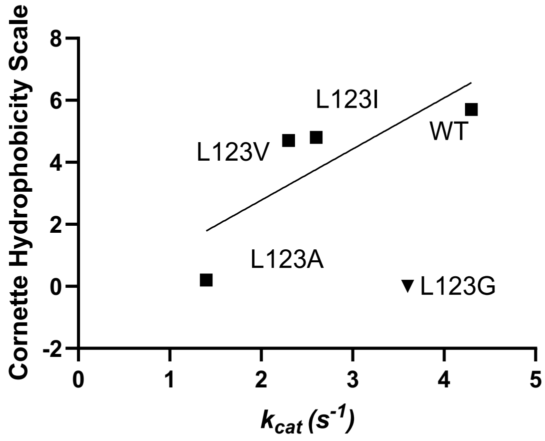

Figure S3. Relationship between the Cornette hydrophobicity scale of the residue at position 123 and catalytic rate (k_{cat}) for reaction (R^2 = 0.65), excluding the L123G mutant, which is represented as an inverse triangle and shown for comparison only.

Table S1. Kinetic parameters of wild-type Mt-OMDPC and its mutants (with standard deviation in parentheses)

	k_{cat} at 25°C (s^{-1})	Activation energy, $E_a(k_{cat})$ (kcal/mol)
WT	4.2 (0.2)	15.3 (0.3)
I68A	0.3 (0.02)	14.7 (0.5)
I68V	3.7	14.9
I68L	1.9(0.2)	15.1(0.9)
I96A	0.05 (0.002)	14.9 (0.7)
I96V	0.9(0.1)	16.1(0.6)
I96L	2.7	14.7
V155A	0.9(0.1)	15.4(0.4)
V155I	2.5	16.3
V155L	1.0 (0.1)	16.6 (0.7)
I178L	2.5(0.2)	15.1(0.4)
I178A	1.2(0.2)	15.3(0.7)
I178V	1.1(0.2)	15.1(0.5)
L123A/V155L	0.3(0.03)	17.9 (0.4)
I96V/L123A	0.4(0.7)	17.3(0.5)
I96V/V155L	1.2 (0.1)	16.3 (0.4)

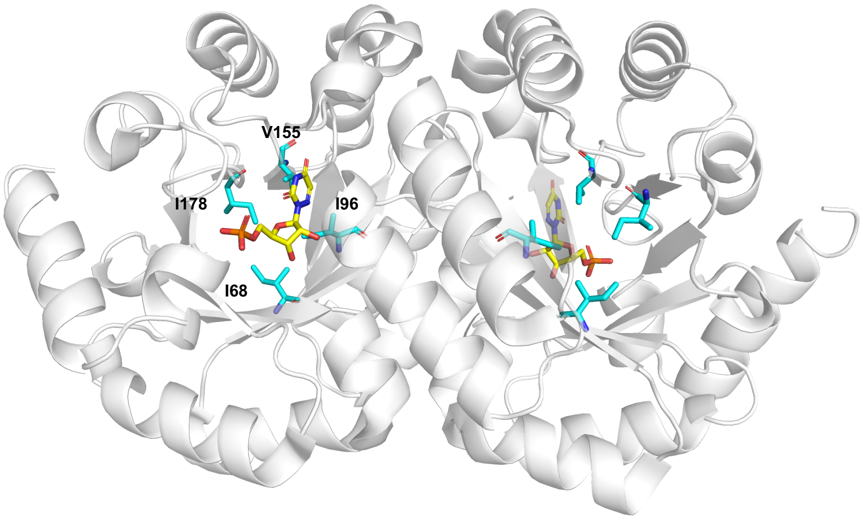

Figure S4. Positions of hydrophobic mutations in Mt-OMPDC, showing only a slight change in the activation energy of catalysis (PDB: 3G1A).

Table S2. The inhibition constant (K_i) of 6-azaUMP for the OMP decarboxylation reaction catalyzed by WT Mt-OMPDC and the L123A mutant. The K_i value at 25°C was obtained from published literature,[2] while the K_i value at 55°C was estimated in this study. Values in parentheses indicate standard deviation obtained from triplicates using linear regression of the inhibition plot. (see Section S5 for details).

	Inhibition constant (K_i) µM	Temperature (°C)
WT	0.58	25
WT	1.0 (0.3)	55
L123A	0.2 (0.2)	55

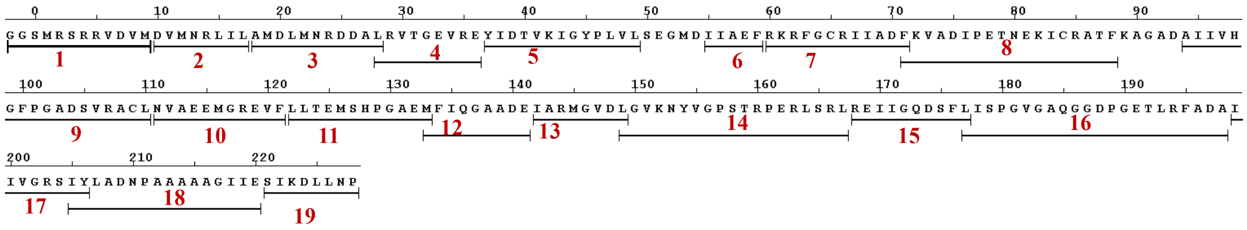

Figure S5. HDX coverage map of Mt-OMPDC using a set of 19 non-overlapping peptides, with an average length of 12 residues (±4.4), achieving 94.4% protein sequence coverage and 83% amide coverage.

Table S3. Non-overlapping peptide set for Mt-OMPDC, including peptide lengths and the number of exchangeable amides, used for HDX-MS analysis.

Peptide number	Start number	End number	Amino acid residues	Exchangeable amides (N_T)	Sequence
1	-2	9	12	10	GGSMRSRRVDVM
2	10	17	8	6	DVMNRLIL
3	18	28	11	9	AMDLMNRDDAL
4	28	36	9	7	LRVTGEVRE
5	37	49	13	10	YIDTVKIGYPLVL
6	55	59	5	3	IIAEF
7	60	71	12	10	RKRFGCRIIADF
8	71	88	18	15	FKVADIPETNEKICRATF
9	94	110	18	15	AIIVHGFPGADSVRACLN
10	111	121	11	9	NVAEEMGREVF
11	122	133	12	9	LATEMSHPGAEM
12	133	141	9	7	MFIQGAADE
13	142	149	8	6	IARMGVDL
14	149	167	19	15	LGVKNYVGPSTRPERLSRL
15	168	177	10	8	REIIGQDSFL
16	177	198	22	18	LISPGVGAQGGDPGETLRFADA
17	199	206	8	6	IIVGRSIY
18	205	220	16	13	IYLADNPAAAAAGIIE
19	221	228	8	6	SIKDLLNP

Table S4. Peptide-specific back-exchange values that are used for back-exchange correction in HDX analysis. LC-MS analysis of WT(apo) and L123A(apo) were done using column 1, while LC-MS of WT(L) and L123A(L) were done using column 2. The averaged back-exchange values for peptides are from three independent experiments and are listed below.

**Peptide**	**Back-Exchange (%) Column 1**	**Back-Exchange (%) Column 2**
-2 -9	48.5	49.7
10-17	57.5	54.6
18-28	54.1	51
28-36	41.6	44.5
37-49	48.8	44.9
55-59	54.6	48.8
60-71	54.4	52.5
71-88	39.2	43.5
94-110	58.3	55.7
111-121	41.7	41
122-133	44.0	44
133-141	37.3	38
142-149	59.0	53.8
149-167	44.4	38.9
168-177	65.8	62.2
177-198	38.2	37.4
199-206	33.0	32.1
205-220	42.2	22.9
221-228	24.5	19.1

Figure S6: Comparison of HDX traces (D-uptake vs. ln(t)) at 35 °C for ligand-free states (WT(apo) and L123A(apo)) and ligand-bound states (WT(L) and L123A(L)) with 6-azaUMP. Each data point represents the average D-uptake from biological replicate experiments. Standard deviations shown for each time point were calculated from these biological replicates.

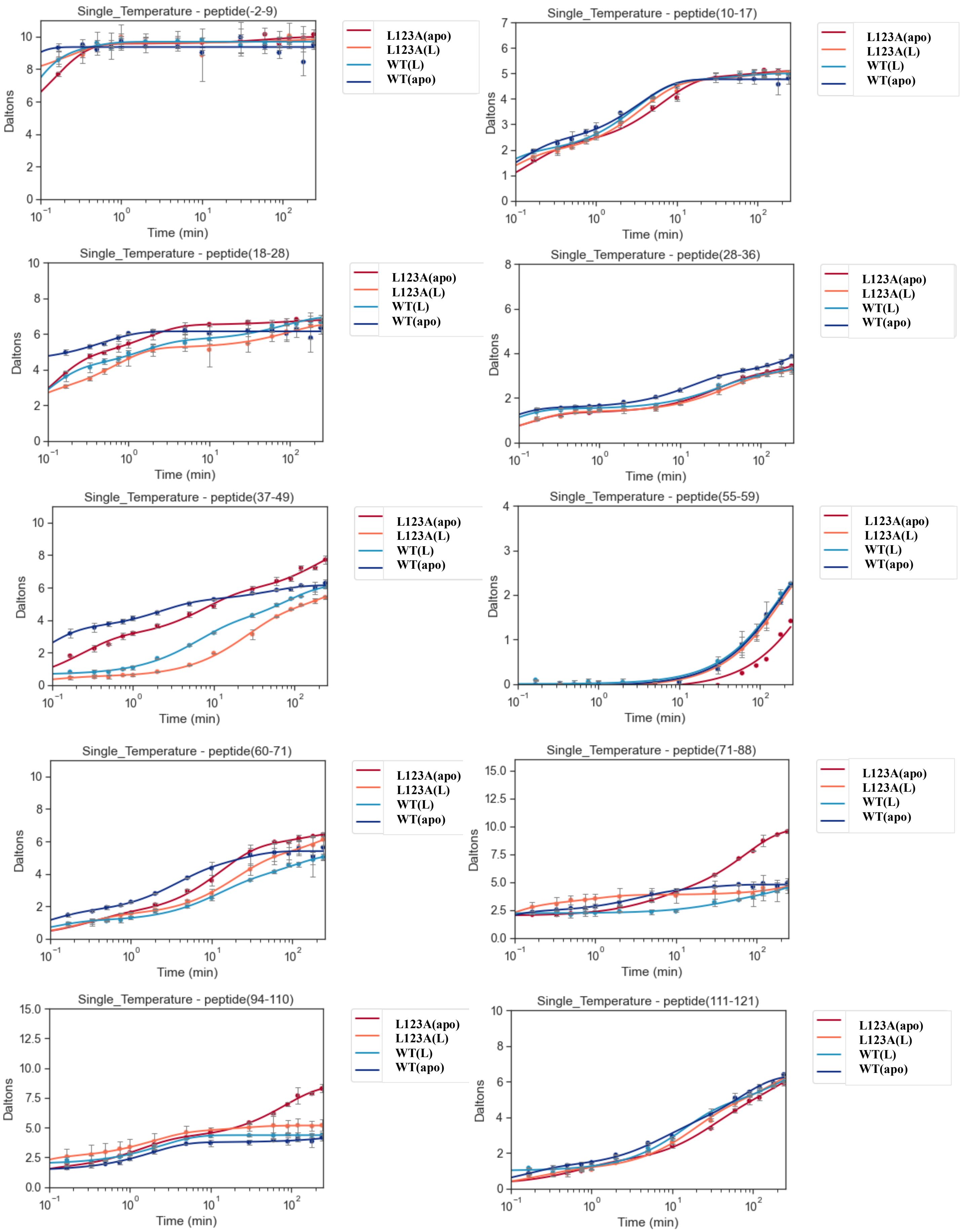

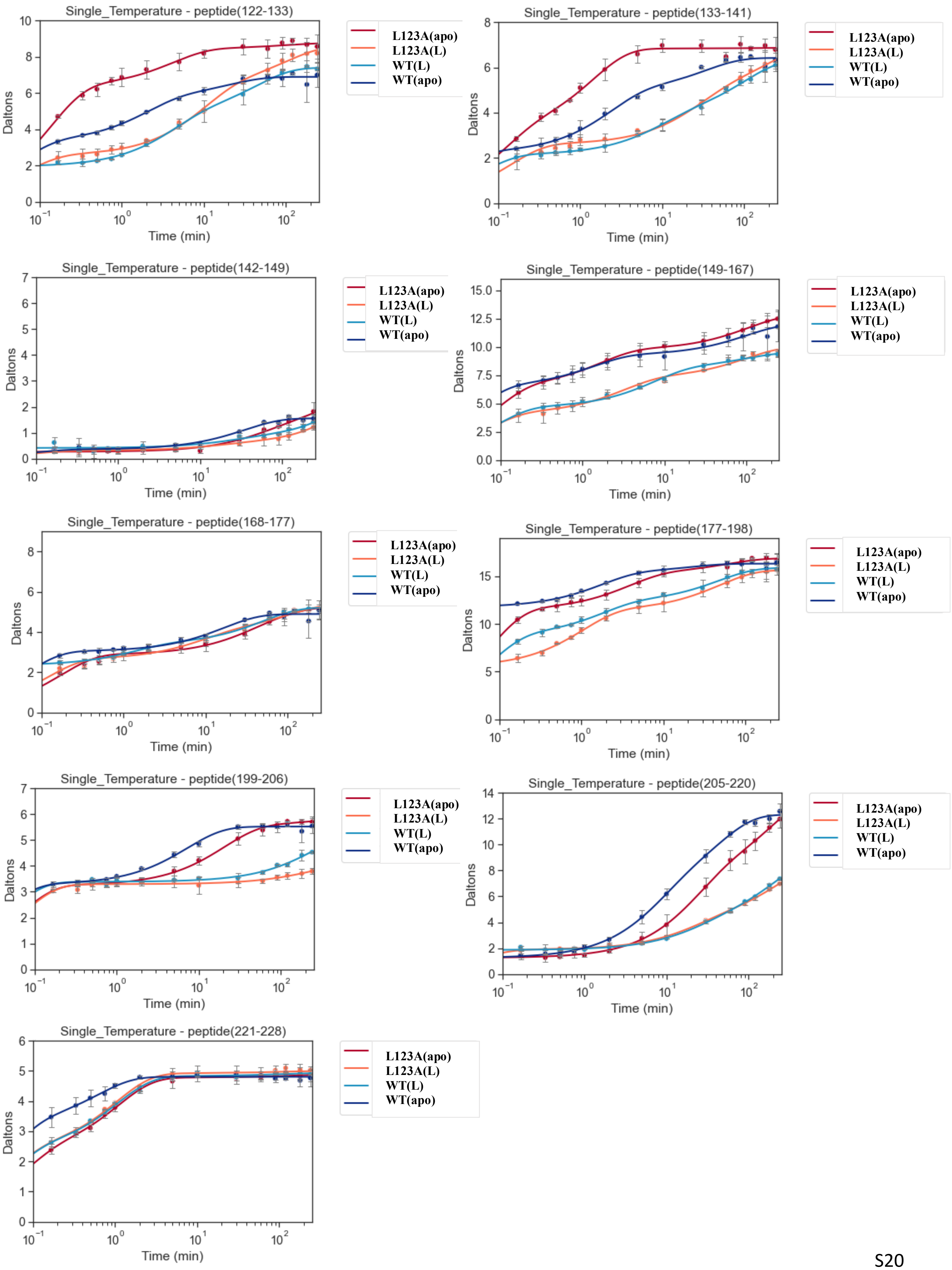
Single_Temperature - peptide(122-133)
Single_Temperature - peptide(133-141)
Single_Temperature - peptide(142-149)
Single_Temperature - peptide(149-167)
Single_Temperature - peptide(168-177)
Single_Temperature - peptide(177-198)
Single_Temperature - peptide(199-206)
Single_Temperature - peptide(205-220)
Single_Temperature - peptide(221-228)
Daltons
Time (min)
L123A(apo)
L123A(L)
WT(L)
WT(apo)

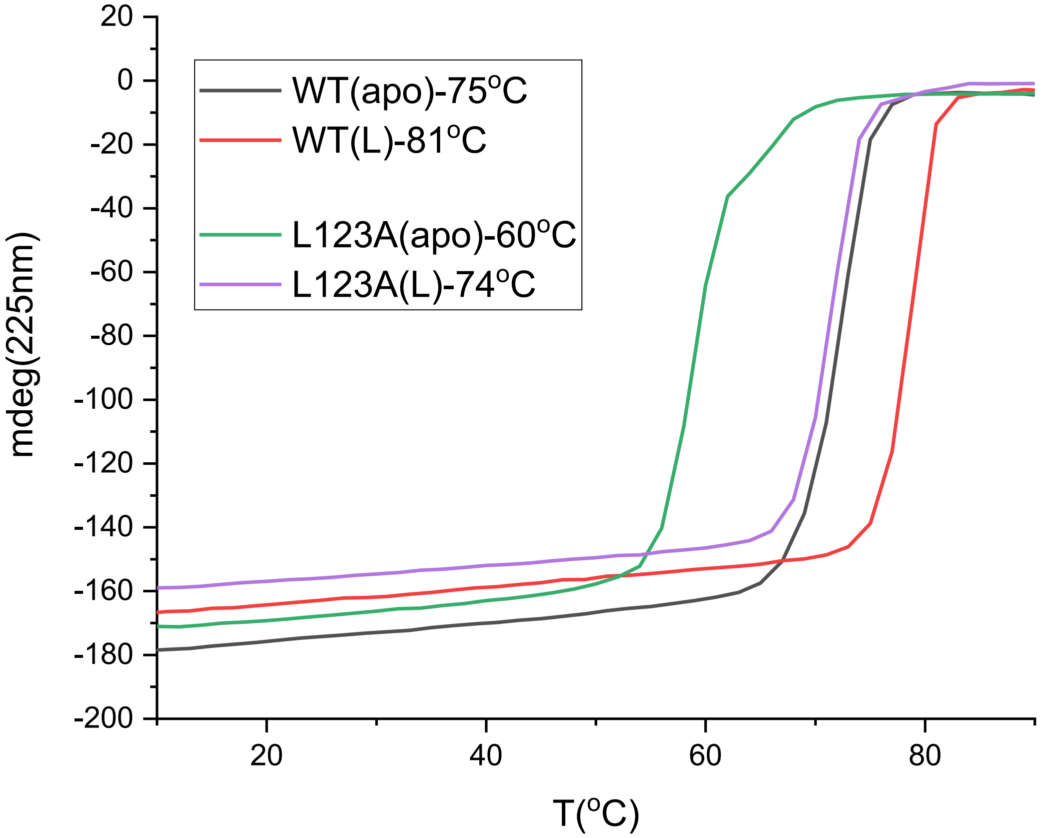

Figure S7. Molar ellipticity for WT(apo), L123A(apo), WT(L) and L123A(L) at θ_{225} as a function of temperature. Background corrected by subtraction of blank buffer melt.

Table S5A. Change in HDX protection due to L123A mutation in Mt-OMPDC, after 10 minutes of HDX at 35°C. The change is presented in form of normalized percentage change in D-uptake (ΔD(%)) for each peptide. To ensure statistical significance, changes exceeding 0.5 Da and 3σ, where σ represents the propagated standard error, were considered. Values for each peptide were averaged from two biological replicate experiments, with statistically significant differences highlighted in bold. "N_T", "SD", and "SPE" represent the total number of exchangeable amides in the peptide, standard deviation, and standard propagated error, respectively.

		**L123A (Apo)**		**WT(Apo)**				
Peptide	N_T	Average Deuteron uptake	SD	Average Deuteron uptake	SD	$D_{L123A(apo)} - D_{WT(apo)}$	SPE	ΔD(%)= $((D_{L123A(apo)} - D_{WT(apo)})/N_T)$ *100
-2 -9	10	9.642	0.293	9.025	0.154	0.617	0.23	6.2
10-17	6	4.054	0.14	4.521	0.092	-0.468	0.12	-7.8
18-28	9	6.546	0.004	6.032	0.383	0.513	0.27	5.7
**28-36**	**7**	**1.746**	**0.07**	**2.349**	**0.081**	**-0.602**	**0.08**	**-8.6**
37-49	10	4.865	0.148	5.28	0.02	-0.416	0.11	-4.2
55-59	3	0.044	0.036	0.044	0.036	0	0.04	0
60-71	10	3.595	0.308	4.35	0.398	-0.756	0.36	-7.6
71-88	15	4.131	0.097	4.215	0.091	-0.084	0.09	-0.6
**94-110**	**14**	**4.624**	**0.2**	**3.709**	**0.296**	**0.915**	**0.25**	**6.5**
**111-121**	**9**	**2.398**	**0.121**	**2.938**	**0.006**	**-0.54**	**0.09**	**-6**
**122-133**	**9**	**8.187**	**0.229**	**6.117**	**0.208**	**2.07**	**0.22**	**23**
**133-141**	**7**	**6.975**	**0.31**	**5.132**	**0.14**	**1.842**	**0.24**	**26.3**
142-149	6	0.3	0.088	0.576	0.005	-0.276	0.06	-4.6
149-167	15	10.076	0.385	9.149	1.159	0.927	0.86	6.2
168-177	8	3.396	0.11	3.749	0.151	-0.354	0.13	-4.4
177-198	18	15.308	0.324	15.653	0.489	-0.345	0.41	-1.9
**199-206**	**6**	**4.216**	**0.16**	**4.852**	**0.097**	**-0.636**	**0.13**	**-10.6**
**205-220**	**13**	**3.802**	**0.794**	**6.184**	**0.42**	**-2.382**	**0.64**	**-18.3**
221-228	5	4.918	0.27	4.878	0.052	0.04	0.19	0.8

Table S5B. Change in HDX protection due to L123A mutation in Mt-OMPDC, after 120 minutes of HDX at 35°C. The change is presented in form of normalized percentage change in D-uptake (ΔD(%)) for each peptide. To ensure statistical significance, changes exceeding 0.5 Da and 3σ, where σ represents the propagated standard error, were considered. Values for each peptide were averaged from two biological replicate experiments, with statistically significant differences highlighted in bold. "N_T", "SD", and "SPE" represent the total number of exchangeable amides in the peptide, standard deviation, and standard propagated error, respectively.

		**L123A (Apo)**		**WT(Apo)**				
Peptide	N_T	Average Deuteron uptake	SD	Average Deuteron uptake	SD	$D_{L123A(apo)} - D_{WT(apo)}$	SPE	ΔD(%)= (($D_{L123A(apo)}$ - $D_{WT(apo)}$)/N_T) *100
-2 -9	10	9.825	0.199	9.736	0.303	0.088	0.26	0.9
10-17	6	5.135	0.017	4.923	0.094	0.211	0.07	3.5
**18-28**	**9**	**6.83**	**0.016**	**6.291**	**0.012**	**0.539**	**0.01**	**6**
28-36	7	3.178	0.041	3.464	0.038	-0.286	0.04	-4.1
**37-49**	**10**	**7.214**	**0.148**	**6.127**	**0.063**	**1.086**	**0.11**	**10.9**
55-59	3	1.562	0.294	1.562	0.294	0	0.29	0
60-71	10	6.217	0.211	5.581	0.448	0.637	0.35	6.4
**71-88**	**15**	**8.724**	**0.535**	**4.899**	**0.017**	**3.825**	**0.38**	**25.5**
**94-110**	**14**	**7.695**	**0.682**	**4.059**	**0.199**	**3.637**	**0.5**	**26**
**111-121**	**9**	**5.103**	**0.168**	**5.704**	**0.156**	**-0.6**	**0.16**	**-6.7**
**122-133**	**9**	**8.887**	**0.15**	**7.103**	**0.006**	**1.784**	**0.11**	**19.8**
133-141	7	6.845	0.095	6.481	0.033	0.364	0.07	5.2
142-149	6	1.433	0.246	1.619	0.008	-0.186	0.17	-3.1
149-167	15	11.876	0.516	11.676	0.546	0.2	0.53	1.3
168-177	8	5.023	0.122	5.053	0.021	-0.031	0.09	-0.4
177-198	18	16.92	0.058	16.599	0.066	0.321	0.06	1.8
199-206	6	5.724	0.036	5.628	0.115	0.097	0.09	1.6
**205-220**	**13**	**10.305**	**0.71**	**11.669**	**0.218**	**-1.364**	**0.53**	**-10.5**
221-228	5	5.098	0.125	4.75	0.037	0.348	0.09	7

Table S6A. Change in HDX protection for wild-type (WT) form of Mt-OMPDC due to binding of 6-azaUMP (TSA), after 10 minutes of HDX at 35°C. The change is presented in form of normalized percentage change in D-uptake (ΔD(%)) for each peptide. To ensure statistical significance, changes exceeding 0.5 Da and 3σ, where σ represents the propagated standard error, were considered. Values for each peptide were averaged from two biological replicate experiments, with statistically significant differences highlighted in bold. "N_T", "SD", and "SPE" represent the total number of exchangeable amides in the peptide, standard deviation, and standard propagated error, respectively.

		**WT (L)**		**WT(apo)**				
Peptide	N_T	Average Deuteron uptake	SD	Average Deuteron uptake	SD	$D_{WT(L)}$-$D_{WT(apo)}$	SPE	ΔD(%)= (($D_{WT(L)}$ - $D_{WT(apo)}$)/N_T) *100
**-2 -9**	**10**	**9.826**	**0.139**	**9.025**	**0.154**	**0.801**	**0.15**	**8**
10-17	6	4.563	0.052	4.521	0.092	0.041	0.07	0.7
18-28	9	5.713	0.243	6.032	0.383	-0.319	0.32	-3.5
**28-36**	**7**	**1.836**	**0.107**	**2.349**	**0.081**	**-0.512**	**0.09**	**-7.3**
**37-49**	**10**	**3.215**	**0.032**	**5.28**	**0.02**	**-2.066**	**0.03**	**-20.7**
55-59	3	0.069	0.076	0.044	0.036	0.025	0.06	0.8
**60-71**	**10**	**2.513**	**0.136**	**4.35**	**0.398**	**-1.837**	**0.3**	**-18.4**
**71-88**	**15**	**2.388**	**0.057**	**4.215**	**0.091**	**-1.827**	**0.08**	**-12.2**
94-110	14	4.312	0.161	3.709	0.296	0.604	0.24	4.3
111-121	9	2.82	0.026	2.938	0.006	-0.118	0.02	-1.3
**122-133**	**9**	**5.067**	**0.035**	**6.117**	**0.208**	**-1.05**	**0.15**	**-11.7**
**133-141**	**7**	**3.473**	**0.283**	**5.132**	**0.14**	**-1.659**	**0.22**	**-23.7**
142-149	6	0.473	0.084	0.576	0.005	-0.104	0.06	-1.7
**149-167**	**15**	**7.151**	**0.235**	**9.149**	**1.159**	**-1.999**	**0.84**	**-13.3**
168-177	8	3.67	0.084	3.749	0.151	-0.079	0.12	-1
**177-198**	**18**	**13.101**	**0.225**	**15.653**	**0.489**	**-2.552**	**0.38**	**-14.2**
**199-206**	**6**	**3.52**	**0.046**	**4.852**	**0.097**	**-1.333**	**0.08**	**-22.2**
**205-220**	**13**	**2.737**	**0.013**	**6.184**	**0.42**	**-3.447**	**0.3**	**-26.5**
221-228	5	4.933	0.079	4.878	0.052	0.056	0.07	1.1

Table S6B. Change in HDX protection for wild-type (WT) form of Mt-OMPDC due to binding of 6-azaUMP (TSA), after 120 minutes of HDX at 35°C. The change is presented in form of normalized percentage change in D-uptake (ΔD(%)) for each peptide. To ensure statistical significance, changes exceeding 0.5 Da and 3σ, where σ represents the propagated standard error, were considered. Values for each peptide were averaged from two biological replicate experiments, with statistically significant differences highlighted in bold. "N_T", "SD", and "SPE" represent the total number of exchangeable amides in the peptide, standard deviation, and standard propagated error, respectively.

		**WT (L)**		**WT(apo)**				
Peptide	N_T	Average Deuteron uptake	SD	Average Deuteron uptake	SD	$D_{WT(L)}$-$D_{WT(apo)}$	SPE	ΔD(%)= (($D_{WT(L)}$ - $D_{WT(apo)}$)/N_T) *100
-2 -9	10	9.631	0.105	9.736	0.303	-0.105	0.23	-1.1
10-17	6	4.876	0.03	4.923	0.094	-0.048	0.07	-0.8
18-28	9	6.553	0.161	6.291	0.012	0.262	0.11	2.9
28-36	7	3.048	0.08	3.464	0.038	-0.416	0.06	-5.9
**37-49**	**10**	**5.47**	**0.04**	**6.127**	**0.063**	**-0.657**	**0.05**	**-6.6**
55-59	3	1.529	0.036	1.562	0.294	-0.033	0.21	-1.1
60-71	10	4.596	0.158	5.581	0.448	-0.984	0.34	-9.8
**71-88**	**15**	**4.035**	**0.243**	**4.899**	**0.017**	**-0.863**	**0.17**	**-5.8**
94-110	14	4.331	0.249	4.059	0.199	0.272	0.23	1.9
111-121	9	5.515	0.137	5.704	0.156	-0.189	0.15	-2.1
122-133	9	7.061	0.056	7.103	0.006	-0.042	0.04	-0.5
**133-141**	**7**	**5.473**	**0.16**	**6.481**	**0.033**	**-1.008**	**0.12**	**-14.4**
142-149	6	1.129	0.182	1.619	0.008	-0.49	0.13	-8.2
**149-167**	**15**	**9.004**	**0.202**	**11.676**	**0.546**	**-2.672**	**0.41**	**-17.8**
168-177	8	5.002	0.011	5.053	0.021	-0.051	0.02	-0.6
**177-198**	**18**	**15.417**	**0.027**	**16.599**	**0.066**	**-1.182**	**0.05**	**-6.6**
**199-206**	**6**	**4.043**	**0.054**	**5.628**	**0.115**	**-1.585**	**0.09**	**-26.4**
**205-220**	**13**	**5.972**	**0.005**	**11.669**	**0.218**	**-5.697**	**0.15**	**-43.8**
221-228	5	4.884	0.048	4.75	0.037	0.134	0.04	2.7

Table S6C. Change in HDX protection for mutant (L123A) form of Mt-OMPDC due to binding of 6-azaUMP (TSA), after 10 minutes of HDX at 35°C. The change is presented in form of normalized percentage change in D-uptake (ΔD(%)) for each peptide. To ensure statistical significance, changes exceeding 0.5 Da and 3σ, where σ represents the propagated standard error, were considered. Values for each peptide were averaged from two biological replicate experiments, with statistically significant differences highlighted in bold. "N_T", "SD", and "SPE" represent the total number of exchangeable amides in the peptide, standard deviation, and standard propagated error, respectively.

		**L123A(L)**		**L123A(apo)**				
Peptide	N_T	Average Deuteron uptake	SD	Average Deuteron uptake	SD	$D_{L123A(L)}$-$D_{L123A(apo)}$	SPE	ΔD(%)= $((D_{L123A(L)} - D_{L123A(apo)})/N_T)$ *100
-2 -9	10	8.874	1.611	9.642	0.293	-0.768	1.16	-7.7
10-17	6	4.306	0.333	4.054	0.14	0.253	0.26	4.2
18-28	9	5.119	0.962	6.546	0.004	-1.427	0.68	-15.9
28-36	7	1.797	0.099	1.746	0.07	0.051	0.09	0.7
**37-49**	**10**	**1.961**	**0.018**	**4.865**	**0.148**	**-2.904**	**0.11**	**-29**
55-59	3	-0.014	0.003	0.044	0.036	-0.059	0.03	-2
60-71	10	2.808	0.285	3.595	0.308	-0.787	0.3	-7.9
71-88	15	3.794	0.674	4.131	0.097	-0.337	0.48	-2.2
94-110	14	4.767	0.274	4.624	0.2	0.144	0.24	1
111-121	9	2.588	0.092	2.398	0.121	0.19	0.11	2.1
**122-133**	**9**	**4.984**	**0.608**	**8.187**	**0.229**	**-3.203**	**0.46**	**-35.6**
**133-141**	**7**	**3.473**	**0.232**	**6.975**	**0.31**	**-3.501**	**0.27**	**-50**
142-149	6	0.477	0.034	0.3	0.088	0.177	0.07	2.9
**149-167**	**15**	**6.969**	**0.066**	**10.076**	**0.385**	**-3.107**	**0.28**	**-20.7**
168-177	8	3.472	0.405	3.396	0.11	0.076	0.3	1
**177-198**	**18**	**12.208**	**0.851**	**15.308**	**0.324**	**-3.1**	**0.64**	**-17.2**
**199-206**	**6**	**3.25**	**0.333**	**4.216**	**0.16**	**-0.967**	**0.26**	**-16.1**
205-220	13	2.859	0.119	3.802	0.794	-0.943	0.57	-7.3
221-228	5	4.918	0.27	4.918	0.27	0	0.27	0

Table S6D. Change in HDX protection for mutant (L123A) form of Mt-OMPDC due to binding of 6-azaUMP (TSA), after 120 minutes of HDX at 35°C. The change is presented in form of normalized percentage change in D-uptake ($\Delta D(\%)$). To ensure statistical significance, changes exceeding 0.5 Da and 3σ, where σ represents the propagated standard error, were considered. Values for each peptide were averaged from two biological replicate experiments, with statistically significant differences highlighted in bold. "N_T", "SD", and "SPE" represent the total number of exchangeable amides in the peptide, standard deviation, and standard propagated error, respectively.

		**L123A(L)**		**L123A(Apo)**				
Peptide	N_T	Average Deuteron uptake	SD	Average Deutero n uptake	SD	$D_{L123A(L)}$-$D_{L123A(apo)}$	SPE	$\Delta D(\%)$= $((D_{L123A(L)} - D_{L123A(apo)})/N_T)$ *100
-2 -9	10	10.062	0.789	9.825	0.199	0.237	0.58	2.4
10-17	6	5.021	0.141	5.135	0.017	-0.114	0.1	-1.9
18-28	9	6.237	0.414	6.83	0.016	-0.593	0.29	-6.6
28-36	7	3.034	0.086	3.178	0.041	-0.144	0.07	-2.1
**37-49**	**10**	**4.937**	**0.066**	**7.214**	**0.148**	**-2.277**	**0.11**	**-22.8**
55-59	3	1.372	0.174	1.562	0.294	-0.189	0.24	-6.3
60-71	10	5.673	0.435	6.217	0.211	-0.545	0.34	-5.4
**71-88**	**15**	**4.493**	**1.159**	**8.724**	**0.535**	**-4.231**	**0.9**	**-28.2**
**94-110**	**14**	**5.204**	**0.584**	**7.695**	**0.682**	**-2.491**	**0.63**	**-17.8**
111-121	9	5.581	0.118	5.103	0.168	0.477	0.15	5.3
122-133	9	8.099	0.357	8.887	0.15	-0.788	0.27	-8.8
**133-141**	**7**	**5.848**	**0.24**	**6.845**	**0.095**	**-0.997**	**0.18**	**-14.2**
142-149	6	0.876	0.15	1.433	0.246	-0.557	0.2	-9.3
**149-167**	**15**	**9.388**	**0.207**	**11.876**	**0.516**	**-2.488**	**0.39**	**-16.6**
168-177	8	5.097	0.053	5.023	0.122	0.074	0.09	0.9
**177-198**	**18**	**15.326**	**0.426**	**16.92**	**0.058**	**-1.594**	**0.3**	**-8.9**
**199-206**	**6**	**3.625**	**0.116**	**5.724**	**0.036**	**-2.099**	**0.09**	**-35**
**205-220**	**13**	**5.888**	**0.156**	**10.305**	**0.71**	**-4.418**	**0.51**	**-34**
221-228	5	5.098	0.125	5.098	0.125	0	0.13	0

Table S7A. Single-temperature HDX analysis was conducted to investigate the impact of mutation on the conformational states of the ligand-bound forms of wild-type (WT(L)) and mutant(L123A(L)) of Mt-OMPDC. The change in D-uptake between the ligand-bound forms of WT and L123A after 10 minutes of HDX at 35°C was quantified as normalized percentage change in D-uptake (ΔD(%)). For statistical significance, only changes greater than 0.5 Da and 3σ, where σ is the standard propagated error, were considered. Values for each peptide were averaged from two biological replicate experiments. Peptides with statistically significant differences are highlighted in bold. "N_T", "SD", and "SPE" denote the total number of exchangeable amides in the peptide, standard deviation, and standard propagated error, respectively.

		**L123A(L)**		**WT(L)**				
Peptide	N_T	Average Deuteron uptake	SD	Average Deuteron uptake	SD	$D_{L123A(L)}$-$D_{WT(L)}$	SPE	ΔD(%)= (($D_{L123A(L)}$ - $D_{WT(L)}$)/N_T) *100
-2 -9	10	8.874	1.611	9.826	0.139	-0.952	1.14	-9.5
10-17	6	4.306	0.333	4.563	0.052	-0.257	0.24	-4.3
18-28	9	5.119	0.962	5.713	0.243	-0.595	0.7	-6.6
28-36	7	1.797	0.099	1.836	0.107	-0.039	0.1	-0.6
**37-49**	**10**	**1.961**	**0.018**	**3.215**	**0.032**	**-1.254**	**0.03**	**-12.5**
55-59	3	-0.014	0.003	0.069	0.076	-0.083	0.05	-2.8
60-71	10	2.808	0.285	2.513	0.136	0.294	0.22	2.9
71-88	15	3.794	0.674	2.388	0.057	1.406	0.48	9.4
94-110	14	4.767	0.274	4.312	0.161	0.455	0.22	3.3
111-121	9	2.588	0.092	2.82	0.026	-0.232	0.07	-2.6
122-133	9	4.984	0.608	5.067	0.035	-0.083	0.43	-0.9
133-141	7	3.473	0.232	3.473	0.283	0	0.26	0
142-149	6	0.477	0.034	0.473	0.084	0.005	0.06	0.1
149-167	15	6.969	0.066	7.151	0.235	-0.182	0.17	-1.2
168-177	8	3.472	0.405	3.67	0.084	-0.199	0.29	-2.5
177-198	18	12.208	0.851	13.101	0.225	-0.893	0.62	-5
199-206	6	3.25	0.333	3.52	0.046	-0.27	0.24	-4.5
205-220	13	2.859	0.119	2.737	0.013	0.122	0.08	0.9
221-228	5	4.918	0.27	4.933	0.079	-0.016	0.2	-0.3

Table S7B. Single-temperature HDX analysis was conducted to investigate the impact of mutation on the conformational states of the ligand-bound forms of wild-type (WT(L)) and mutant(L123A(L)) of Mt-OMPDC. The change in deuterium uptake between the ligand-bound forms of WT and L123A after 120 minutes of HDX at 35°C was quantified as normalized percentage change in D-uptake in ΔD(%). For statistical significance, only changes greater than 0.5 Da and 3σ, where σ is the standard propagated error, were considered. Values for each peptide were averaged from two biological replicate experiments. Peptides with statistically significant differences are highlighted in bold. "N_T", "SD", and "SPE" denote the total number of exchangeable amides in the peptide, standard deviation, and standard propagated error, respectively.

		**L123A(L)**		**WT(L)**				
Peptide	N_T	Average Deuteron uptake	SD	Average Deuteron uptake	SD	$D_{L123A(L)}$-$D_{WT(L)}$	SPE	ΔD(%)= (($D_{L123A(L)}$ - $D_{WT(L)}$)/N_T) *100
-2 -9	10	10.062	0.789	9.631	0.105	0.431	0.56	4.3
10-17	6	5.021	0.141	4.876	0.03	0.146	0.1	2.4
18-28	9	6.237	0.414	6.553	0.161	-0.316	0.31	-3.5
28-36	7	3.034	0.086	3.048	0.08	-0.014	0.08	-0.2
37-49	10	4.937	0.066	5.47	0.04	-0.534	0.05	-5.3
55-59	3	1.372	0.174	1.529	0.036	-0.157	0.13	-5.2
**60-71**	**10**	**5.673**	**0.435**	**4.596**	**0.158**	**1.076**	**0.33**	**10.8**
71-88	15	4.493	1.159	4.035	0.243	0.457	0.84	3
94-110	14	5.204	0.584	4.331	0.249	0.873	0.45	6.2
111-121	9	5.581	0.118	5.515	0.137	0.066	0.13	0.7
**122-133**	**9**	**8.099**	**0.357**	**7.061**	**0.056**	**1.038**	**0.26**	**11.5**
133-141	7	5.848	0.24	5.473	0.16	0.375	0.2	5.4
142-149	6	0.876	0.15	1.129	0.182	-0.253	0.17	-4.2
149-167	15	9.388	0.207	9.004	0.202	0.384	0.2	2.6
168-177	8	5.097	0.053	5.002	0.011	0.094	0.04	1.2
177-198	18	15.326	0.426	15.417	0.027	-0.092	0.3	-0.5
199-206	6	3.625	0.116	4.043	0.054	-0.418	0.09	-7
205-220	13	5.888	0.156	5.972	0.005	-0.084	0.11	-0.6
221-228	5	5.098	0.125	4.884	0.048	0.214	0.09	4.3

Table S7C. Single-temperature HDX analysis was conducted to investigate the impact of mutation on the conformational states of the ligand-bound forms of wild-type (WT(L)) and mutant(L123A(L)) of Mt-OMPDC. The change in deuterium uptake between the ligand-bound forms of WT and L123A after 10 minutes of HDX at 50°C was quantified as normalized percentage change in D-uptake in ΔD(%). For statistical significance, only changes greater than 0.5 Da and 3σ, where σ is the standard propagated error, were considered. Values for each peptide were averaged from two biological replicate experiments. Peptides with statistically significant differences are highlighted in bold. "N_T", "SD", and "SPE" denote the total number of exchangeable amides in the peptide, standard deviation, and standard propagated error, respectively.

		**L123A(L)**		**WT(L)**				
Peptide	N_T	Average Deuteron uptake	SD	Average Deuteron uptake	SD	$D_{L123A(L)}$-$D_{WT(L)}$	SPE	ΔD(%)= (($D_{L123A(L)}$ - $D_{WT(L)}$)/N_T) *100
-2 -9	10	9.203	1.387	9.283	0.242	-0.081	1	-0.8
10-17	6	4.885	0.468	4.846	0.012	0.04	0.33	0.7
18-28	9	5.738	1.004	6.352	0.048	-0.614	0.71	-6.8
28-36	7	2.294	0.04	2.554	0.02	-0.261	0.03	-3.7
**37-49**	**10**	**4.159**	**0.246**	**4.956**	**0.003**	**-0.797**	**0.17**	**-8**
55-59	3	0.537	0.036	0.707	0.128	-0.17	0.09	-5.7
**60-71**	**10**	**4.546**	**0.114**	**3.733**	**0.14**	**0.813**	**0.13**	**8.1**
71-88	15	4.185	0.156	3.552	0.169	0.633	0.16	4.2
94-110	14	5.039	0.186	4.392	0.181	0.646	0.18	4.6
111-121	9	4.112	0.149	4.494	0.116	-0.382	0.13	-4.2
122-133	9	7.047	1.012	6.416	0.114	0.631	0.72	7
133-141	7	4.957	0.618	4.729	0.158	0.228	0.45	3.3
142-149	6	0.556	0.103	0.843	0.104	-0.287	0.1	-4.8
149-167	15	8.395	0.606	8.585	0.058	-0.19	0.43	-1.3
168-177	8	4.132	0.816	4.333	0.155	-0.202	0.59	-2.5
177-198	18	13.971	1.272	14.56	0.358	-0.589	0.93	-3.3
199-206	6	3.612	0.368	4.276	0.019	-0.664	0.26	-11.1
**205-220**	**13**	**4.727**	**0.073**	**5.438**	**0.103**	**-0.712**	**0.09**	**-5.5**
221-228	5	4.926	0.283	4.833	0.158	0.093	0.23	1.9

Table S7D. Single-temperature HDX analysis was conducted to investigate the impact of mutation on the conformational states of the ligand-bound forms of wild-type (WT(L)) and mutant(L123A(L)) of Mt-OMPDC. The change in deuterium uptake between the ligand-bound forms of WT and L123A after 120 minutes of HDX at 50°C was quantified as the normalized percentage change in D-uptake in ΔD(%). For statistical significance, only changes greater than 0.5 Da and 3σ, where σ is the standard propagated error, were considered. Values for each peptide were averaged from two biological replicate experiments. Peptides with statistically significant differences are highlighted in bold. "N_T", "SD", and "SPE" denote the total number of exchangeable amides in the peptide, standard deviation, and standard propagated error, respectively.

		**L123A(L)**		**WT(L)**				
Peptide	N_T	Average Deuteron uptake	SD	Average Deutero n uptake	SD	$D_{L123A(L)}$-$D_{WT(L)}$	SPE	ΔD(%)= (($D_{L123A(L)}$ - $D_{WT(L)}$)/N_T) *100
-2 -9	10	9.918	0.259	9.465	0.144	0.452	0.21	4.5
10-17	6	5.005	0.184	4.876	0.0003	0.129	0.13	2.1
18-28	9	6.839	0.524	6.66	0.256	0.18	0.41	2
28-36	7	3.702	0.228	3.669	0.148	0.033	0.19	0.5
37-49	10	6.237	0.169	6.28	0.113	-0.043	0.14	-0.4
55-59	3	2.702	0.052	2.635	0.049	0.067	0.05	2.2
60-71	10	6.734	0.809	5.603	0.249	1.131	0.6	11.3
**71-88**	**15**	**5.736**	**0.189**	**5.074**	**0.034**	**0.661**	**0.14**	**4.4**
**94-110**	**14**	**5.665**	**0.429**	**4.468**	**0.046**	**1.198**	**0.31**	**8.6**
111-121	9	6.81	0.205	6.628	0.048	0.183	0.15	2
122-133	9	8.923	0.622	7.666	0.193	1.257	0.46	14
133-141	7	6.89	0.491	6.544	0.194	0.346	0.37	4.9
142-149	6	1.518	0.17	1.499	0.069	0.019	0.13	0.3
149-167	15	10.24	0.224	10.036	0.065	0.204	0.17	1.4
168-177	8	5.296	0.372	5.187	0.103	0.109	0.27	1.4
177-198	18	16.431	0.661	16.083	0.45	0.349	0.57	1.9
**199-206**	**6**	**5.104**	**0.239**	**5.637**	**0.078**	**-0.534**	**0.18**	**-8.9**
**205-220**	**13**	**9.668**	**0.446**	**10.741**	**0.278**	**-1.073**	**0.37**	**-8.3**
221-228	5	4.938	0.25	4.86	0.153	0.079	0.21	1.6

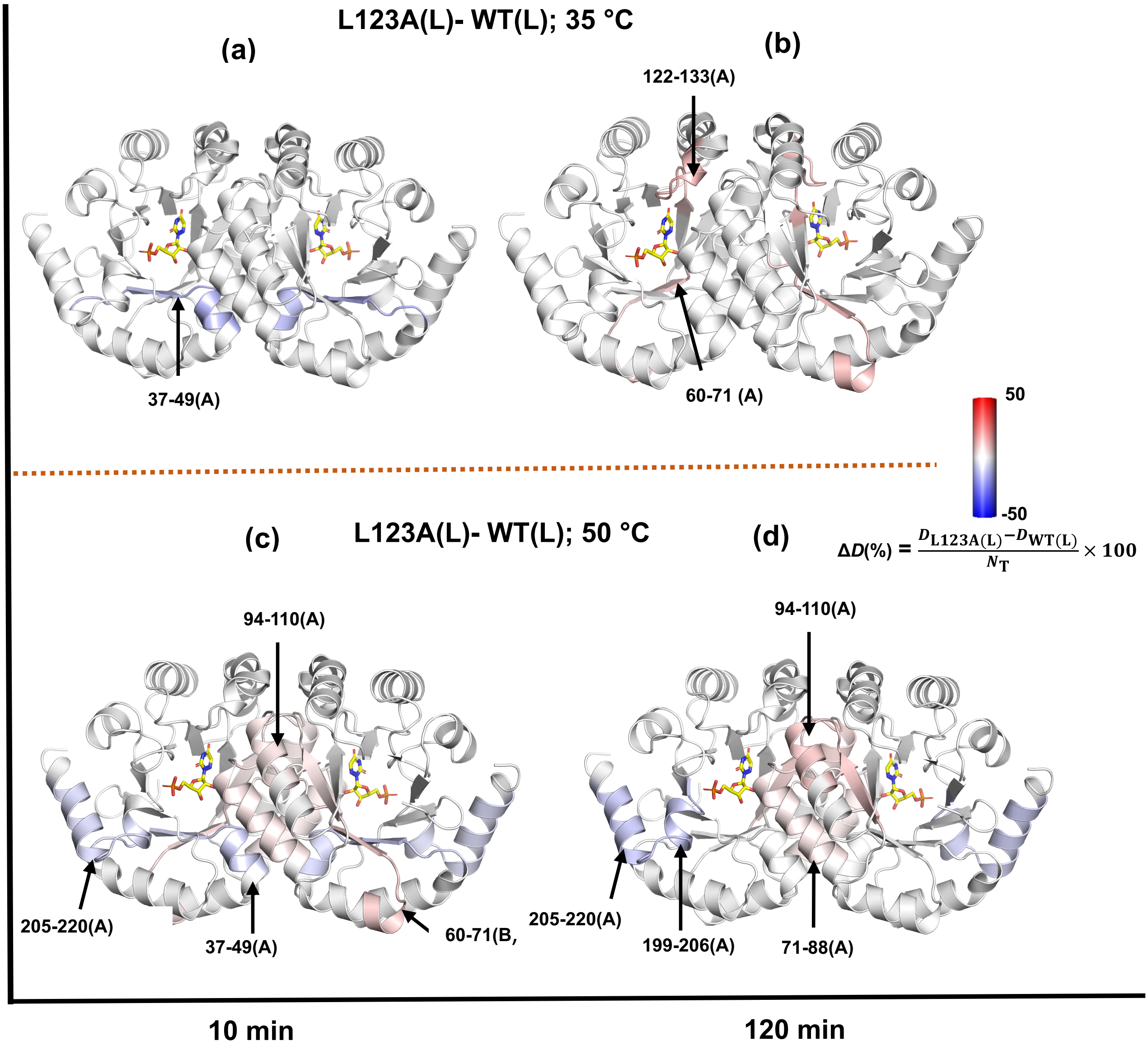

Figure S8. Single-temperature HDX analysis of WT(L) and L123A(L) to examine the impact of mutation the conformational states of the ligand-bound form. Panels (a) and (b) show the normalized percentage change in deuterium uptake (ΔD(%)) between L123A(L) and WT(L) after 10 minutes and 2 hours of HDX at 35°C, respectively. Panels (c) and (d) show the ΔD(%) at 50°C, near the organism's optimal growth temperature (T_{opt} = 65°C), after 10 minutes and 120 minutes, respectively. The ΔD(%) values, reflecting changes in conformational states due to the mutation, were mapped onto the Mt-OMPDC crystal structure (PDB: 3G1A) using a color gradient. Blue indicates regions with lower D-uptake, and red indicates regions with higher D-uptake in L123A(L) compared to WT(L). Significant variations are marked with arrows, and only data with values exceeding 3σ were included.

Tables S8. Initial fitting parameters used for three-exponential fitting of D-uptake vs. ln(*Time*) plots for peptides in the WT(L) construct of Mt-OMPDC. Parameters were derived by manually identifying inflection points in the multi-exponential curve. The lower limit of the fast-exchange regime (k_1) was set as the upper limit for the medium-exchange regime (k_2), and the lower limit of k_2 defined the upper limit for the slow-exchange regime (k_3). Refer to the supporting text for details on parameter derivation.

**Peptide**	**T(°C)**	***A* Initial value (Da)**	***B* Initial value (Da)**	***C* Initial value (Da)**	**k_1 Initial value (min^{-1})**	**k_2 Initial value (min^{-1})**	**k_3 Initial value (min^{-1})**	**N_T Initial value (Da)**	**N_T Lower limit (Da)**	**N_T upper limit (Da)**	**k_1 Lower limit (min^{-1})**	**k_2 Lower limit (min^{-1})**
	15	5	3	2	20	0.25	0.0025	9.5	9	10	10	0.02
	20	5	3	2	20	0.25	0.0025	9.5	9	10	10	0.02
	25	6	2	2	20	0.25	0.0025	9.5	9	10	10	0.02
(-2)-9	35	7	2	1	20	0.25	0.0025	9.5	9	10	10	0.02
	45	7	2	1	20	0.25	0.0025	9.5	9	10	10	0.02
	50	8	2	0.1	20	0.25	0.0025	9.5	9	10	10	0.02
	55	8	2	0.1	20	0.25	0.0025	9.5	9	10	10	0.02
	15	1	2	1.5	20	0.5	0.0025	5	4	6	1	0.01
	20	1	2	1	20	0.5	0.0025	5	4	6	1	0.01
	25	1	2.5	1	20	0.5	0.0025	5	4	6	1	0.01
10-17	35	2	2.5	1	20	0.5	0.0025	5	4	6	1.5	0.02
	45	2	2	2	20	0.5	0.0025	5	4	6	2.5	0.02
	50	2	2	2	20	0.5	0.0025	5	4	6	2.5	0.02
	55	2	2	2	20	0.5	0.0025	5	4	6	2.5	0.02
	15	2	3	3	20	0.25	0.01	5	4	9	0.7	0.02
	20	2	3	3	20	0.25	0.01	5	4	9	0.7	0.02
	25	3	3	3	20	0.25	0.01	5	4	9	0.7	0.02
18-28	35	3	3	3	20	0.25	0.01	5	4	9	0.7	0.02
	45	4	3	3	20	0.25	0.01	5	4	9	0.7	0.02
	50	5	3	3	20	0.25	0.01	5	4	9	0.7	0.02
	55	5	3	3	20	0.25	0.01	5	4	9	0.7	0.02
	15	1.5	2	2	2	0.006	0.0001	3	1	7	0.1	0.005
	20	1.5	2	2	2	0.006	0.0001	3	1	7	0.1	0.005
	25	1.5	2	2	2	0.006	0.0001	3	1	7	0.2	0.005
28-36	35	1.5	2	2	2	0.006	0.0001	3	1	7	0.5	0.005
	45	1.5	2	2	2	0.006	0.0001	3	1	7	0.5	0.005
	50	1.5	2	2	2	0.01	0.0001	3	1	7	1	0.01
	55	1.5	2	2	2	0.01	0.0001	3	1	7	1	0.01
	15	1.5	1.5	1.5	20	0.1	0.0025	5	4	10	0.7	0.005
	20	1.5	1.5	1.5	20	0.1	0.0025	5	4	10	0.7	0.005
	25	2	1.5	1.5	20	0.1	0.0025	5	4	10	1	0.005
	35	2.5	1.5	1.5	20	0.1	0.0025	5	4	10	1	0.005
37-49	45	4	1	1	20	0.1	0.0025	5	4	10	1.5	0.01
	50	4	1	1	20	0.1	0.0025	5	4	10	2.5	0.01
	55	4	1	1	20	0.1	0.0025	5	4	10	2.5	0.01
	15	0	0	1	50	1	0.0025	0.5	0.1	3	2.5	0.5
	20	0	0	1	50	1	0.0025	0.5	0.1	3	2.5	0.5
	25	0	0	1	50	1	0.0025	0.5	0.1	3	2.5	0.5
	35	0	1	1	50	1	0.0025	0.5	0.1	3	2.5	0.5
55-59	45	0	1	2	50	1	0.0025	0.5	0.1	3	2.5	0.5
	50	0	2	1	50	1	0.0025	0.5	0.1	3	2.5	0.5
	55	0	2	1	50	1	0.0025	0.5	0.1	3	2.5	0.5
	15	1.5	0.5	0.5	20	0.05	0.001	2	1	10	0.5	0.005
	20	1.5	2	2	20	0.05	0.001	2	1	10	0.5	0.005
	25	1	1	2	20	0.05	0.001	2	1	10	0.5	0.005
60-71	35	1.5	1.5	1	20	0.5	0.001	2	1	10	1	0.01
	45	2	2	2	20	0.5	0.001	2	1	10	2	0.01
	50	2	2	2	20	0.5	0.001	2	1	10	2	0.01
	55	2	2	2	20	0.5	0.001	2	1	10	2	0.01
	15	3	0.5	1.5	50	0.01	0.001	3	1	15	0.05	0.005
	20	2	0.5	1.5	20	0.01	0.001	3	1	15	0.1	0.005
	25	2	0.5	1.5	20	0.01	0.001	3	1	15	0.1	0.005

71-88	35	2	1	1.5	20	0.01	0.001	3	1	15	0.5	0.005
	45	2	2	2	20	0.5	0.001	3	1	15	2	0.005
	50	2	2	2	20	0.5	0.001	3	1	15	2	0.005
	55	2	2	2	20	0.5	0.001	3	1	15	2	0.005
94-110	15	1	1	1	20	0.5	0.0025	3	2	14	1.5	0.005
	20	1	1	1	20	0.5	0.0025	3	2	14	1.5	0.005
	25	1	1	1	20	0.5	0.0025	3	2	14	1.5	0.005
	35	1	2	1	20	0.5	0.0025	3	2	14	5	0.0075
	45	1	2	2	20	0.5	0.0025	3	2	14	5	0.0075
	50	2	2	3	20	0.5	0.0025	3	2	14	5	0.0075
	55	2	2	2	20	0.5	0.0025	3	2	14	5	0.0075
111-121	15	1	1	1	20	0.5	0.0025	4	2	9	1	0.005
	20	1	1	1	20	0.5	0.0025	4	2	9	1	0.005
	25	1	1	1	20	0.5	0.0025	4	2	9	1	0.005
	35	1	1	2	20	0.5	0.0025	4	2	9	1	0.005
	45	1	2	2	20	0.5	0.0025	4	2	9	2	0.02
	50	1	2	3	20	0.5	0.0025	4	2	9	2	0.02
	55	1	2	3	20	0.5	0.0025	4	2	9	2	0.02
122-133	15	1	1	1	20	0.1	0.01	3	3	9	10	0.05
	20	1	1	1	20	0.1	0.01	3	3	9	10	0.05
	25	1	1	1	20	0.1	0.01	3	3	9	10	0.05
	35	2	1	1	20	0.1	0.01	3	3	9	10	0.05
	45	2	1	1	20	0.1	0.01	3	3	9	10	0.05
	50	2	1	1	20	0.1	0.01	3	3	9	10	0.05
	55	2	1	1	20	0.1	0.01	3	3	9	10	0.05
133-141	15	1	1	2	10	0.05	0.0025	3.5	3	7	0.1	0.005
	20	1	1	3	10	0.05	0.0025	3.5	3	7	0.7	0.005
	25	1	1	3	10	0.05	0.0025	3.5	3	7	1	0.01
	35	2	3	3	10	0.05	0.0025	3.5	3	7	1.5	0.01
	45	0.5	3	0.5	10	0.05	0.0025	3.5	3	7	2	0.01
	50	0.5	3	0.5	10	0.05	0.0025	3.5	3	7	2	0.01
	55	0.5	3	0.5	10	0.05	0.0025	6	3	7	2	0.01
142-149	15	0.2	0.1	0.1	20	0.01	0.0025	0.5	0.1	6	0.1	0.005
	20	0.2	0.5	0.5	20	0.01	0.0025	0.5	0.1	6	0.1	0.005
	25	0.2	0.1	0.5	20	0.01	0.0025	0.5	0.1	6	0.1	0.005
	35	0.2	0.3	0.5	20	0.01	0.0025	0.5	0.1	6	0.1	0.005
	45	0.2	0.5	0.5	20	0.01	0.0025	0.5	0.1	6	0.2	0.01
	50	0.2	0.5	0.5	20	0.01	0.0025	0.5	0.1	6	0.5	0.01
	55	0.2	0.5	0.5	20	0.01	0.0025	0.5	0.1	6	0.5	0.01
149-167	15	3	3	4	10	0.05	0.0025	7.5	7	15	1.5	0.0075
	20	3	3	4	10	0.05	0.0025	7.5	7	15	1.5	0.0075
	25	3	3	4	10	0.05	0.0025	7.5	7	15	1.5	0.0075
	35	4	4	3	10	0.05	0.0025	7.5	7	15	1.5	0.01
	45	6	4	3	10	0.05	0.0025	7.5	7	15	1.5	0.01
	50	6	4	3	10	0.1	0.0025	7.5	7	15	2	0.02
	55	6	4	3	20	0.1	0.0025	7.5	7	15	2	0.02
168-177	15	2	1	1	20	0.1	0.0025	3.5	3	8	1.5	0.02
	20	2	1	1	20	0.1	0.0025	3.5	3	8	1.5	0.02
	25	2	1	1	20	0.1	0.0025	3.5	3	8	1.5	0.02
	35	3	2	1	20	0.1	0.0025	3.5	3	8	1.5	0.02
	45	3	2	1	20	0.5	0.0025	3.5	3	8	1.5	0.05
	50	3	2	1	20	0.5	0.0025	3.5	3	8	1.5	0.07
	55	3	2	1	20	0.5	0.0025	3.5	3	8	1.5	0.07
177-198	15	6	4	3	10	0.1	0.01	13	12	18	1.5	0.05
	20	6	4	3	10	0.1	0.01	13	12	18	1.5	0.05
	25	6	4	3	10	0.1	0.01	13	12	18	1.5	0.05
	35	8	4	3	10	0.1	0.01	13	12	18	1.5	0.05
	45	10	5	2	10	0.1	0.005	13	12	18	1.5	0.07
	50	10	5	1	10	0.1	0.005	13	12	18	1.5	0.07
	55	10	5	1	10	0.1	0.005	13	12	18	1.5	0.07
	15	3	0.5	2	20	0.01	0.0001	3.5	3	6	0.05	0.005
	20	3	0.5	2	20	0.01	0.0025	3.5	3	6	0.05	0.005
	25	3	0.5	2	20	0.01	0.0025	3.5	3	6	0.05	0.005

199-206	35	3	1.5	0.5	20	0.01	0.0025	3.5	3	6	0.05	0.005
	45	3	2	0.5	20	0.01	0.0025	3.5	3	6	0.1	0.005
	50	3	2	0.5	20	0.01	0.0025	3.5	3	6	0.5	0.005
	55	3	2	0.5	20	0.01	0.0025	3.5	3	6	0.5	0.005
205-220	15	1.5	0.5	4	20	0.5	0.0025	12	11.5	14	10	0.05
	20	1.5	0.5	5	20	0.5	0.0025	12	11.5	14	10	0.05
	25	1.5	1	6	20	0.5	0.0025	12	11.5	14	10	0.05
	35	1.5	2.5	7	20	0.5	0.0025	12	11.5	14	10	0.05
	45	1.5	4	7	20	0.5	0.0025	12	11.5	14	10	0.05
	50	2	5	5	20	0.5	0.0025	12	11.5	14	10	0.05
	55	2	5	5	20	0.5	0.0025	12	11.5	14	10	0.05
221-228	15	2	2.5	0.5	20	0.5	0.0025	4.9	4.9	6	1	0.01
	20	2	2.5	0.5	20	0.5	0.0025	4.9	4.9	6	1	0.01
	25	2.5	2	0.5	20	0.5	0.0025	4.9	4.9	6	1	0.01
	35	3	2	0.1	20	0.5	0.0025	4.9	4.9	6	1.5	0.01
	45	4	1	0.1	20	0.1	0.0025	4.9	4.9	6	1.5	0.01
	50	4.5	0.5	0	20	0.1	0.0025	5	4.99	6	4	0.01
	55	4.5	0.5	0	20	0.1	0.0025	5	4.99	6	4	0.01

Tables S9. Initial fitting parameters used for three-exponential fitting of D-uptake vs. ln(*Time*) plots for peptides in the L123A(L) construct of Mt-OMPDC. Parameters were derived by manually identifying inflection points in the multi-exponential curve. The lower limit of the fast-exchange regime (k_1) was set as the upper limit for the medium-exchange regime (k_2), and the lower limit of k_2 defined the upper limit for the slow-exchange regime (k_3). Refer to the supporting text for details on parameter derivation.

Peptide	T(°C)	*A* Initial value (Da)	*B* Initial value (Da)	*C* Initial value (Da)	k_1 Initial value (min^{-1})	k_2 Initial value (min^{-1})	k_3 Initial value (min^{-1})	N_T Initial value (Da)	N_T Lower limit (Da)	N_T upper limit (Da)	k_1 Lower limit (min^{-1})	k_2 Lower limit (min^{-1})
(-2)-9	15	7	1	0.1	20	0.25	0.0025	9.5	9	10	10	0.01
	20	5	1	0.1	20	0.25	0.0025	9.5	9	10	10	0.01
	25	6	1	0.1	20	0.25	0.0025	9.5	9	10	10	0.01
	35	7	1	0.1	20	0.25	0.0025	9.5	9	10	10	0.01
	45	7	1	0.1	20	0.25	0.0025	9.5	9	10	10	0.01
	50	8	1	0.1	20	0.25	0.0025	9.5	9	10	10	0.01
	55	8	1	0.1	20	0.25	0.0025	9.5	9	10	10	0.01
10-17	15	1	4	0.1	20	0.5	0.002	5	4	6	1	0.005
	20	1	4	0.1	20	0.5	0.002	5	4	6	1	0.005
	25	1	4	0.1	20	0.5	0.002	5	4	6	1	0.005
	35	2	4	0.1	20	0.5	0.002	5	4	6	1.5	0.01
	45	2	3	0.1	20	0.5	0.002	5	4	6	5	0.05
	50	2	3	0.1	20	0.5	0.002	5	4	6	5	0.05
	55	3	3	0.1	20	0.5	0.002	5	4	6	5	0.05
18-28	15	2	3	1	20	0.5	0.001	5	4	9	1	0.005
	20	2	3	1	20	0.5	0.01	5	4	9	1	0.01
	25	3	3	1	20	0.5	0.01	5	4	9	1	0.01
	35	3	3	1	20	0.5	0.01	5	4	9	1.5	0.01
	45	3	3	1	20	0.5	0.01	5	4	9	2	0.05
	50	4	3	1	20	0.5	0.01	5	4	9	2.5	0.05
	55	4	3	1	20	0.5	0.01	5	4	9	2.5	0.05
28-36	15	1	1	0.1	2	0.01	0.001	2	1	7	0.1	0.005
	20	1	1	0.1	2	0.01	0.001	2	1	7	0.1	0.005
	25	1	1	0.1	2	0.01	0.001	2	1	7	0.1	0.005
	35	1	2	0.5	2	0.01	0.001	2	1	7	0.5	0.0075
	45	1	2	0.5	2	0.01	0.001	2	1	7	0.5	0.01
	50	1	2	1	2	0.01	0.001	2	1	7	1	0.01
	55	1	2	1	2	0.01	0.001	2	1	7	1	0.01
37-49	15	0.3	2	0.5	10	0.5	0.0025	3	2	10	0.7	0.003
	20	0.3	2	0.5	10	0.5	0.0025	3	2	10	0.7	0.005
	25	0.3	2	0.5	10	0.5	0.0025	3	2	10	0.7	0.005
	35	0.3	3	1	10	0.5	0.0025	3	3	10	0.7	0.005
	45	0.3	3	1	10	0.5	0.0025	6	4	10	1	0.075
	50	0.3	3	1	10	0.5	0.0025	6	5	10	2.5	0.01
	55	0.3	3	1	10	0.5	0.0025	6	5	10	2.5	0.01
55-59	15	0	0	1	50	1	0.0025	0.5	0	3	5	0.05
	20	0	0	1	50	1	0.0025	0.5	0	3	5	0.05
	25	0	0	1	50	1	0.0025	0.5	0	3	5	0.05
	35	0	1	1	50	1	0.0025	2	1	3	5	0.1
	45	0	1	2	50	1	0.0025	2.5	2	3	5	0.1
	50	0	2	1	50	1	0.0025	2.5	2	3	5	0.1
	55	0	2	1	50	1	0.0025	2.5	2	3	5	0.1
60-71	15	0.5	3	0.5	5	0.05	0.001	3	2	10	0.1	0.005
	20	0.5	3	0.5	5	0.05	0.001	3	2	10	0.1	0.005
	25	0.5	3	1	5	0.05	0.001	3	2	10	0.1	0.005
	35	1	4	1	5	0.5	0.001	3	2	10	0.5	0.0075
	45	1	4	2	5	0.5	0.001	3	2	10	0.5	0.01
	50	1.5	4	2	5	0.5	0.001	3	2	10	1	0.025
	55	1.5	4	2	5	0.5	0.001	3	2	10	1	0.025
	15	3	0.5	1.5	20	0.05	0.001	5	3	15	0.05	0.005
	20	2	0.5	1.5	20	0.05	0.001	5	3	15	0.05	0.005

71-88	25	2	0.5	1.5	20	0.05	0.001	5	3	15	0.05	0.005
	35	2	1	1.5	20	0.05	0.001	5	3	15	1.5	0.05
	45	2	2	2	20	0.05	0.001	5	3	15	1.5	0.05
	50	2	2	2	20	0.05	0.001	5	3	15	1.5	0.05
	55	2	2	2	20	0.05	0.001	5	3	15	1.5	0.05
94-110	15	1	1	1	20	0.5	0.0025	7	5	14	1	0.005
	20	1	1	1	20	0.5	0.0025	7	5	14	1	0.005
	25	1	1	1	20	0.5	0.0025	7	5	14	1	0.005
	35	1	2	1	20	0.5	0.0025	7	5	14	2	0.01
	45	1	2	2	20	0.5	0.0025	7	5	14	2	0.01
	50	2	2	3	20	0.5	0.0025	7	5	14	5	0.01
	55	2	2	2	20	0.5	0.0025	7	5	14	5	0.01
111-121	15	1	1	1	20	0.5	0.0025	3	2	9	1	0.005
	20	1	1	1	20	0.5	0.0025	3	2	9	1	0.005
	25	1	1	1	20	0.5	0.0025	3	2	9	1	0.005
	35	1	1	2	20	0.5	0.0025	3	2	9	1.5	0.005
	45	1	2	2	20	0.5	0.0025	3	2	9	2	0.01
	50	1	2	3	20	0.5	0.0025	3	2	9	2.5	0.01
	55	1	2	3	20	0.5	0.0025	3	2	9	2.5	0.01
122-133	15	1	1	1	20	0.1	0.001	8	6	9	1	0.005
	20	1	1	1	20	0.1	0.001	8	6	9	1	0.005
	25	1	1	1	20	0.1	0.001	8	6	9	1	0.005
	35	2	1	1	20	0.1	0.001	8	6	9	2	0.01
	45	2	1	1	20	0.1	0.001	8	6	9	2.5	0.01
	50	2	1	1	20	0.1	0.001	8	6	9	2.5	0.02
	55	2	1	1	20	0.1	0.001	8	6	9	10	0.05
133-141	15	1	1	2	10	0.05	0.0025	6	4	7	0.075	0.005
	20	1	1	3	10	0.05	0.0025	6	4	7	0.075	0.005
	25	1	1	3	10	0.05	0.0025	6	4	7	0.1	0.005
	35	2	3	3	10	0.05	0.0025	6	4	7	1	0.005
	45	0.5	3	0.5	10	0.05	0.0025	6	4	7	1	0.01
	50	0.5	3	0.5	10	0.05	0.0025	6	4	7	2	0.01
	55	0.5	3	0.5	10	0.05	0.0025	6	4	7	2	0.01
142-149	15	0.2	0.5	0.5	20	0.01	0.0025	1	0.1	6	0.1	0.005
	20	0.2	0.5	0.5	20	0.01	0.0025	1	0.1	6	0.1	0.005
	25	0.2	0.1	0.5	20	0.01	0.0025	1	0.1	6	0.1	0.005
	35	0.2	0.3	0.5	20	0.01	0.0025	1	0.1	6	0.1	0.005
	45	0.2	0.5	0.5	20	0.01	0.0025	1	0.1	6	0.2	0.005
	50	0.2	0.5	0.5	20	0.01	0.0025	1	0.1	6	0.5	0.005
	55	0.2	0.5	0.5	20	0.01	0.0025	1	0.1	6	0.5	0.005
149-167	15	3	2.5	0.5	10	0.05	0.0025	7	5	15	0.2	0.005
	20	3	2.5	0.5	10	0.05	0.0025	7	5	15	0.5	0.005
	25	3	2.5	0.5	10	0.05	0.0025	7	5	15	0.5	0.005
	35	4	2.5	0.5	10	0.05	0.0025	7	5	15	1	0.01
	45	4	2.5	0.5	10	0.05	0.0025	7	5	15	1.5	0.01
	50	4	2.5	0.5	10	0.1	0.0025	7	5	15	2	0.025
	55	4	2.5	0.5	20	0.1	0.0025	7	5	15	2	0.05
168-177	15	2	1	1	20	0.05	0.0025	5	3	8	0.1	0.005
	20	2	1	1	20	0.05	0.0025	5	3	8	0.1	0.005
	25	2	1	1	20	0.05	0.0025	5	3	8	1	0.01
	35	3	2	1	20	0.05	0.0025	5	3	8	1	0.01
	45	3	2	1	20	0.05	0.0025	5	3	8	2.5	0.01
	50	3	2	1	20	0.1	0.0025	5	3	8	2.5	0.025
	55	3	2	1	20	0.1	0.0025	5	3	8	2.5	0.025
177-198	15	6	4	3	10	0.1	0.01	13	11	18	1.5	0.05
	20	6	4	3	10	0.1	0.01	13	11	18	1.5	0.05
	25	6	4	3	10	0.1	0.01	13	11	18	1.5	0.05
	35	8	4	3	10	0.1	0.01	13	11	18	1.5	0.05
	45	10	5	2	10	0.1	0.005	13	11	18	1.5	0.07
	50	10	5	1	10	0.1	0.005	13	11	18	1.5	0.07
	55	10	5	1	10	0.1	0.005	13	11	18	1.5	0.07
	15	1	2	0.2	20	0.05	0.0001	2.5	2	6	0.05	0.005
	20	1	2	0.4	20	0.05	0.0001	2.5	2	6	0.05	0.005

199-206	25	1	2	0.4	20	0.05	0.0025	2.5	2	6	0.05	0.005
	35	1	2	0.5	20	0.05	0.0025	2.5	2	6	0.05	0.005
	45	1	2	1	20	0.05	0.0025	2.5	2	6	0.1	0.005
	50	1	2	2	20	0.05	0.0025	2.5	2	6	0.5	0.005
	55	1	2	2	20	0.05	0.0025	2.5	2	6	1	0.005
205-220	15	2	1	1	20	0.5	0.0025	8	3	14	1	0.005
	20	2	1	1	20	0.5	0.0025	8	3	14	1	0.005
	25	2	1	1	20	0.5	0.0025	8	3	14	1	0.005
	35	2	2	1	20	0.5	0.0025	8	3	14	2	0.005
	45	2	4	1	20	0.5	0.0025	8	3	14	2	0.01
	50	2	5	1	20	0.5	0.0025	8	3	14	2	0.01
	55	2	5	1	20	0.5	0.0025	8	3	14	2	0.01
221-228	15	2	2.5	0.5	20	0.5	0.0025	4.9	4.9	5	1.5	0.01
	20	2	2.5	0.5	20	0.5	0.0025	4.9	4.9	5	1.5	0.01
	25	2.5	2	0.5	20	0.5	0.0025	4.9	4.9	5	1.5	0.01
	35	3	2	0.1	20	0.5	0.0025	4.9	4.9	5	1.5	0.01
	45	4	1	0.1	20	0.1	0.0025	4.9	4.9	5	3	0.01
	50	4.5	0.5	0	20	0.1	0.0025	5	4.99	5	3	0.01
	55	4.5	0.5	0	20	0.1	0.0025	5	4.99	5	3	0.01

Figure S9. Temperature- and time-dependent HDX traces for the 6-azaUMP bound forms of Mt-OMDPC, with WT(L) (right) and L123A(L) (left) were analyzed. HDX traces for all 19 non-overlapping peptides were collected at 14 time points ranging from 10 seconds to 4 hours across 15–55°C, using two independent biological replicates.

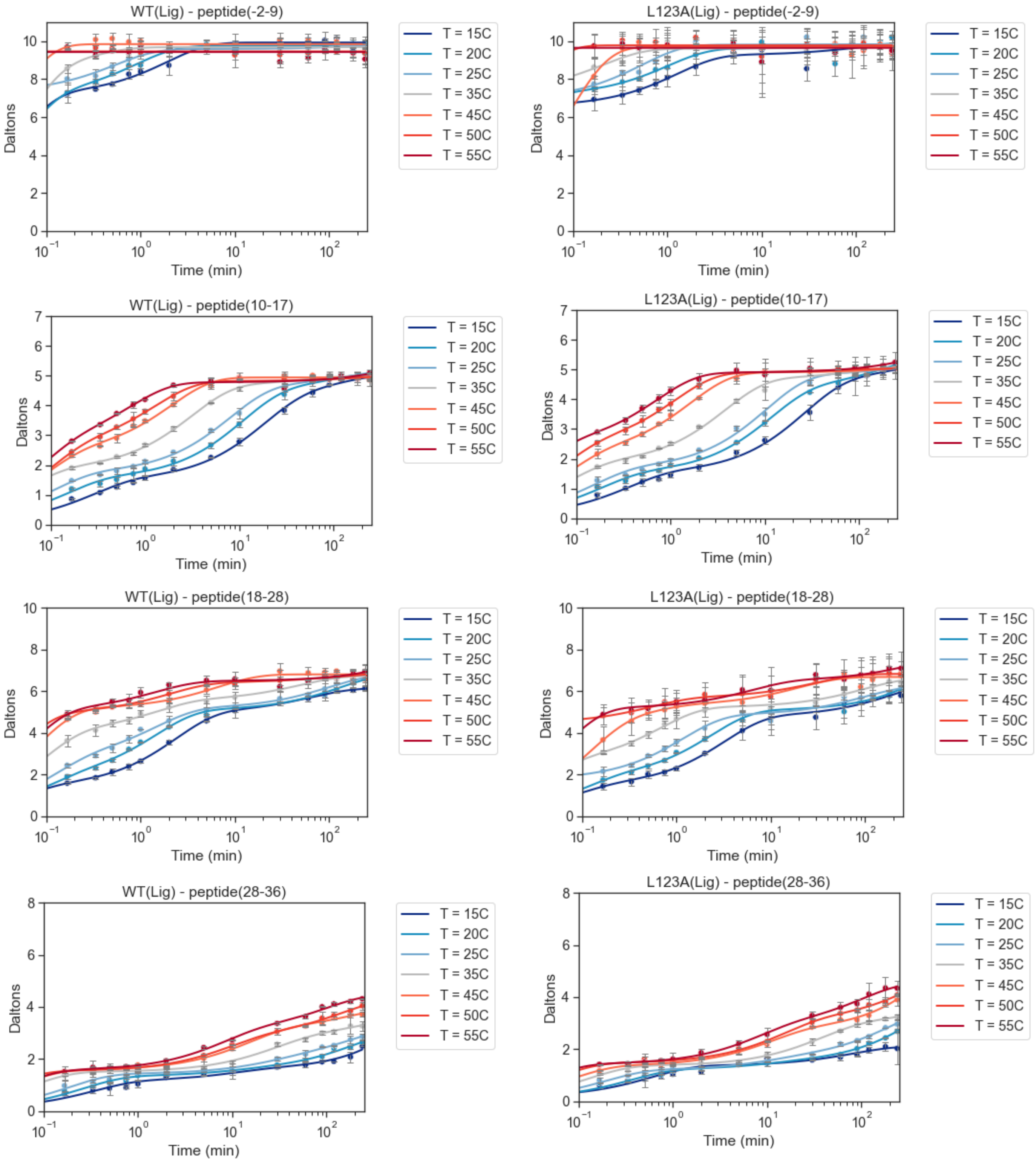

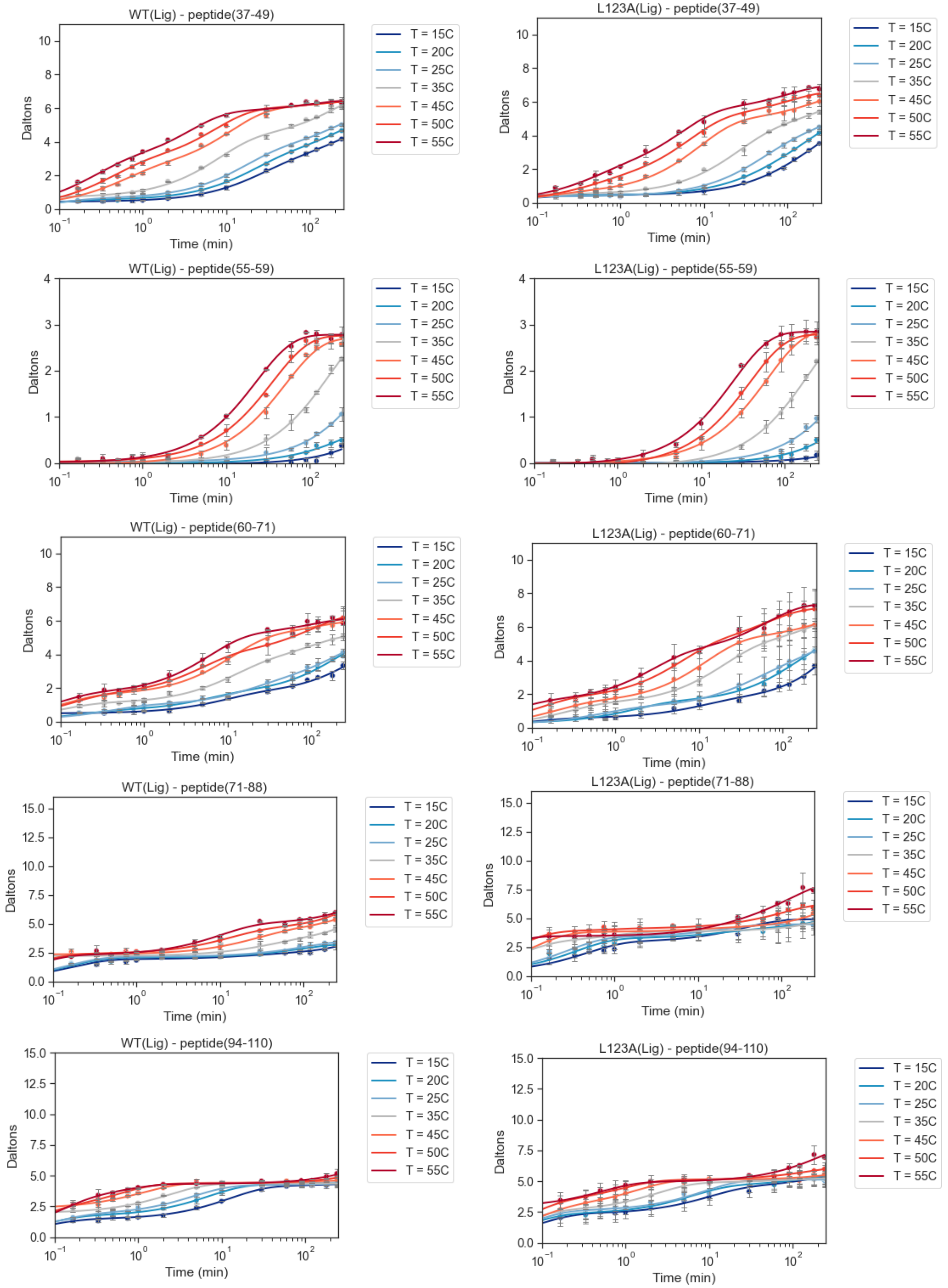
WT(Lig) - peptide(37-49)
Daltons
Time (min)
T = 15C
T = 20C
T = 25C
T = 35C
T = 45C
T = 50C
T = 55C
L123A(Lig) - peptide(37-49)
Daltons
Time (min)
T = 15C
T = 20C
T = 25C
T = 35C
T = 45C
T = 50C
T = 55C
WT(Lig) - peptide(55-59)
Daltons
Time (min)
T = 15C
T = 20C
T = 25C
T = 35C
T = 45C
T = 50C
T = 55C
L123A(Lig) - peptide(55-59)
Daltons
Time (min)
T = 15C
T = 20C
T = 25C
T = 35C
T = 45C
T = 50C
T = 55C
WT(Lig) - peptide(60-71)
Daltons
Time (min)
T = 15C
T = 20C
T = 25C
T = 35C
T = 45C
T = 50C
T = 55C
L123A(Lig) - peptide(60-71)
Daltons
Time (min)
T = 15C
T = 20C
T = 25C
T = 35C
T = 45C
T = 50C
T = 55C
WT(Lig) - peptide(71-88)
Daltons
Time (min)
T = 15C
T = 20C
T = 25C
T = 35C
T = 45C
T = 50C
T = 55C
L123A(Lig) - peptide(71-88)
Daltons
Time (min)
T = 15C
T = 20C
T = 25C
T = 35C
T = 45C
T = 50C
T = 55C
WT(Lig) - peptide(94-110)
Daltons
Time (min)
T = 15C
T = 20C
T = 25C
T = 35C
T = 45C
T = 50C
T = 55C
L123A(Lig) - peptide(94-110)
Daltons
Time (min)
T = 15C
T = 20C
T = 25C
T = 35C
T = 45C
T = 50C
T = 55C

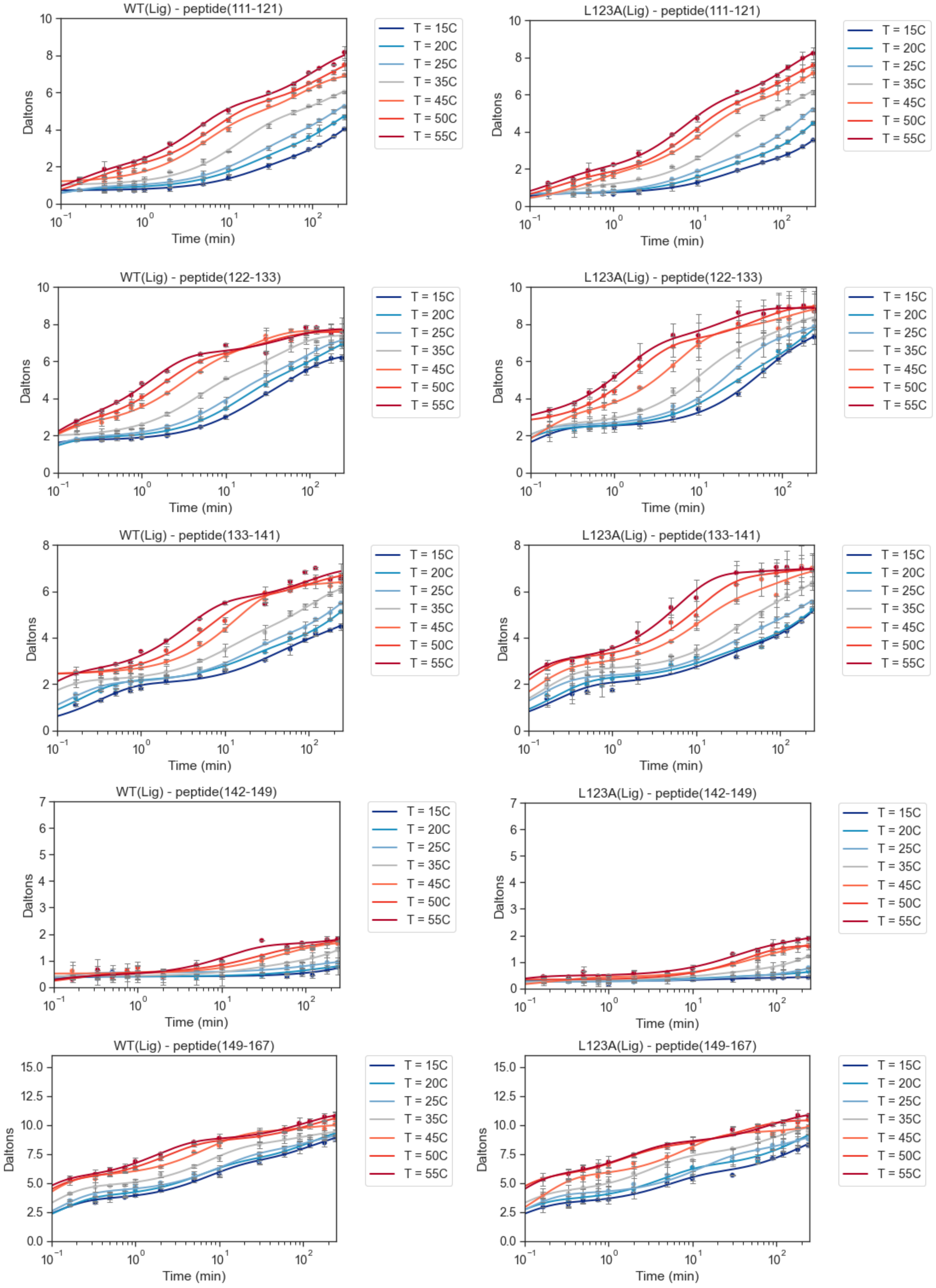
WT(Lig) - peptide(111-121)
L123A(Lig) - peptide(111-121)
WT(Lig) - peptide(122-133)
L123A(Lig) - peptide(122-133)
WT(Lig) - peptide(133-141)
L123A(Lig) - peptide(133-141)
WT(Lig) - peptide(142-149)
L123A(Lig) - peptide(142-149)
WT(Lig) - peptide(149-167)
L123A(Lig) - peptide(149-167)
Daltons
Time (min)
T = 15C
T = 20C
T = 25C
T = 35C
T = 45C
T = 50C
T = 55C

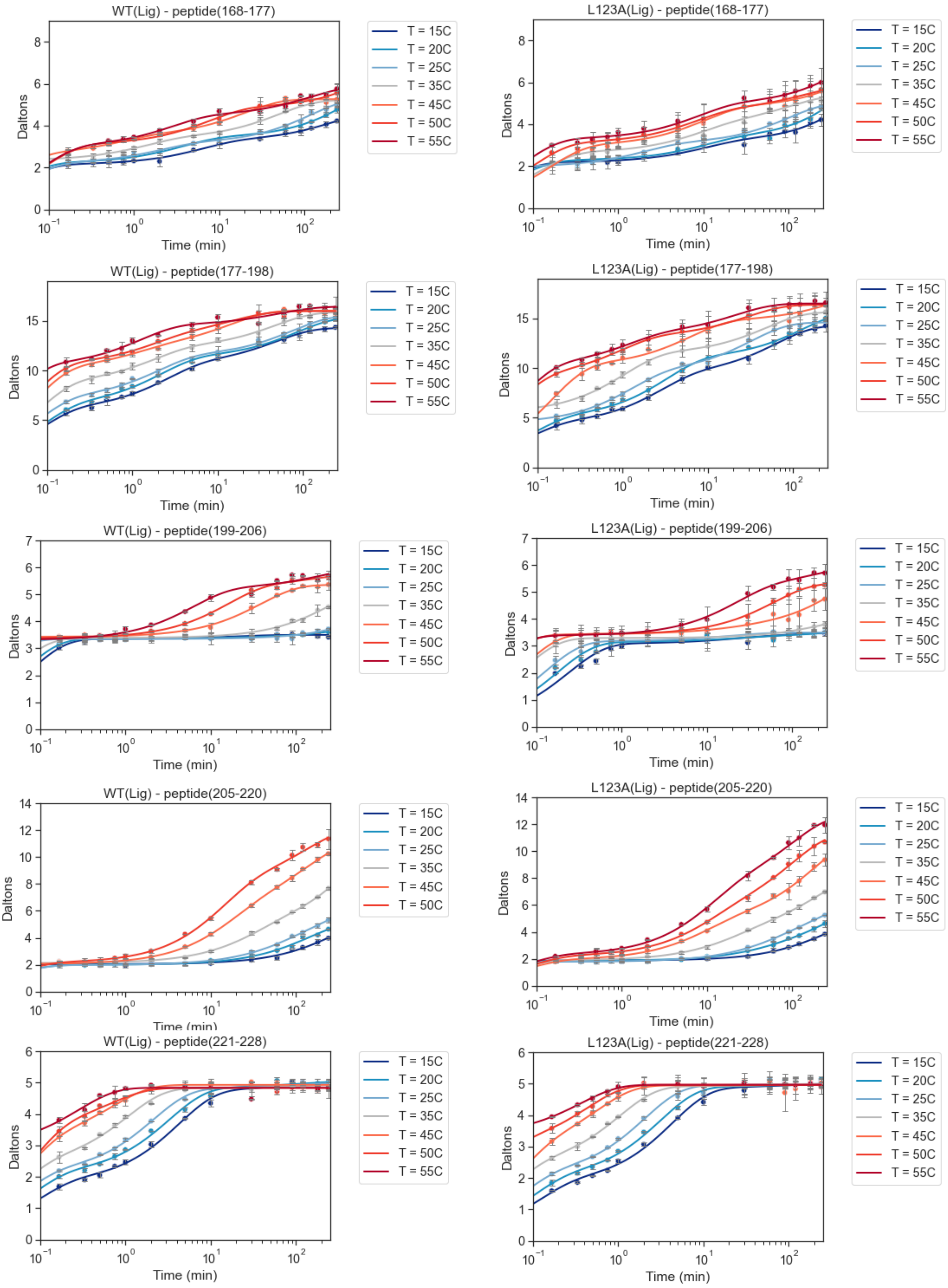
WT(Lig) - peptide(168-177)
L123A(Lig) - peptide(168-177)
WT(Lig) - peptide(177-198)
L123A(Lig) - peptide(177-198)
WT(Lig) - peptide(199-206)
L123A(Lig) - peptide(199-206)
WT(Lig) - peptide(205-220)
L123A(Lig) - peptide(205-220)
WT(Lig) - peptide(221-228)
L123A(Lig) - peptide(221-228)
Daltons
Time (min)
T = 15C
T = 20C
T = 25C
T = 35C
T = 45C
T = 50C
T = 55C

Table S10. Final fitting parameters obtained from the three-exponential fits for D-uptake vs. ln(*Time*) plots of replicate 1 for the WT(L) construct of Mt-OMPDC. These values were determined after fitting the data using the initial guess parameters.

Peptide	Temperature/°C	N_{obs} (Da)	A (Da)	B (Da)	C (Da)	k_1 (min^{-1})	k_2 (min^{-1})	k_3 (min^{-1})
10-17	15	5.026691507	1.410234	2.787494	0.796999	3.914423	0.062789	0.01
	20	5.67790348	1.657742	3.117508	0.875993	5.98677	0.081038	0.002144
	25	5.186252864	1.691445	2.994261	0.496963	9.952082	0.129549	0.01
	35	5.006170644	1.869587	2.904811	0.231708	20.37066	0.277339	0.02
	45	5.367187472	2.179972	2.742777	0.444169	15.47999	0.594206	2.08E-19
	50	6	2.528981	2.265738	1.203681	11.06124	0.683344	0.000735
	55	6	2.540575	2.275396	1.183938	14.64799	1.44101	0.000702
28-36	15	6.999999999	4.492518	0.1	0.000585	1.064545	0.610087	5.31021
	20	4.106228646	3.478189	0.009639	4.52E-18	1.382098	1.324766	1.359866
	25	3.455874723	9.289267	0.2	0.005	1.325044	0.509202	1.614505
	35	3.813299078	18.53855	0.039467	0.005	1.559465	1.111358	1.142418
	45	4.019635505	20.76349	0.06852	0.005	1.493631	1.614954	0.910967
	50	7	100	0.100855	0.001321	1.50864	1.521647	3.969713
	55	4.464018935	22.46815	0.116408	0.01	1.661964	1.452672	1.349456
37-49	15	4.923892	0.405286	2.041401	2.477116	15.70626	0.0452	0.005
	20	5.468232	0.569933	2.256083	2.62931	6.364624	0.050673	0.005
	25	6.033624	0.521478	2.619423	2.892616	10.97659	0.075667	0.005
	35	7.153231	0.672326	3.498282	2.982623	100	0.124003	0.005
	45	6.406441	2.171029	3.524488	0.576139	1.667941	0.087812	0.01
	50	10	2.488291	3.526829	3.916658	2.5	0.131328	0.000502
	55	6.466605	2.598467	3.198846	0.633521	4.35335	0.26573	0.01
55-59	15	2.999749	2.44E-17	2.25E-17	3.066875	40.19854	1.118184	0.00046
	20	0.637709	1.14E-14	1.76E-14	0.667707	78.42981	0.500016	0.005664
	25	2.339722	3.60E-09	1.52E-18	2.341101	72.84565	1.48238	0.002261
	35	2.999999	7.26E-23	4.64E-13	3.041112	71.58692	2.499763	0.005671
	45	2.74659	1.59E-20	2.48E-11	2.778726	3.033285	2.499573	0.019498
	50	2.832856	4.07E-24	0.042313	2.792725	99.06583	2.5	0.024422
	55	2.744307	1.58E-17	7.32E-11	2.75961	99.99374	0.500008	0.044095
60-71	15	3.889189	0.44596	1.113018	2.330211	98.24682	0.124361	0.005
	20	4.194814	1.144069	2.94394	3.57E-13	1.384423	0.010271	0.005
	25	4.981531	0.527646	1.165089	3.288529	12.89299	0.189198	0.005
	35	5.432701	1.023228	2.161942	2.24778	17.06547	0.111213	0.01
	45	5.768763	1.541199	3.224281	0.974585	6.893794	0.089454	0.01
	50	7.448353	1.780454	2.884703	2.754908	5.302554	0.111969	0.004031
	55	6.73016	1.697528	3.185934	1.848365	14.52265	0.225889	0.009577
71-88	15	15	1.92412	0.352406	12.6924	5.998979	0.05	0.000283
	20	4.400874	2.183974	1.077536	1.089346	5.43116	0.007947	3.14E-15
	25	4.241799	2.015293	0.263255	1.947447	6.65499	0.1	0.00406
	35	15	2.176996	1.502764	11.32024	100	0.016908	0.000384
	45	5.414664	2.299026	2.112625	1.003013	100	0.028373	0.005
	50	7.009289	2.461852	1.910844	2.635103	14.01422	0.076858	0.002691
	55	15	2.307903	2.531668	10.16254	16.93247	0.089254	0.000474
94-110	15	4.844608	1.469877	2.669499	0.703525	9.284002	0.080384	0.00312
	20	4.521063	1.885932	2.485008	0.139317	8.423437	0.143035	0.005
	25	4.47851	1.668903	2.562819	0.245206	12.89275	0.263302	0.005
	35	5.275301	2.167861	2.341492	0.765948	100	0.317429	3.87E-06
	45	4.454721	2.035748	2.370257	0.048716	100	0.960422	2.50E-15
	50	14	2.100074	2.302285	9.597644	19.35406	1.745122	0.000175
	55	14	3.531855	0.782121	9.691878	8.649207	1.249322	0.000399
	15	8.964677	0.749362	1.25536	6.959551	12.78462	0.060745	0.001485
	20	5.764156	1.014383	1.465199	3.255539	5.524193	0.049391	0.005
	25	6.46544	0.855077	1.994525	3.615464	11.9066	0.06864	0.005
	35	6.851453	1.0167	3.361226	2.473527	100	0.067892	0.005

111-121	45	6.867128	1.265761	2.983195	2.58434	6.148992	0.166499	0.015029
	50	9	1.830246	3.37939	3.724887	3.941412	0.134316	0.004183
	55	9	1.791047	3.535837	3.682356	8.078871	0.231554	0.006323
122-133	15	6.237911	1.731062	1.146238	3.360601	25.89596	0.101006	0.017682
	20	9	1.941085	3.073814	3.981011	10.89942	0.062408	0.002872
	25	7.325849	1.792211	2.484013	3.049622	24.90092	0.149669	0.013861
	35	7.454103	2.02475	3.050091	2.379263	100	0.196813	0.024539
	45	7.596393	2.403903	3.264281	1.922977	11.12128	0.367644	0.05
	50	7.722762	2.372878	3.393055	1.956794	19.38992	0.58717	0.05
	55	7.642695	2.499376	3.871403	1.271549	16.93794	0.793083	0.020847
133-141	15	4.742285	1.838915	1.688625	1.151838	3.520579	0.034962	0.005
	20	7	2.154862	1.186333	3.621339	4.455252	0.055002	0.002665
	25	5.723207	1.950281	1.048458	2.714351	8.121059	0.152419	0.009735
	35	6.225755	2.180277	1.848632	2.196784	19.98667	0.115192	0.01
	45	6.365809	2.37037	3.599114	0.396227	17.37062	0.080154	0.01
	50	7	2.371257	3.174436	1.454306	100	0.165459	0.009087
	55	6.934753	2.422078	3.101132	1.411462	20.14387	0.295409	0.01
142-149	15	5.99998	0.447131	5.76E-22	5.555246	8.431624	0.1	0.000336
	20	0.899718	0.586673	0.30192	0.009171	9.278652	0.0059	1.12E-13
	25	1.313975	0.573086	1.02E-17	0.745506	9.443356	0.022945	0.003989
	35	5.999872	0.502205	0.216613	5.281054	100	0.031037	0.000745
	45	1.552981	0.452505	1.096401	1.38E-13	7.685585	0.019928	0.01
	50	5.999828	0.583858	0.939777	4.445524	4.0261	0.030412	0.000158
	55	5.999614	0.449469	1.127568	4.429666	12.44507	0.080928	0.000225
149-167	15	9.543161	3.621564	3.021055	2.89587	10.45856	0.130228	0.0075
	20	9.946761	3.945619	2.835299	3.148318	7.747487	0.158119	0.0075
	25	9.808687	4.000869	2.947272	2.854886	11.11765	0.177687	0.0075
	35	9.702671	4.712853	3.548519	1.442854	12.28067	0.133616	0.01
	45	9.853574	5.293992	3.70939	0.84663	13.38153	0.184348	0.01
	50	15	5.239434	3.595277	6.165175	17.21643	0.348762	0.001704
	55	11.275	5.61418	3.206984	2.453698	17.51196	0.497822	0.008437
168-177	15	4.39893	2.247393	0.999761	1.151807	23.49999	0.138494	0.007574
	20	6.02612	2.279442	1.202154	2.544251	15.34709	0.256398	0.003207
	25	5.529063	2.207442	1.129741	2.191803	21.36861	0.41569	0.007318
	35	5.26463	2.425394	0.985347	1.853889	100	0.698696	0.02
	45	5.230001	2.442879	1.14113	1.645991	100	1.5	0.048788
	50	8	3.107813	1.851844	3.039375	12.80013	0.112088	0.001238
	55	6.578179	2.992443	1.546761	2.038619	16.40085	0.383018	0.004181
199-206	15	4.881651	3.36651	0.148828	1.366018	14.14225	0.05	1.72E-23
	20	6	3.389049	0.013572	2.597068	15.52263	0.05	0.000444
	25	5.999996	3.412001	3.54E-11	2.587985	27.42552	0.005	0.000539
	35	5.383651	3.426123	4.61E-09	1.957425	20.40644	0.005	0.003885
	45	5.370253	3.4536	1.916652	5.07E-14	100	0.024344	0.005
	50	6	3.354088	1.847032	0.79888	100	0.071057	0.005
	55	5.815351	3.350342	1.969179	0.49583	100	0.149735	0.005
205-220	15	14	1.865183	0.206921	11.9277	17.78033	0.05	0.000643
	20	11.5	1.810026	0.522179	9.167798	20.38567	0.05	0.001125
	25	11.5	1.712718	0.891255	8.896027	100	0.05	0.001426
	35	11.5	1.926774	1.867781	7.705445	100	0.05	0.002743
	45	11.5	1.891512	4.231819	5.376505	19.50517	0.050212	0.005479
	50	12.73622	2.084964	5.184356	5.466826	20.78078	0.080771	0.006932
	55	11.50001	1.950073	2.925576	6.62551	16.67284	0.253667	0.031906
221-228	15	5.000231	1.787058	2.962253	0.250371	14.99992	0.22802	0.01
	20	5.035388	2.096759	2.790083	0.145677	11.8943	0.274626	0.01
	25	5.006369	2.105093	2.787721	0.113266	16.79988	0.438792	0.01
	35	4.982628	2.375728	2.510884	0.096016	100	0.907551	0.01
	45	5.050787	3.030479	1.96345	0.056849	21.85916	1.455336	1.40E-23
	50	6	3.897015	0.995016	1.108986	10.60636	0.838789	0.000156
	55	5.084231	3.004702	1.852072	0.227456	100	3.271589	6.28E-33

Table S11. Final fitting parameters obtained from the three-exponential fits for D-uptake vs. ln(*Time*) plots of replicate 2 for the WT(L) construct of Mt-OMPDC. These values were determined after fitting the data using the initial guess parameters.

Peptide	Temperature/°C	N_{obs} (Da)	*A* (Da)	*B* (Da)	*C* (Da)	k_1 (min^{-1})	k_2 (min^{-1})	k_3 (min^{-1})
10-17	15	5.019498	1.442938	2.631879	0.866394	3.221644	0.051486	0.01
	20	5.11163	1.367267	2.867645	0.869149	8.621678	0.105174	0.01
	25	5.031698	1.703181	2.763917	0.559909	9.806781	0.127235	0.01
	35	4.99439	1.789234	2.776989	0.428105	17.49367	0.369999	0.02
	45	4.966076	2.549712	2.411311	4.30E-06	10.48457	0.454695	9.98E-20
	50	4.942768	2.49548	2.334355	0.112519	12.05113	0.906663	0.010284
	55	5.431518	2.572658	2.150426	0.708263	14.19417	1.440474	0.003411
28-36	15	6.999995	1.089809	0.283786	5.491865	2.013989	0.075785	0.000675
	20	5.589272	1.187542	0.456529	3.90955	4.446313	0.1	0.001224
	25	7	1.352025	1.171887	4.44764	4.435772	0.016456	0.000227
	35	7	1.435084	1.44545	4.118046	10.86397	0.035726	0.000248
	45	7	1.63258	1.579456	3.788009	16.58877	0.065679	0.000675
	50	4.119845	1.543755	1.120154	1.455993	21.3227	0.121306	0.01
	55	4.507637	1.450732	1.510044	1.54682	15.57282	0.151678	0.01
37-49	15	5.258998	0.460823	1.992115	2.80606	100	0.042792	0.003922
	20	5.484949	0.49457	2.366829	2.62355	100	0.05234	0.005
	25	5.672979	0.677151	2.658239	2.337587	9.015279	0.057857	0.005
	35	6.967006	0.684251	3.366349	2.916406	100	0.133051	0.005
	45	6.451643	1.901985	3.708743	0.633904	2.165685	0.101946	0.01
	50	6.326327	2.529504	3.158344	0.52663	2.5	0.142145	0.01
	55	6.550228	2.51461	3.125722	0.867548	4.096108	0.297423	0.01
55-59	15	2.99971	0.015694	2.22E-15	2.984015	99.99969	0.51	0.000483
	20	2.020512	0.02059	4.15E-15	1.999921	99.99807	0.51	0.001319
	25	3	0.054997	2.43E-28	2.945691	8.116225	1.506578	0.001891
	35	3	0.04617	2.98E-16	2.95383	99.99841	0.500006	0.006083
	45	2.664817	8.60E-22	7.19E-13	2.683516	26.30799	0.902221	0.019786
	50	2.708831	0.02345	1.94E-09	2.685382	100	0.5	0.031171
	55	2.814858	0.037827	1.33E-10	2.777031	100	2.499999	0.043385
60-71	15	10	0.51809	1.113789	8.368121	100	0.103649	0.000928
	20	4.956071	0.497393	1.096304	3.362235	17.97184	0.156509	0.005
	25	10	0.997749	1.959063	6.995527	2.760321	0.028638	0.000817
	35	5.372073	1.160324	2.619946	1.55432	4.987283	0.059181	0.004804
	45	10	1.584479	3.770536	4.641586	11.78204	0.086302	0.000865
	50	6.277614	1.349886	2.339789	2.589804	11.56712	0.286924	0.01
	55	5.653467	1.555805	3.822559	0.270737	8.682737	0.127573	0.01
71-88	15	15	1.8507	0.384522	12.72574	5.805895	0.05	0.000198
	20	3.763317	1.784245	0.524021	1.447242	10.33659	0.1	0.004321
	25	4.512338	2.168644	1.209499	1.127879	7.284821	0.011276	2.79E-15
	35	15	2.237142	1.079509	11.68335	100	0.044126	0.000402
	45	15	2.388538	1.743841	10.86762	100	0.053258	0.000602
	50	15	2.288442	2.104539	10.60703	25.8753	0.085091	0.000671
	55	7.494784	2.211771	2.770957	2.512048	17.92588	0.115538	0.002006
94-110	15	4.108746	1.406834	2.701848	6.42E-05	36.21935	0.078796	6.94E-14
	20	14	1.523246	2.68242	9.794334	100	0.135104	6.94E-05
	25	4.311112	1.9093	2.315781	0.083764	9.657888	0.170527	0.005
	35	4.226934	1.66223	2.564701	2.57E-06	100	0.553247	5.23E-11
	45	14	2.582553	1.831716	9.585731	100	0.759467	0.000181
	50	14	2.065598	2.247325	9.687178	19.27845	1.671645	0.000243
	55	14	2.192498	2.156219	9.650942	11.63564	1.786172	0.000298
	15	5.242923	0.695263	1.230254	3.317406	100	0.043782	0.003954
	20	5.663759	0.662869	1.564134	3.436756	100	0.068097	0.005
	25	6.229532	0.93899	1.93524	3.349503	7.273667	0.05679	0.005
	35	6.812943	0.97528	3.2303	2.607362	100	0.087925	0.005

111-121	45	7.248282	1.194595	3.329669	2.724018	100	0.179107	0.009567
	50	7.437692	1.474221	3.319557	2.631037	6.787294	0.202005	0.010668
	55	7.915878	1.469874	3.270343	3.164352	6.19792	0.310245	0.013828
122-133	15	6.461984	1.713083	2.02936	2.71954	25.61621	0.066417	0.011057
	20	6.93861	1.717251	2.029559	3.191801	100	0.111701	0.012583
	25	7.724643	1.952571	2.989209	2.78295	13.25701	0.074971	0.006046
	35	7.33978	1.847665	2.599929	2.892186	100	0.29426	0.02071
	45	7.708852	2.577677	2.861924	2.269152	21.43758	0.358467	0.05
	50	7.390053	2.420309	3.50942	1.460985	14.10551	0.617403	0.03179
	55	7.846257	2.531188	3.734143	1.581027	12.91882	0.783793	0.017774
133-141	15	5.158209	1.951209	1.239646	1.82597	2.677252	0.024587	0.005
	20	5.979615	1.902885	1.176139	2.885678	7.538152	0.114617	0.005
	25	7	1.995063	1.59528	3.397958	7.930145	0.060197	0.003112
	35	6.448967	2.085733	1.361763	3.000479	13.41525	0.116606	0.01
	45	7	2.531235	3.705659	0.763106	100	0.066804	0.0015
	50	6.535759	2.429417	3.171434	0.934907	30.55913	0.146774	0.01
	55	7	2.446614	3.133317	1.419783	15.57299	0.282358	0.01
142-149	15	5.999887	0.353005	2.13E-39	5.646882	100	0.095001	0.000149
	20	5.782469	0.213586	0.210026	5.358856	100	0.020641	0.000263
	25	3.749899	0.515758	0.227751	3.006931	12.37253	0.023182	0.00018
	35	6	0.313135	0.497194	5.189671	100	0.05637	0.000335
	45	1.870857	0.56497	0.295197	1.010691	100	0.101441	0.01
	50	6	0.423957	0.881462	4.693394	8.790714	0.055202	0.000436
	55	6	0.393764	1.190008	4.417523	7.396101	0.092589	0.000186
149-167	15	9.198613	3.348223	2.76653	3.078982	11.578	0.169644	0.0075
	20	9.71705	3.511379	2.725305	3.475328	11.88005	0.200831	0.0075
	25	8.960287	4.301133	3.498294	1.129251	7.954037	0.057426	0.0075
	35	9.371462	4.521413	3.259942	1.584849	11.58403	0.143199	0.01
	45	15	5.71793	3.878884	5.401564	15.03657	0.110015	0.000574
	50	10.41909	5.167735	3.106578	2.145089	17.64751	0.497069	0.01238
	55	10.95066	4.858627	3.580543	2.511491	29.65038	0.611973	0.009753
168-177	15	5.387558	2.012673	1.202498	2.17239	23.99262	0.208347	0.002629
	20	7.065584	2.190064	1.181481	3.694039	100	0.261795	0.001831
	25	5.159077	2.148126	0.823533	2.187557	18.46383	0.6349	0.009184
	35	5.169543	2.244189	1.101723	1.823631	100	0.767175	0.02
	45	5.30838	2.738659	0.656073	1.913625	25.32837	1.5	0.05
	50	5.291713	2.957594	1.324372	1.008174	12.02746	0.286743	0.016103
	55	5.652088	2.9548	1.391721	1.303724	10.71082	0.353276	0.013777
199-206	15	4.49112	3.32997	0.150893	1.008608	12.99993	0.05	3.00E-17
	20	4.346679	3.333182	0.263966	0.749278	16.62797	0.00859	3.03E-19
	25	5.999979	3.277275	2.66E-12	2.722741	100	0.005	0.000634
	35	5.272008	3.339488	3.23E-10	1.932538	22.55147	0.005	0.003838
	45	6	3.386562	1.774269	0.839169	100	0.031644	0.001315
	50	5.514808	3.414756	2.100048	4.50E-15	28.47257	0.051521	0.005
	55	6	3.275408	1.908223	0.816369	100	0.161293	0.005
205-220	15	11.5	1.789482	0.163585	9.546933	100	0.05	0.000906
	20	11.5	1.652926	0.660273	9.186801	34.8638	0.05	0.00115
	25	11.5	1.798453	0.629412	9.072135	100	0.05	0.00168
	35	11.5	1.751933	2.248198	7.499869	100	0.05	0.002551
	45	11.5	1.937794	4.113667	5.448538	100	0.060228	0.006033
	50	11.5	1.97751	5.009989	4.512145	14.31837	0.07656	0.007717
221-228	15	5.061467	1.839765	2.9084	0.307337	9.124533	0.241982	0.01
	20	5.035562	2.043182	2.738367	0.253568	15.48385	0.332056	0.01
	25	4.995345	2.074752	2.64447	0.275969	18.6975	0.51923	0.01
	35	4.9	2.424612	2.348757	0.125846	13.12481	0.917966	0.007096
	45	4.944264	3.203356	1.667002	0.073036	13.0069	1.275514	7.75E-31
	50	5.046447	2.932077	1.83888	0.275491	100	2.304274	1.69E-22
	55	6	3.349646	1.445564	1.204754	16.19569	3.353729	0.000285

Table S12. Final fitting parameters obtained from the three-exponential fits for D-uptake vs. ln(*Time*) plots of replicate 1 for the L123A(L) construct of Mt-OMPDC. These values were determined after fitting the data using the initial guess parameters.

Peptide	Temperature/°C	N_{obs} (Da)	A (Da)	B (Da)	C (Da)	k_1 (min^{-1})	k_2 (min^{-1})	k_3 (min^{-1})
10-17	15	4.833095	1.384782	3.213628	0.121058	2.617336	0.032312	0.005
	20	5.543968	1.451097	2.754978	1.323571	6.375897	0.092548	0.005
	25	5.015123	1.653266	3.055527	0.284456	5.799292	0.088188	0.005
	35	5.031258	1.873393	2.615117	0.540622	10.95838	0.267303	0.01
	45	6	1.998439	2.751315	1.249695	12.39517	0.682665	0.000233
	50	4.944818	2.063049	2.532347	0.349423	25.79182	1.13999	0.016757
	55	4.946233	2.292726	2.353014	0.300494	100	1.575266	0.024639
28-36	15	1.853534	1.184708	0.420025	0.021719	1.24882	0.024287	1.98E-36
	20	7	1.040356	0.35335	5.538449	3.369248	0.1	0.001053
	25	3.180872	1.281003	0.898933	0.974152	4.104307	0.018915	0.005
	35	3.191224	1.303743	1.883804	7.63E-07	8.356679	0.02222	4.50E-07
	45	7	1.364155	1.327411	4.310233	11.26606	0.099988	0.0012
	50	7	1.444331	1.499013	4.056485	17.48023	0.070212	0.001321
	55	4.385257	1.331869	1.562226	1.491163	100	0.108657	0.007817
37-49	15	4.800513	0.458732	1.983721	2.358061	100	0.010316	0.003
	20	5.593463	0.415154	0.724807	4.453498	23.47928	0.037684	0.005
	25	5.323635	0.385124	2.46228	2.47623	100	0.0257	0.005
	35	5.80339	0.535184	3.659013	1.608735	15.49822	0.035567	0.005
	45	10	0.572318	4.168689	5.254739	8.853462	0.116084	0.000992
	50	6.540045	1.259391	3.477319	1.734174	2.5	0.140498	0.01
	55	6.883703	1.573692	3.599978	1.620296	2.5	0.181314	0.01
55-59	15	2.999888	1.30E-27	2.14E-27	3.02216	25.52919	0.050037	0.00013
	20	2.999995	5.24E-23	2.33E-23	3.038348	58.52069	4.999978	0.00059
	25	3	8.70E-37	4.88E-37	3.017456	8.10084	4.731711	0.001423
	35	2.994971	6.31E-14	2.32E-15	3.040808	89.54829	0.100266	0.005739
	45	2.749318	2.98E-21	2.30E-12	2.784909	20.39936	4.999994	0.01657
	50	2.726819	9.48E-20	1.08E-11	2.782022	13.24858	0.100001	0.026454
	55	2.69284	1.29E-19	1.04E-09	2.788015	5.000016	0.1	0.043721
60-71	15	7.070877	0.601744	1.141196	5.327977	16.44168	0.1	0.001904
	20	10	0.798919	1.329162	7.834868	4.762221	0.1	0.001974
	25	10	1.193503	2.88058	5.760087	1.708611	0.024558	0.001027
	35	10	1.391807	3.956726	4.627081	5.211051	0.043597	0.001018
	45	7.233592	1.509575	3.592203	2.124639	7.35976	0.13488	0.01
	50	7.994112	2.025263	3.093366	2.867787	6.041239	0.133768	0.011258
	55	8.013398	1.589878	2.893443	3.530076	100	0.468599	0.01993
71-88	15	5.355183	2.628725	2.441677	0.000121	3.015459	0.028678	5.52E-30
	20	14.99998	3.333777	1.18619	10.22438	3.152276	0.05	0.000251
	25	14.83819	3.273552	0.907716	10.54973	4.785201	0.05	2.62E-05
	35	5.150511	3.147446	1.115436	0.887516	14.83478	1.16526	0.013267
	45	15	3.735712	0.752792	10.51114	11.53205	1.245691	0.000482
	50	6.755698	4.347862	2.20E-10	2.40654	13.34661	0.050003	0.007181
	55	8.75267	3.950722	0.029783	4.772166	99.9997	0.073029	0.008017
94-110	15	6.262902	2.514776	2.183661	1.564497	21.75916	0.164788	0.005
	20	13.99998	2.833558	2.285514	8.878705	13.18119	0.161644	0.000448
	25	5.537273	2.972194	2.398174	0.161143	11.08725	0.11407	0.005
	35	5.680525	3.055972	2.076941	0.547616	16.25707	0.46441	0.01
	45	14	3.154706	2.109292	8.735642	13.56605	0.843105	0.000141
	50	6.860957	3.160849	2.142001	1.558055	22.4602	1.902495	0.00394
	55	8.495182	3.439142	1.591453	3.464587	100	2.000255	0.004359
	15	6.925484	0.640569	1.17424	5.110675	100	0.078575	0.001648
	20	9	0.66175	1.429022	6.909217	21.6407	0.075489	0.001781
	25	6.485105	0.582751	1.522327	4.380027	100	0.123022	0.005
	35	6.878462	0.961348	3.370911	2.527569	7.045742	0.053523	0.005

111-121	45	9	1.309644	3.585037	4.034029	3.393818	0.096087	0.002976
	50	7.956805	1.52163	3.607896	2.829692	4.520112	0.106823	0.006886
	55	8.284886	1.505986	3.740349	3.006365	5.57788	0.158311	0.00951
122-133	15	7.147631	2.622856	4.524614	1.40E-13	13.95294	0.016163	0.005
	20	9	2.573631	1.954715	4.471411	17.08383	0.085099	0.005
	25	8.388926	2.666983	3.870485	1.850597	14.81987	0.040407	0.00344
	35	8.064373	2.869034	3.611541	1.582329	14.18067	0.062875	0.01
	45	9	3.137779	3.920319	1.93881	8.091002	0.207568	0.003465
	50	8.65238	2.818756	3.337276	2.496348	100	0.741761	0.02
	55	8.319589	3.034234	3.141607	2.143748	99.99968	0.807577	0.05
133-141	15	7	1.948931	1.418638	3.606928	7.676133	0.075	0.002847
	20	7	2.408019	1.064082	3.498491	6.591348	0.075	0.002758
	25	6.245007	2.430743	1.466512	2.328157	9.175641	0.043944	0.005
	35	6.720663	2.72893	2.102476	1.881488	10.04274	0.029824	0.005
	45	7	2.78424	2.382924	1.833356	9.427001	0.133131	0.004855
	50	7	3.268035	3.36344	0.363629	10.92844	0.052733	0.00083
	55	6.629059	3.058547	3.570208	8.42E-16	17.95261	0.116022	3.47E-06
142-149	15	0.472484	0.236764	0.143835	0.091884	100	0.1	0.005
	20	6	0.297762	0.120603	5.581636	38.29925	0.082078	0.00022
	25	6	0.242957	0.259496	5.497547	100	0.053256	0.000209
	35	6	0.314426	0.648112	5.037338	15.77586	0.021154	0.000189
	45	6	0.413152	0.861542	4.723919	9.26284	0.026567	0.000319
	50	1.641333	0.30388	1.192598	0.145086	14.46811	0.020478	2.26E-12
	55	2.056099	0.444959	0.874803	0.736337	100	0.039627	0.005
149-167	15	9.552767	3.397558	2.463865	3.690465	15.22218	0.2	0.005
	20	14.2019	3.80329	2.900011	7.496261	13.44096	0.220463	0.001969
	25	9.677442	4.612054	3.559126	1.485993	9.705329	0.057593	0.005
	35	10.1985	4.662319	2.654738	2.879244	14.08988	0.265055	0.01
	45	15	5.562464	3.362298	6.058387	7.299681	0.168511	0.000639
	50	10.46645	5.421984	2.120163	2.924145	16.91887	1.13264	0.025
	55	10.99935	5.47206	2.571337	2.955932	22.67583	0.887286	0.016764
168-177	15	8	2.074082	0.891709	5.034209	100	0.1	0.00097
	20	8	2.173652	0.942652	4.883604	24.42823	0.1	0.001447
	25	4.916431	1.724445	1.213971	1.978015	100	0.619676	0.01
	35	5.595704	2.668786	2.19052	0.718041	7.396966	0.033636	1.67E-24
	45	8	2.757798	1.565462	3.653738	5.291702	0.134938	0.000952
	50	8	3.011692	1.478502	3.495755	8.233457	0.054292	0.001215
	55	8	3.130655	1.606046	3.262078	15.46249	0.081756	0.0012
199-206	15	3.519655	2.821079	0.286827	0.318201	4.741163	0.05	0.005
	20	6	2.970602	0.192867	2.784224	5.989529	0.05	0.000381
	25	3.581809	3.023413	0.356405	0.185251	7.909176	0.037767	3.85E-05
	35	4.837313	3.158003	0.741624	0.937151	17.07224	0.005459	3.18E-13
	45	6	3.298655	0.1193	2.581439	11.67581	0.1	0.001971
	50	5.226049	3.275889	1.950166	3.83E-09	25.06516	0.013762	6.45E-07
	55	6	3.21659	1.9329	0.850509	100	0.039373	0.002337
205-220	15	13.99998	1.817393	0.799302	11.38329	100	0.012953	0.000467
	20	14	1.822346	1.065044	11.11261	100	0.023853	0.000789
	25	6.799085	1.737316	1.659079	3.40269	100	0.022291	0.003291
	35	9.194224	1.859277	2.730931	4.604012	26.8502	0.037999	0.002976
	45	11.40483	1.893508	2.848652	6.662389	15.99235	0.115315	0.004501
	50	11.10583	2.314662	2.856674	5.930427	9.496818	0.100029	0.01
221-228	15	5	1.743212	2.932822	0.320462	9.946275	0.259158	0.01
	20	5	1.860558	2.850913	0.287532	11.64807	0.382652	0.01
	25	5	2.007409	2.760045	0.232135	14.80895	0.559006	0.01
	35	4.987049	2.301419	2.408897	0.276797	17.16572	1.162491	0.01
	45	4.909119	2.711152	2.111651	0.086432	15.19569	1.987795	2.21E-15
	50	5	2.807513	2.003553	0.188934	42.1163	2.444335	0.000917
	55	4.99928	3.496296	1.326337	0.176651	24.75542	3	2.93E-27

Table S13. Final fitting parameters obtained from the three-exponential fits for D-uptake vs. ln(*Time*) plots of replicate 2 for the L123A(L) construct of Mt-OMPDC. These values were determined after fitting the data using the initial guess parameters.

Peptide	Temperature/°C	N_{obs} (Da)	*A* (Da)	*B* (Da)	*C* (Da)	k_1 (min^{-1})	k_2 (min^{-1})	k_3 (min^{-1})
10-17	15	5.498016	1.473061	2.929555	1.055796	3.100357	0.046274	0.005
	20	5.459729	1.480403	2.944006	1.007155	5.025492	0.08416	0.005
	25	5.232689	1.590459	3.260253	0.369687	8.420692	0.105861	0.005
	35	5.206534	1.714092	3.127043	0.36496	16.01114	0.264983	0.01
	45	5.222965	2.102716	2.777414	0.342622	17.26309	0.649285	0.034111
	50	5.162035	2.582379	2.578773	8.59E-06	12.96326	0.752107	1.59E-14
	55	6	2.533758	2.536989	0.929189	19.50414	1.303497	0.002135
28-36	15	7	1.087868	0.466018	5.434106	3.674019	0.1	0.000692
	20	2.949823	1.265708	1.58361	3.73E-07	2.237127	0.006878	0.005
	25	5.285517	1.08473	0.604168	3.581065	7.387515	0.1	0.002034
	35	3.721047	1.380741	1.202511	1.125722	7.512634	0.040668	0.004125
	45	7	1.337596	1.419184	4.243416	12.22905	0.08903	0.001536
	50	4.188575	1.45295	1.50397	1.231479	16.65534	0.084571	0.009271
	55	4.847395	1.542044	1.334351	1.969946	12.86251	0.147562	0.01
37-49	15	5.859446	0.411785	0.805965	4.649311	8.279624	0.01398	0.003
	20	10	0.406538	2.644338	6.949124	100	0.01463	0.00072
	25	6.667594	0.331635	3.080387	3.255572	100	0.021506	0.001631
	35	6.297478	0.424176	3.336745	2.535091	9.386078	0.051352	0.005
	45	6.154423	0.422683	3.56017	2.170513	10.76534	0.155043	0.018472
	50	6.790975	0.624113	4.438568	1.717559	6.95623	0.195036	0.01
	55	7.20029	1.624457	3.887855	1.6571	2.5	0.212318	0.01
55-59	15	3	0.039248	5.56E-30	2.960752	42.33663	4.999738	0.000233
	20	3	0.025785	3.27E-31	2.974215	100	3.697391	0.000762
	25	3	0.003629	2.59E-32	2.996371	95.94433	0.100945	0.001605
	35	3	5.62E-17	2.71E-16	3.078173	69.0517	0.100007	0.005076
	45	3	0.020918	8.21E-18	2.979082	88.53743	4.999999	0.01609
	50	2.84839	1.13E-18	1.37E-13	2.946304	5.00677	4.563998	0.026528
	55	2.999755	0.02251	1.04E-14	2.977245	99.99561	4.999998	0.038147
60-71	15	10	0.508295	0.828872	8.665699	6.975046	0.1	0.001166
	20	4.621327	1.173675	3.246233	1.04E-10	0.559642	0.007902	0.005
	25	4.556667	0.568098	1.086774	2.884404	6.429733	0.1	0.005
	35	6.206756	1.192569	2.609405	2.355029	3.421736	0.088577	0.0075
	45	10	1.243595	3.580803	5.166078	4.950263	0.06667	0.00031
	50	6.437785	1.440357	3.332006	1.665197	14.53415	0.189266	0.018323
	55	6.974578	1.596869	2.768877	2.608071	8.207978	0.235597	0.009036
71-88	15	5.359757	2.64217	1.599651	0.891078	1.770045	0.027644	6.33E-32
	20	5.1641	2.709014	2.47E-06	2.152901	2.192694	0.005	0.002551
	25	6.508321	3.113176	1.235808	1.872565	2.582883	0.044095	4.50E-45
	35	15	2.5077	0.783222	11.70733	13.41282	1.5	0.000244
	45	15	3.402808	0.328236	11.27559	10.98646	0.05	0.00049
	50	6.076258	3.374454	0.612744	2.089044	21.80572	0.621902	0.009882
	55	8.123193	2.937947	1.616058	3.568841	19.31873	0.05	0.005637
94-110	15	5.347383	2.10491	2.027901	1.216249	7.709756	0.070111	0.005
	20	5.166255	1.820352	2.40814	0.937365	17.92639	0.146561	0.005
	25	7.033637	2.176915	2.633974	2.222739	29.31628	0.119433	8.32E-22
	35	5.077533	1.961022	2.692128	0.424382	35.5406	0.387808	0.003133
	45	5.511492	2.300586	2.617096	0.594685	12.45481	0.730209	0.006608
	50	5.929515	2.787869	2.105093	1.036381	15.71881	0.866009	0.006909
	55	8.09696	2.273365	2.576649	3.246946	100	1.844282	0.004578
	15	9	0.683319	1.386589	6.939299	8.688024	0.037571	0.00104
	20	5.48437	0.631493	1.2309	3.621977	100	0.095885	0.005
	25	6.680618	0.653812	1.504198	4.522607	100	0.104285	0.005
	35	7.351684	0.952419	2.808363	3.515724	3.135263	0.068957	0.005

111-121	45	7.759398	1.470757	3.329889	2.860155	2	0.091193	0.007298
	50	8.016928	1.332138	3.591999	3.080719	6.402758	0.136581	0.009578
	55	9	1.727691	3.538205	3.722688	5.86908	0.164154	0.007999
122-133	15	8.729506	2.281776	2.692963	3.749995	8.495663	0.02964	0.005
	20	9	2.195333	2.917747	3.884277	12.9505	0.049171	0.005
	25	9	2.350752	4.811842	1.837222	19.00771	0.041042	0.003164
	35	9	2.200249	4.164037	2.635142	16.87099	0.13806	0.01
	45	9	2.996031	5.713479	0.270066	7.99436	0.12665	0.01
	50	9	2.421433	5.45361	1.124957	99.99998	0.438937	0.02
	55	9	2.748162	5.07199	1.179656	18.67048	0.705688	0.05
133-141	15	7	1.890226	1.115695	3.937454	3.836754	0.075	0.002916
	20	6.343301	1.927937	0.959504	3.318283	2.994905	0.075	0.004231
	25	6.476434	2.078495	1.628455	2.745715	7.042652	0.072067	0.005
	35	7	2.457257	2.61237	1.888119	5.136133	0.03997	0.005
	45	7	2.804329	4.178233	6.95E-13	7.6015	0.04569	0.01
	50	7	2.800093	4.197977	2.18E-12	13.68969	0.117809	0.01
	55	7	2.790636	4.207921	1.12E-11	13.68933	0.220585	0.01
142-149	15	0.806004	0.326857	0.100466	0.388947	7.655174	0.009978	1.04E-17
	20	0.718848	0.305721	0.223554	0.189573	100	0.024332	3.95E-33
	25	6	0.253417	0.047481	5.699102	100	0.1	0.000328
	35	6	0.308513	0.114199	5.574564	6.763312	0.1	0.000616
	45	1.72914	0.443932	1.251424	2.16E-12	1.71557	0.011788	0.005
	50	2.064239	0.355546	1.362097	0.346597	100	0.024882	1.26E-19
	55	2.136393	0.501536	1.050984	0.585521	9.983353	0.033755	0.005
149-167	15	9.521777	3.011895	2.417633	4.088609	10.47615	0.190016	0.005
	20	9.993962	3.101702	2.974963	3.916053	15.36633	0.214272	0.005
	25	9.472757	3.359953	3.143396	2.965602	12.95851	0.120638	0.005
	35	9.824657	3.592487	3.586594	2.642545	14.61806	0.331835	0.01
	45	9.908944	5.370405	4.241463	0.300719	7.297831	0.088277	0.004625
	50	10.30838	5.095029	3.466227	1.74711	24.79388	0.442374	0.025
	55	11.53935	5.284178	3.387072	2.866165	12.74278	0.397554	0.005439
168-177	15	5.212917	2.29624	1.016493	1.900153	17.77206	0.1	0.003709
	20	8	2.353238	1.154746	4.490075	13.3851	0.1	0.001369
	25	5.188068	2.166116	0.994235	2.027717	100	0.414816	0.009812
	35	5.769865	2.148662	1.514129	2.107074	100	0.638556	0.01
	45	6.044068	3.139251	1.855698	1.040309	8.011476	0.085854	0.01
	50	5.956167	3.176196	1.952543	0.824218	12.89606	0.16207	0.007711
	55	7.301579	3.368463	1.714374	2.216547	12.76933	0.187013	0.004093
199-206	15	3.572328	3.119157	0.233162	0.0894	3.816573	0.05	0.005
	20	6	3.203508	0.192824	2.545755	5.396725	0.05	0.00013
	25	3.63242	3.307818	0.284142	0.023104	8.076968	0.05	1.09E-16
	35	5.763421	3.422304	0.019605	2.318816	13.24354	0.05	0.000917
	45	5.202179	3.589801	1.612482	9.55E-14	20.66206	0.009523	0.005
	50	5.448755	3.562586	1.886169	3.69E-13	100	0.019102	0.005
	55	6	3.553848	1.947008	0.499142	24.61051	0.045182	0.005
205-220	15	5.566311	1.926594	5.76E-10	3.639826	16.97	0.005	0.003261
	20	5.325986	1.880885	0.566794	2.878307	100	0.01882	0.005
	25	6.274695	1.813825	1.236833	3.224037	100	0.016724	0.005
	35	8.589951	1.89715	1.656845	5.035701	17.42176	0.072374	0.004949
	45	10.92438	1.929743	2.676652	6.317561	11.92512	0.111191	0.006783
	50	11.68554	2.051149	3.280833	6.353538	23.43152	0.107826	0.01
	55	13.02228	2.300123	4.925375	5.794142	12.28279	0.09749	0.01
221-228	15	5	1.842959	3.150355	6.30E-13	9.098418	0.218788	0.01
	20	5	2.015442	2.982968	4.34E-15	11.98969	0.29944	0.01
	25	5	1.999756	3.000137	4.36E-13	19.44437	0.507998	0.01
	35	5	2.271383	2.728617	2.81E-13	100	0.934002	0.01
	45	5	2.685667	2.314316	7.15E-13	23.65192	1.82278	0.01
	50	5	2.896658	2.103339	7.30E-33	99.99862	2.77248	0.01
	55	5.566311	1.926594	5.76E-10	3.639826	16.97	0.005	0.003261

Figure S10. Arrhenius-like plots to obtain HDX activation energy ($E_a(k_{HDX})$) for 6-azaUMP bound forms of Mt-OMPDC.The left panel shows the data for the WT(L), while the right panel represents the L123A(L). Plots in red are generated using $k_{HDX} = ((Bk_2 + Ck_3)/N_T)$ as the weighted rate constant and plot in purple are generated using $k_{HDX} = (Bk_2/N_T)$. In most of the plot, purple data ((Bk_2/N_T)) point is shadowed under red data (weighted average rate constant) points. Activation energy values are reported in kcal/mol, with standard deviations in parentheses. These errors were calculated from biological replicates using linear regression of the Arrhenius plot

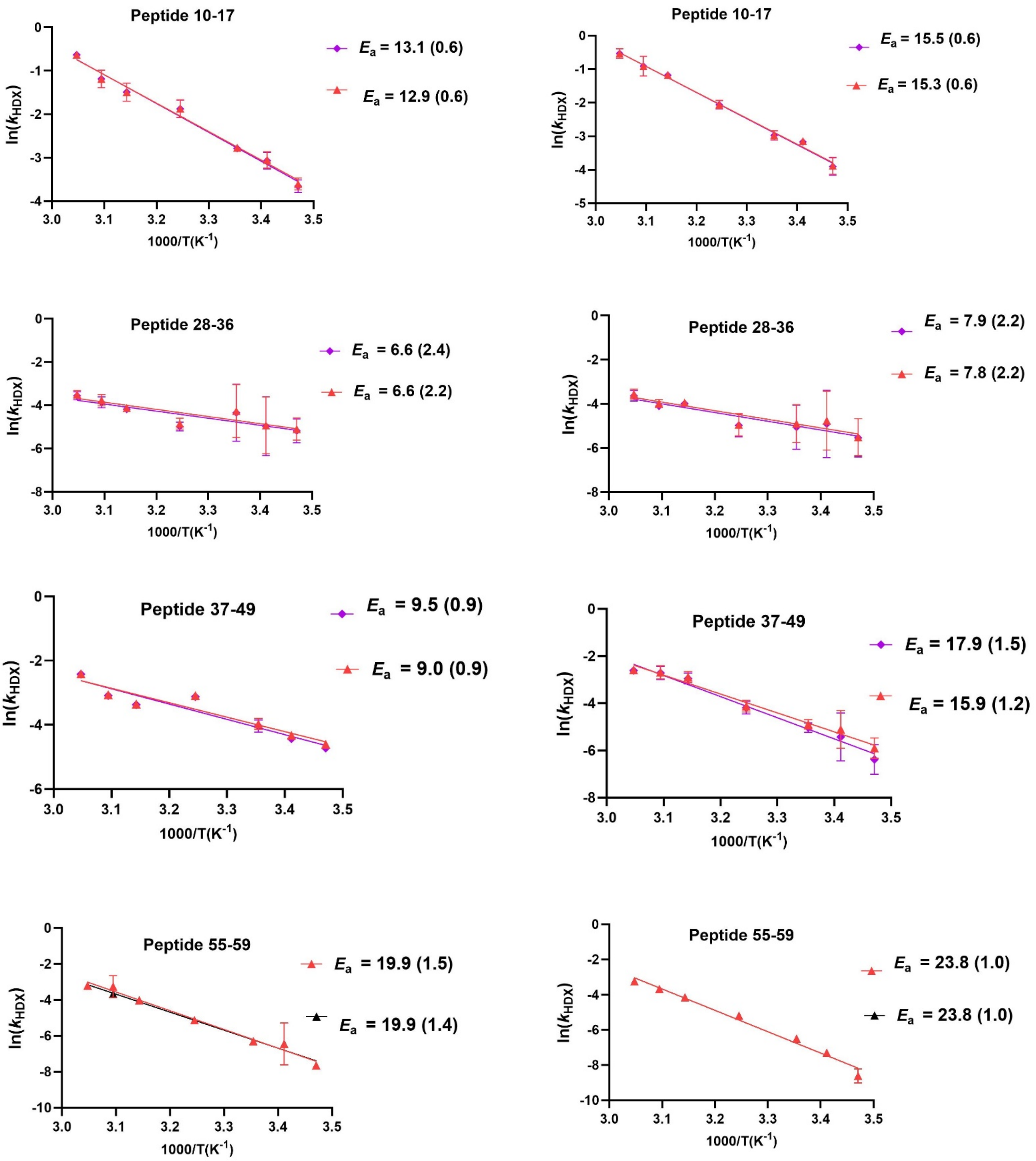

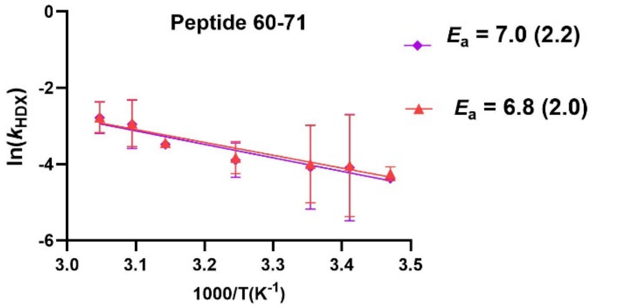
Peptide 60-71
E_a = 7.0 (2.2)
E_a = 6.8 (2.0)
ln(k_{HDX})
1000/T(K^{-1})

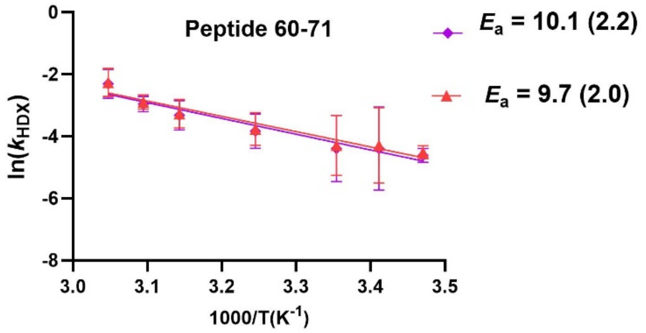
Peptide 60-71
E_a = 10.1 (2.2)
E_a = 9.7 (2.0)
ln(k_{HDX})
1000/T(K^{-1})

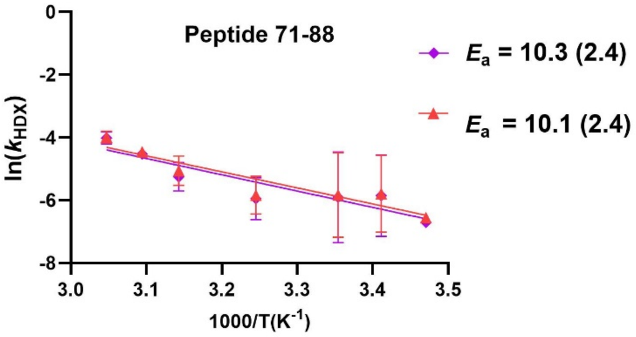
Peptide 71-88
E_a = 10.3 (2.4)
E_a = 10.1 (2.4)
ln(k_{HDX})
1000/T(K^{-1})

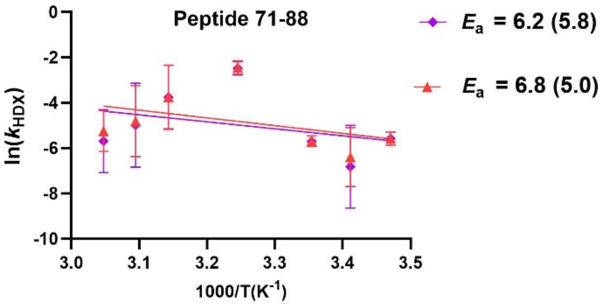
Peptide 71-88
E_a = 6.2 (5.8)
E_a = 6.8 (5.0)
ln(k_{HDX})
1000/T(K^{-1})

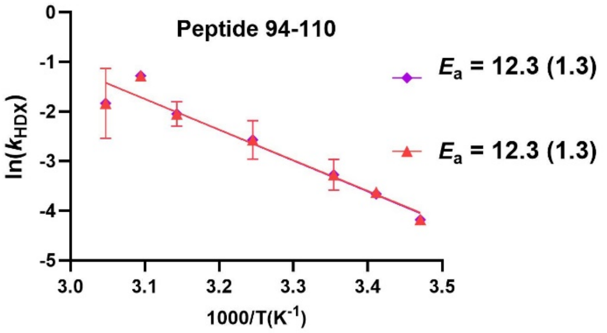
Peptide 94-110
E_a = 12.3 (1.3)
E_a = 12.3 (1.3)
ln(k_{HDX})
1000/T(K^{-1})

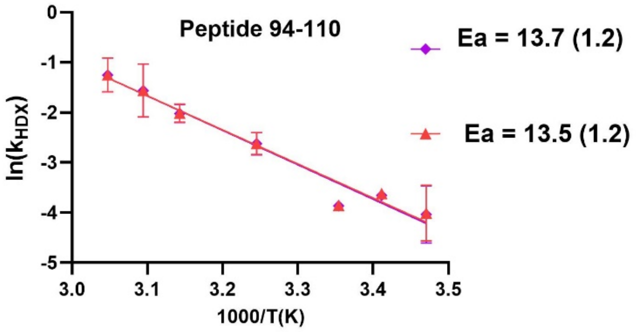
Peptide 94-110
Ea = 13.7 (1.2)
Ea = 13.5 (1.2)
ln(k_{HDX})
1000/T(K)

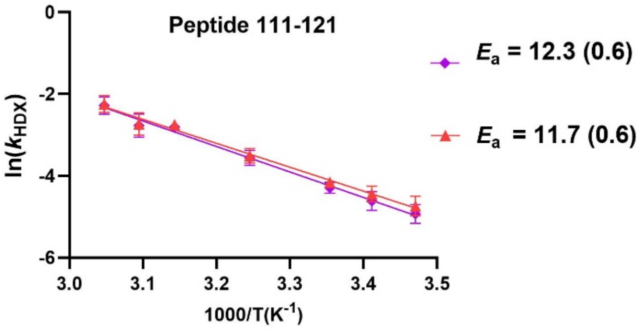
Peptide 111-121
E_a = 12.3 (0.6)
E_a = 11.7 (0.6)
ln(k_{HDX})
1000/T(K^{-1})

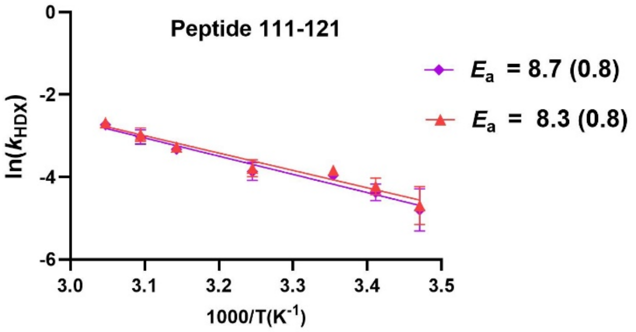
Peptide 111-121
E_a = 8.7 (0.8)
E_a = 8.3 (0.8)
ln(k_{HDX})
1000/T(K^{-1})

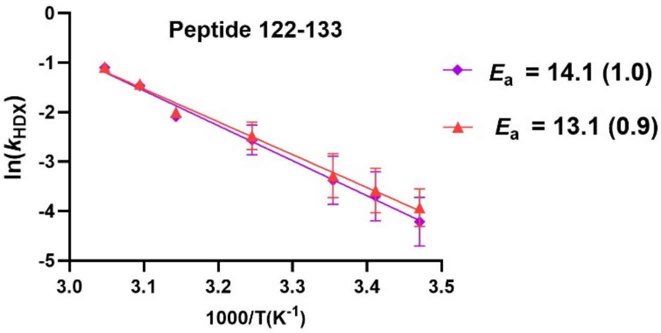
Peptide 122-133
E_a = 14.1 (1.0)
E_a = 13.1 (0.9)
ln(k_{HDX})
1000/T(K^{-1})

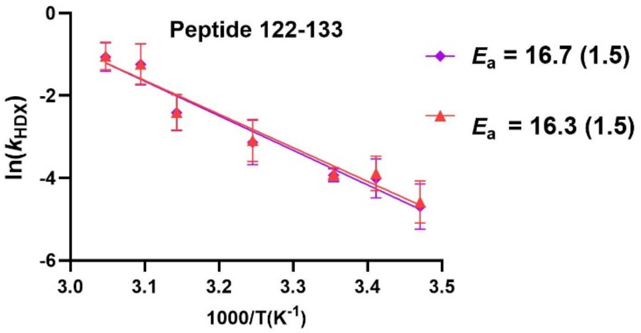
Peptide 122-133
E_a = 16.7 (1.5)
E_a = 16.3 (1.5)
ln(k_{HDX})
1000/T(K^{-1})

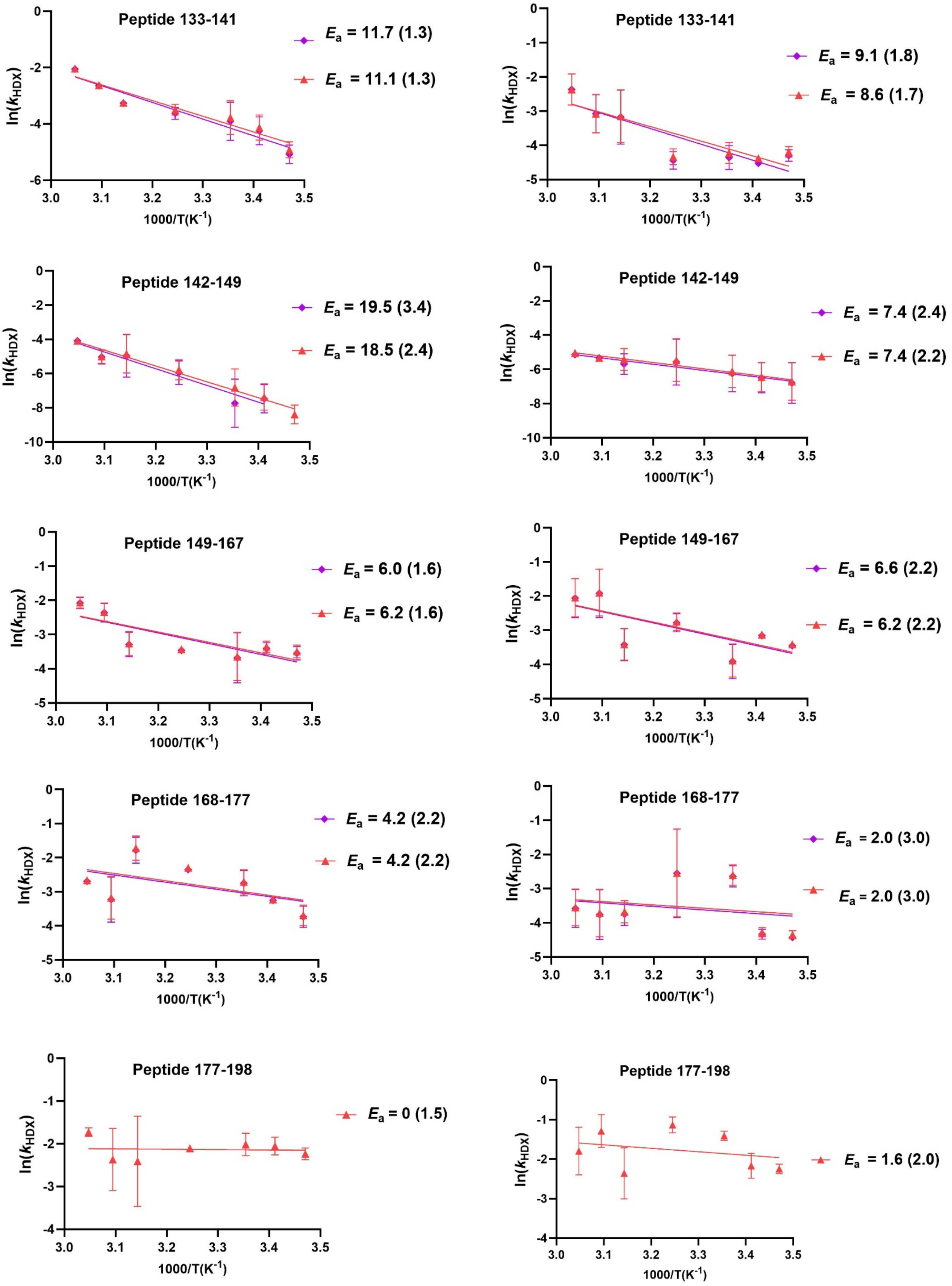

Peptide 133-141
Ea = 11.7 (1.3)
Ea = 11.1 (1.3)
Peptide 133-141
Ea = 9.1 (1.8)
Ea = 8.6 (1.7)
Peptide 142-149
Ea = 19.5 (3.4)
Ea = 18.5 (2.4)
Peptide 142-149
Ea = 7.4 (2.4)
Ea = 7.4 (2.2)
Peptide 149-167
Ea = 6.0 (1.6)
Ea = 6.2 (1.6)
Peptide 149-167
Ea = 6.6 (2.2)
Ea = 6.2 (2.2)
Peptide 168-177
Ea = 4.2 (2.2)
Ea = 4.2 (2.2)
Peptide 168-177
Ea = 2.0 (3.0)
Ea = 2.0 (3.0)
Peptide 177-198
Ea = 0 (1.5)
Peptide 177-198
Ea = 1.6 (2.0)
ln(kHDX)
1000/T(K-1)

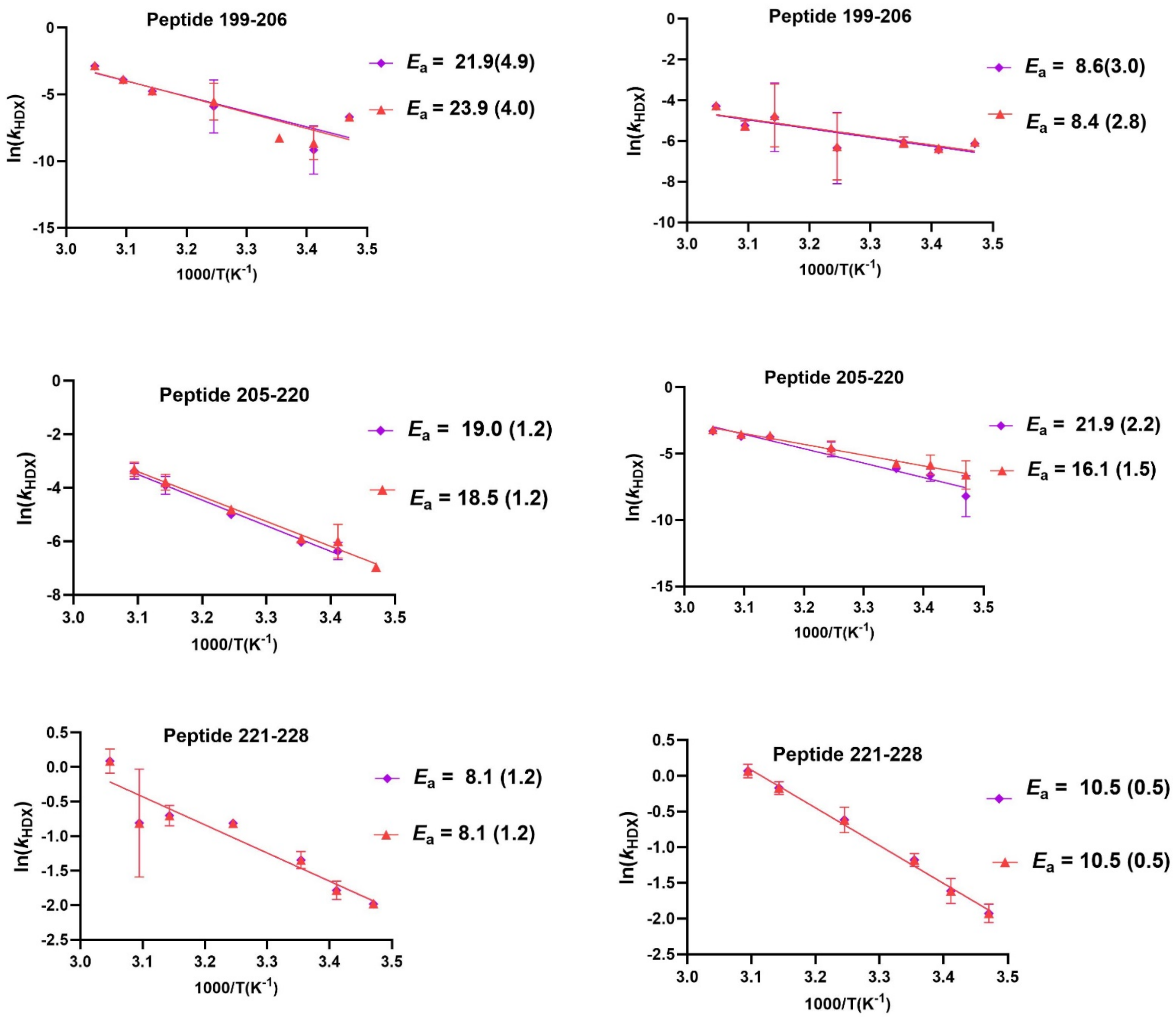

Peptide 199-206
E_a = 21.9(4.9)
E_a = 23.9 (4.0)
ln(k_{HDX})
1000/T(K^{-1})
Peptide 199-206
E_a = 8.6(3.0)
E_a = 8.4 (2.8)
ln(k_{HDX})
1000/T(K^{-1})
Peptide 205-220
E_a = 19.0 (1.2)
E_a = 18.5 (1.2)
ln(k_{HDX})
1000/T(K^{-1})
Peptide 205-220
E_a = 21.9 (2.2)
E_a = 16.1 (1.5)
ln(k_{HDX})
1000/T(K^{-1})
Peptide 221-228
E_a = 8.1 (1.2)
E_a = 8.1 (1.2)
ln(k_{HDX})
1000/T(K^{-1})
Peptide 221-228
E_a = 10.5 (0.5)
E_a = 10.5 (0.5)
ln(k_{HDX})
1000/T(K^{-1})

Table S14. Activation energy for HDX ($E_a(k_{HDX})$) for peptides in WT(L) and L123A(L) forms of Mt-OMPDC. Change in activation energy was estimate by subtracting $E_a(k_{HDX})$ of L123A(L) by WT(L). Values for each peptide were averaged from two biological replicate experiments. Peptides showing $\Delta E_a(k_{HDX})$ value larger than 2σ, where σ is standard propagated error, are highlighted in bold.

Peptide number	$E_a(k_{HDX})$; WT (L)	$E_a(k_{HDX})$; L123A (L)	$\Delta E_a(k_{HDX})$; (L123A(L) – WT (L))
**10-17**	**12.9 (0.6)**	**15.3 (0.6)**	**2.4 (0.6)**
28-36	6.6 (2.2)	7.8 (2.2)	1.2 (2.2)
**37-49**	**9.0 (0.9)**	**15.9 (1.2)**	**6.9 (1.1)**
**55-59**	**19.9 (1.5)**	**23.9 (1.0)**	**4.0 (1.3)**
60-71	6.8 (2.0)	9.7(2.0)	3.0 (2.0)
71-88	10.1 (2.4)	6.8 (5.0)	-3.4 (3.9)
97-110	12.3 (1.3)	13.5 (1.2)	1.2 (1.3)
**111-121**	**11.7 (0.6)**	**8.3 (0.8)**	**-3.4 (0.7)**
**122-133**	**13.1 (0.9)**	**16.3 (1.5)**	**3.2 (1.3)**
133-141	11.1 (1.3)	8.5 (1.7)	-2.6 (1.5)
**142-149**	**18.5 (2.2)**	**7.4 (2.2)**	**-11.1 (2.2)**
149-167	6.0 (1.6)	6.6 (2.2)	0.6 (1.9)
168-177	4.2 (0.6)	2.0 (3.0)	-2.2 (2.6)
**199-206**	**23.8 (4.0)**	**8.3 (2.8)**	**-15.5 (3.5)**
205-220	18.5 (1.2)	16.1 (1.5)	-2.4 (1.4)
**221-228**	**8.2 (1.2)**	**10.5 (0.5)**	**2.4 (0.9)**

Figure S11. Temperature and time dependent HDX traces of WT(apo) in right, and L123A(apo) in left. For WT(apo) data was collected between 15-55°C, whereas for L123(apo) it was between 15-50°C, as protein precipitated at 55°C. HDX data for all 19 peptides were from two independent biological replicates.

Temperature dependent HDX of apo forms were used at T_{melt}-HDX studies, to identify spatially resolved regions of Mt-OMPDC influencing thermal stability. Peptides with an abrupt increase in D-uptake in L123A(apo), a thermally unstable mutant compared to WT(apo), were highlighted with a black-colored boundary.

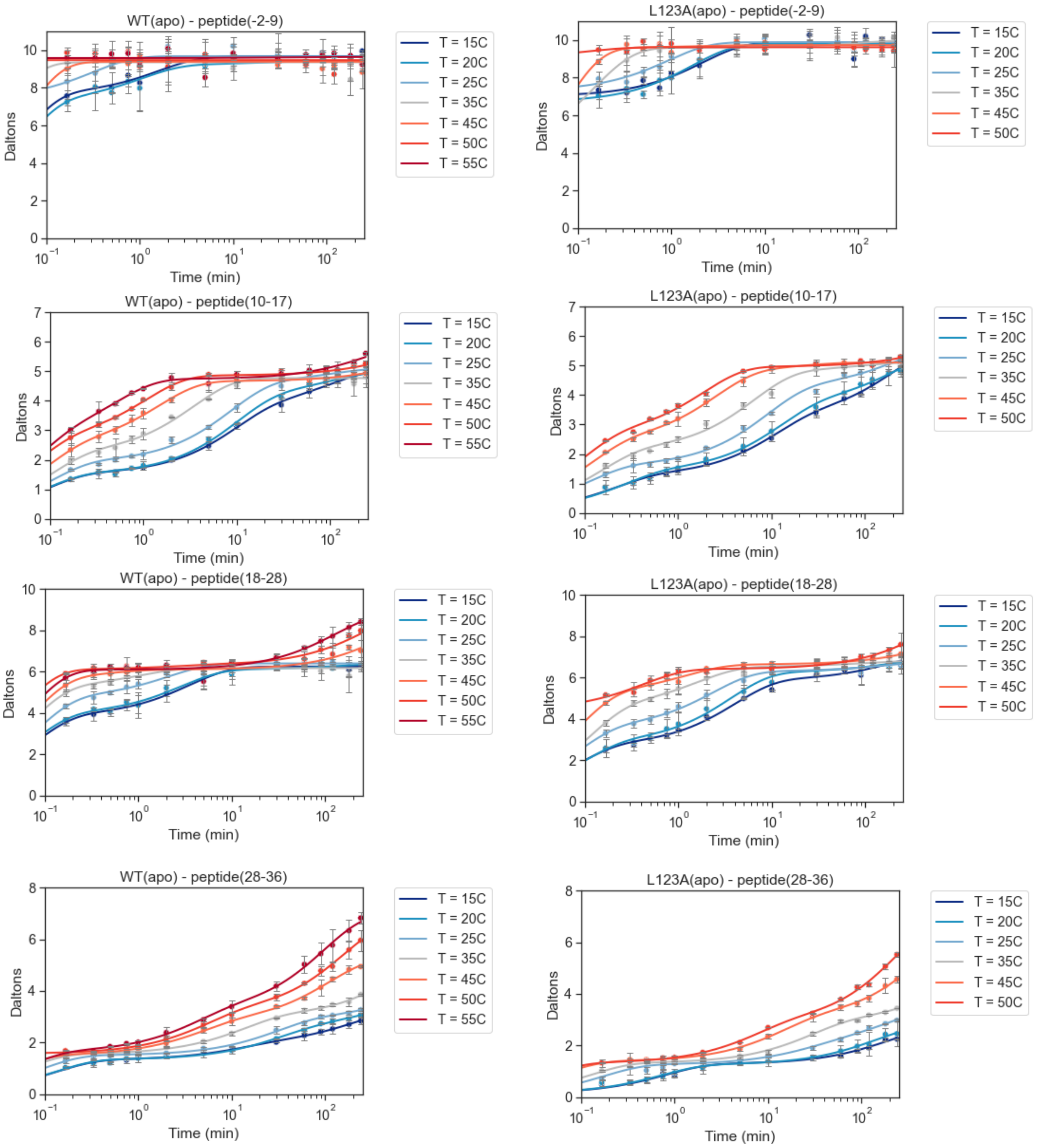

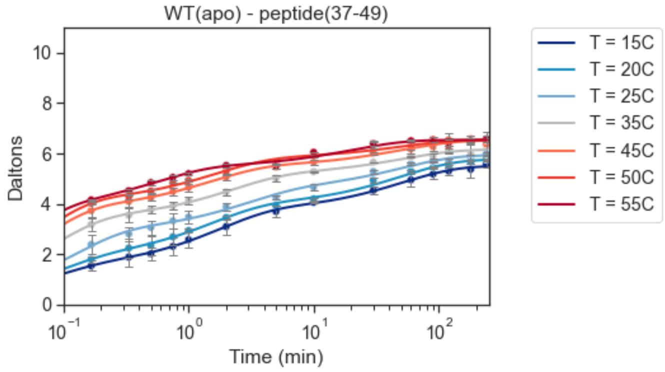
WT(apo) - peptide(37-49)
Daltons
Time (min)
T = 15C
T = 20C
T = 25C
T = 35C
T = 45C
T = 50C
T = 55C

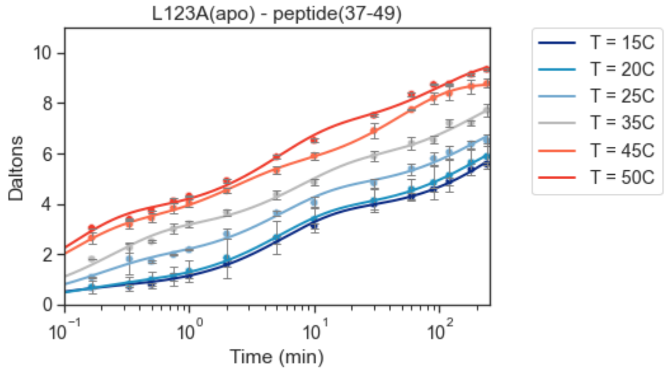
L123A(apo) - peptide(37-49)
Daltons
Time (min)
T = 15C
T = 20C
T = 25C
T = 35C
T = 45C
T = 50C

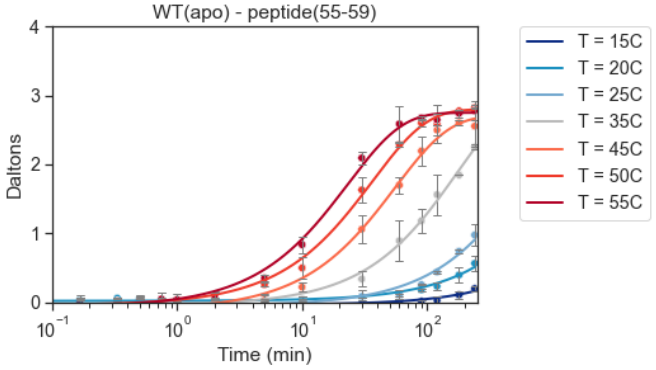
WT(apo) - peptide(55-59)
Daltons
Time (min)
T = 15C
T = 20C
T = 25C
T = 35C
T = 45C
T = 50C
T = 55C

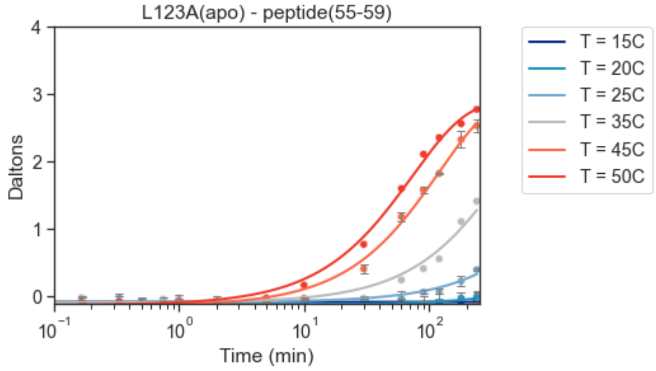
L123A(apo) - peptide(55-59)
Daltons
Time (min)
T = 15C
T = 20C
T = 25C
T = 35C
T = 45C
T = 50C

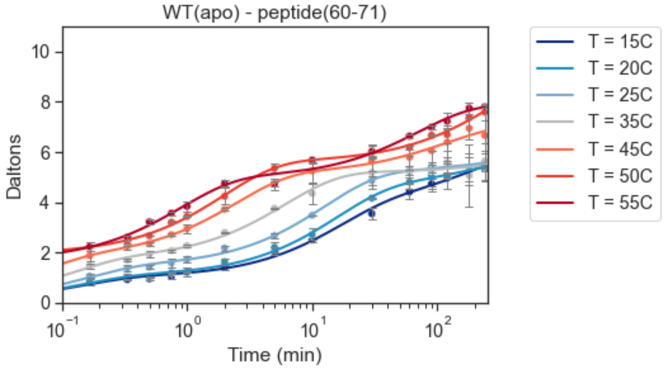
WT(apo) - peptide(60-71)
Daltons
Time (min)
T = 15C
T = 20C
T = 25C
T = 35C
T = 45C
T = 50C
T = 55C

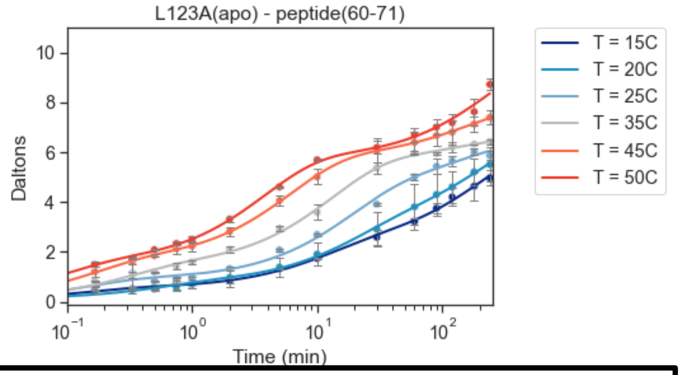
L123A(apo) - peptide(60-71)
Daltons
Time (min)
T = 15C
T = 20C
T = 25C
T = 35C
T = 45C
T = 50C

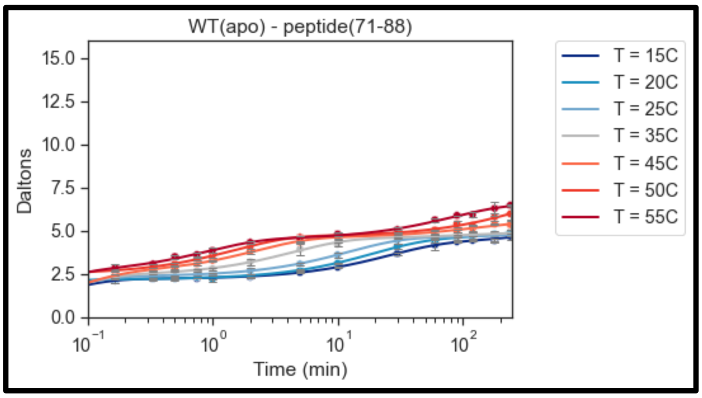
WT(apo) - peptide(71-88)
Daltons
Time (min)
T = 15C
T = 20C
T = 25C
T = 35C
T = 45C
T = 50C
T = 55C

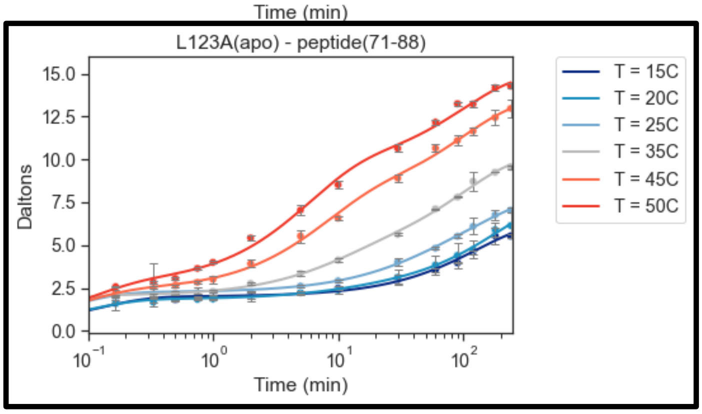
L123A(apo) - peptide(71-88)
Daltons
Time (min)
T = 15C
T = 20C
T = 25C
T = 35C
T = 45C
T = 50C

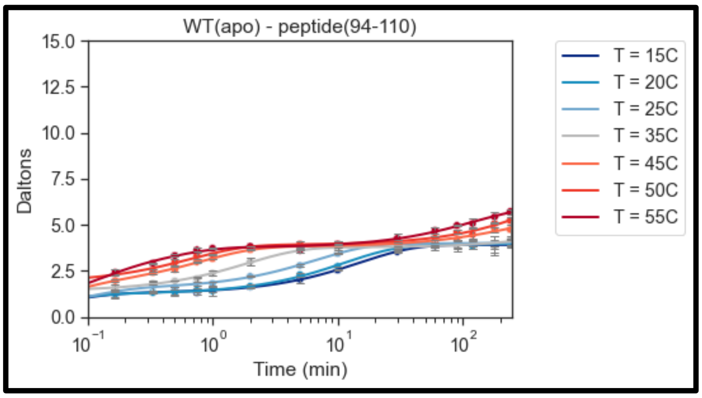
WT(apo) - peptide(94-110)
Daltons
Time (min)
T = 15C
T = 20C
T = 25C
T = 35C
T = 45C
T = 50C
T = 55C

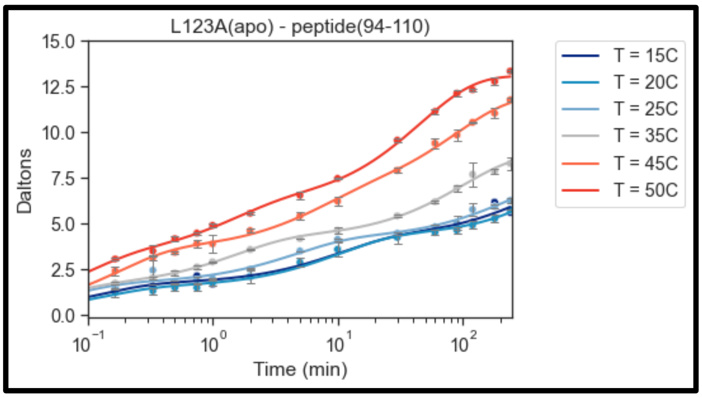
L123A(apo) - peptide(94-110)
Daltons
Time (min)
T = 15C
T = 20C
T = 25C
T = 35C
T = 45C
T = 50C

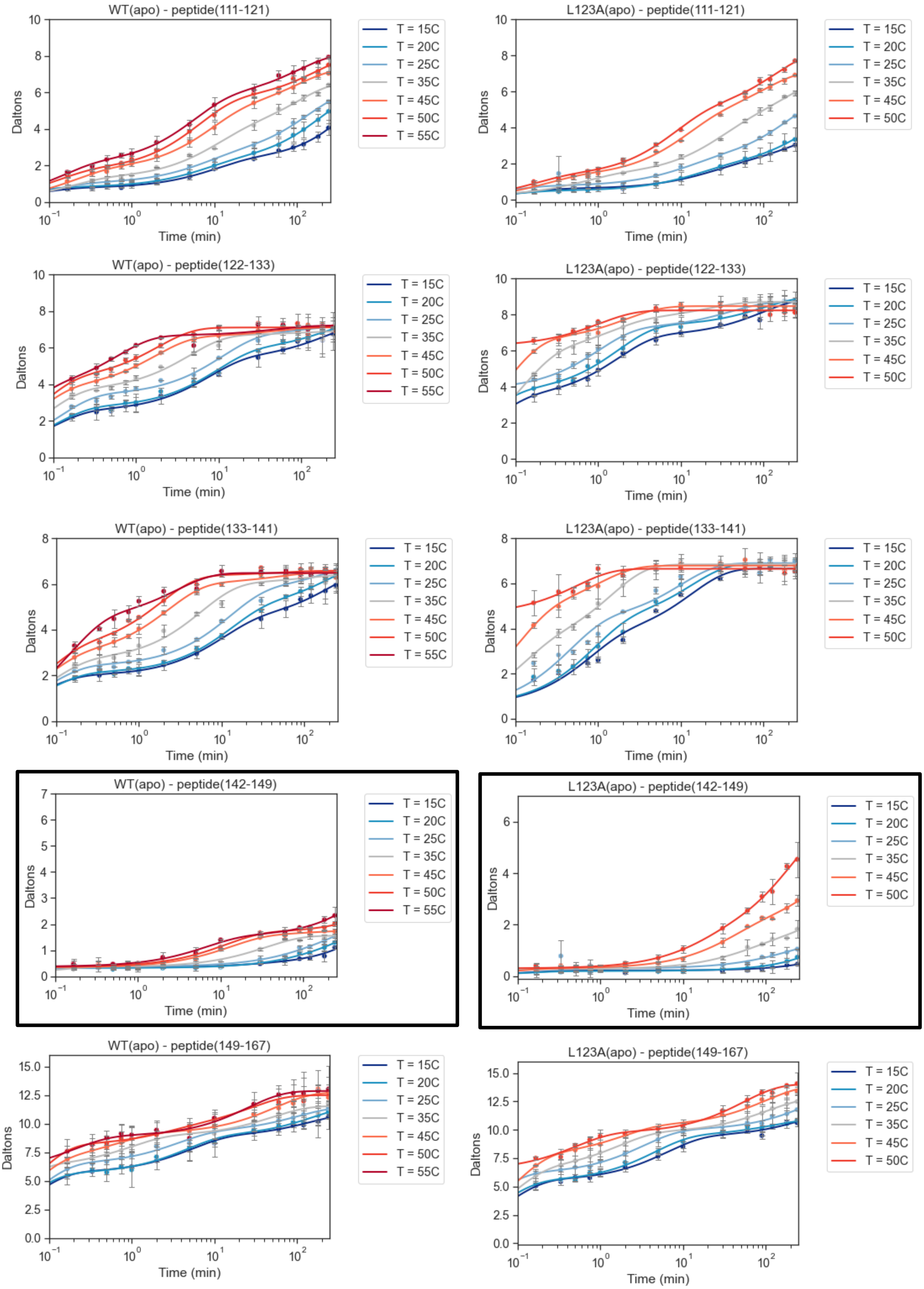
WT(apo) - peptide(111-121)
L123A(apo) - peptide(111-121)
WT(apo) - peptide(122-133)
L123A(apo) - peptide(122-133)
WT(apo) - peptide(133-141)
L123A(apo) - peptide(133-141)
WT(apo) - peptide(142-149)
L123A(apo) - peptide(142-149)
WT(apo) - peptide(149-167)
L123A(apo) - peptide(149-167)
Daltons
Time (min)
T = 15C
T = 20C
T = 25C
T = 35C
T = 45C
T = 50C
T = 55C

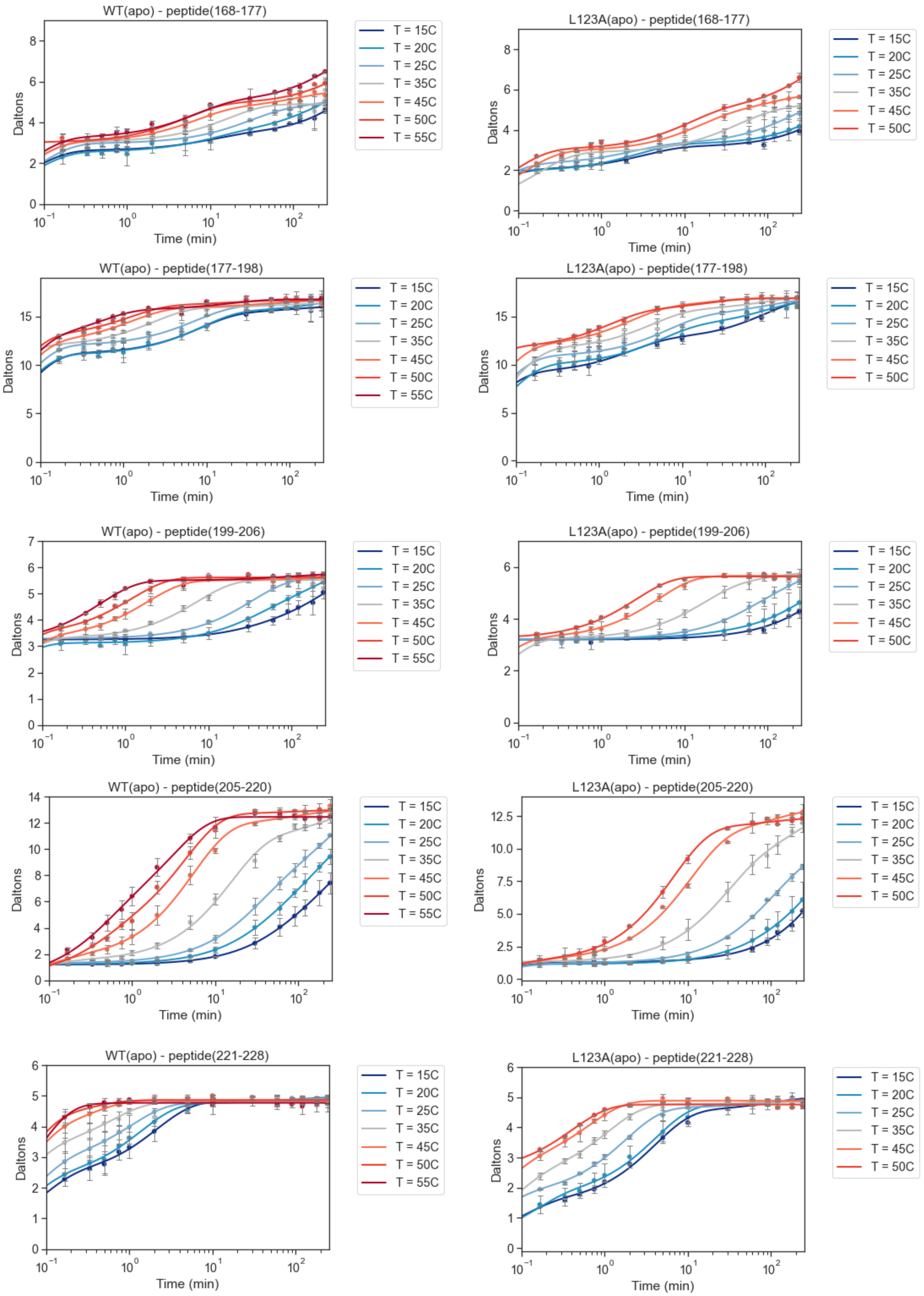
WT(apo) - peptide(168-177)
Daltons
Time (min)
T = 15C
T = 20C
T = 25C
T = 35C
T = 45C
T = 50C
T = 55C
L123A(apo) - peptide(168-177)
Daltons
Time (min)
T = 15C
T = 20C
T = 25C
T = 35C
T = 45C
T = 50C
WT(apo) - peptide(177-198)
Daltons
Time (min)
T = 15C
T = 20C
T = 25C
T = 35C
T = 45C
T = 50C
T = 55C
L123A(apo) - peptide(177-198)
Daltons
Time (min)
T = 15C
T = 20C
T = 25C
T = 35C
T = 45C
T = 50C
WT(apo) - peptide(199-206)
Daltons
Time (min)
T = 15C
T = 20C
T = 25C
T = 35C
T = 45C
T = 50C
T = 55C
L123A(apo) - peptide(199-206)
Daltons
Time (min)
T = 15C
T = 20C
T = 25C
T = 35C
T = 45C
T = 50C
WT(apo) - peptide(205-220)
Daltons
Time (min)
T = 15C
T = 20C
T = 25C
T = 35C
T = 45C
T = 50C
T = 55C
L123A(apo) - peptide(205-220)
Daltons
Time (min)
T = 15C
T = 20C
T = 25C
T = 35C
T = 45C
T = 50C
WT(apo) - peptide(221-228)
Daltons
Time (min)
T = 15C
T = 20C
T = 25C
T = 35C
T = 45C
T = 50C
T = 55C
L123A(apo) - peptide(221-228)
Daltons
Time (min)
T = 15C
T = 20C
T = 25C
T = 35C
T = 45C
T = 50C

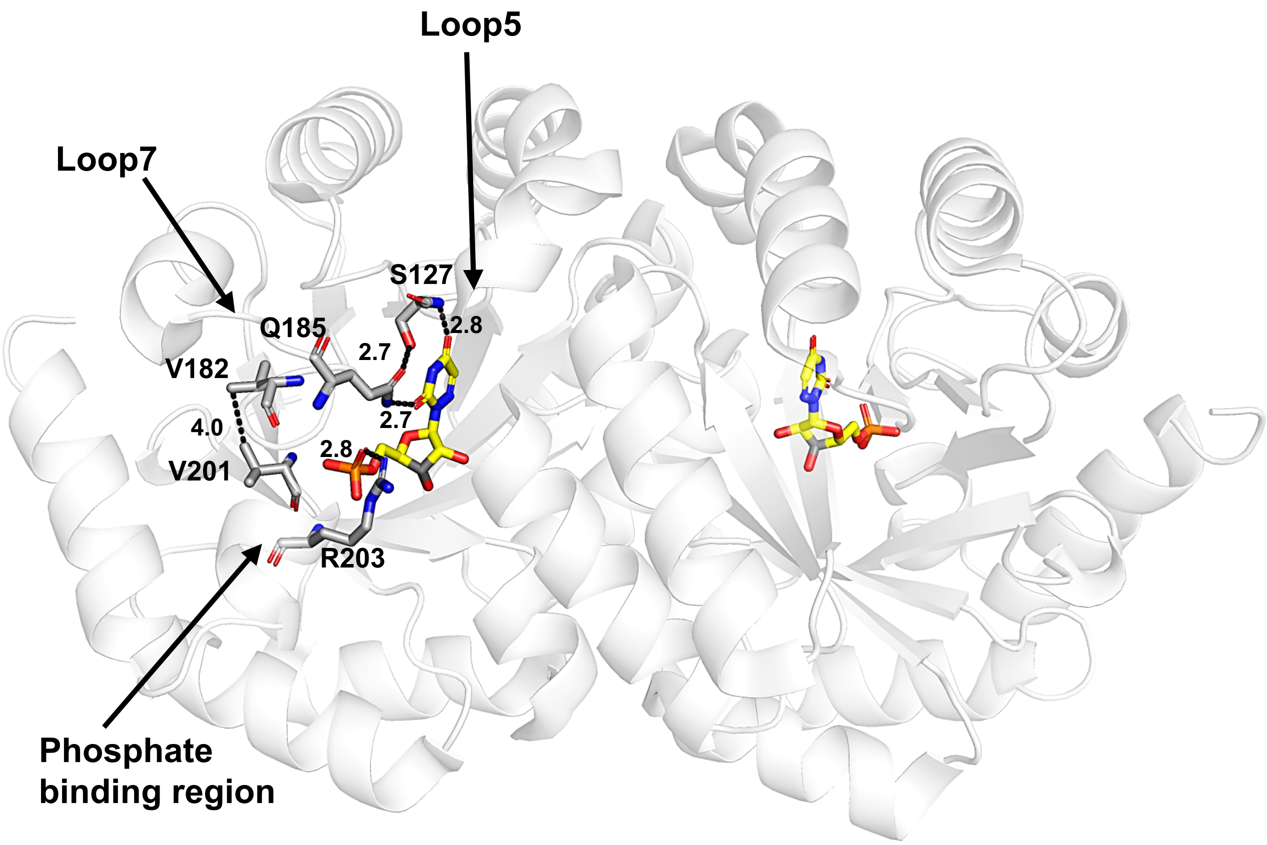

Figure S12. The crystal structure of Mt-OMPDC in the TSA-bound form reveals communication between loop5, loop7, and phosphate-binding region (loop8). Upon substrate binding, loop7 adopts a structured conformation, forming non-covalent interactions with loop5 (via Q185-S127) and loop8 (via V182-V201). Key residues involved are shown as sticks, highlighting hydrogen bonds and van der Waals contacts.

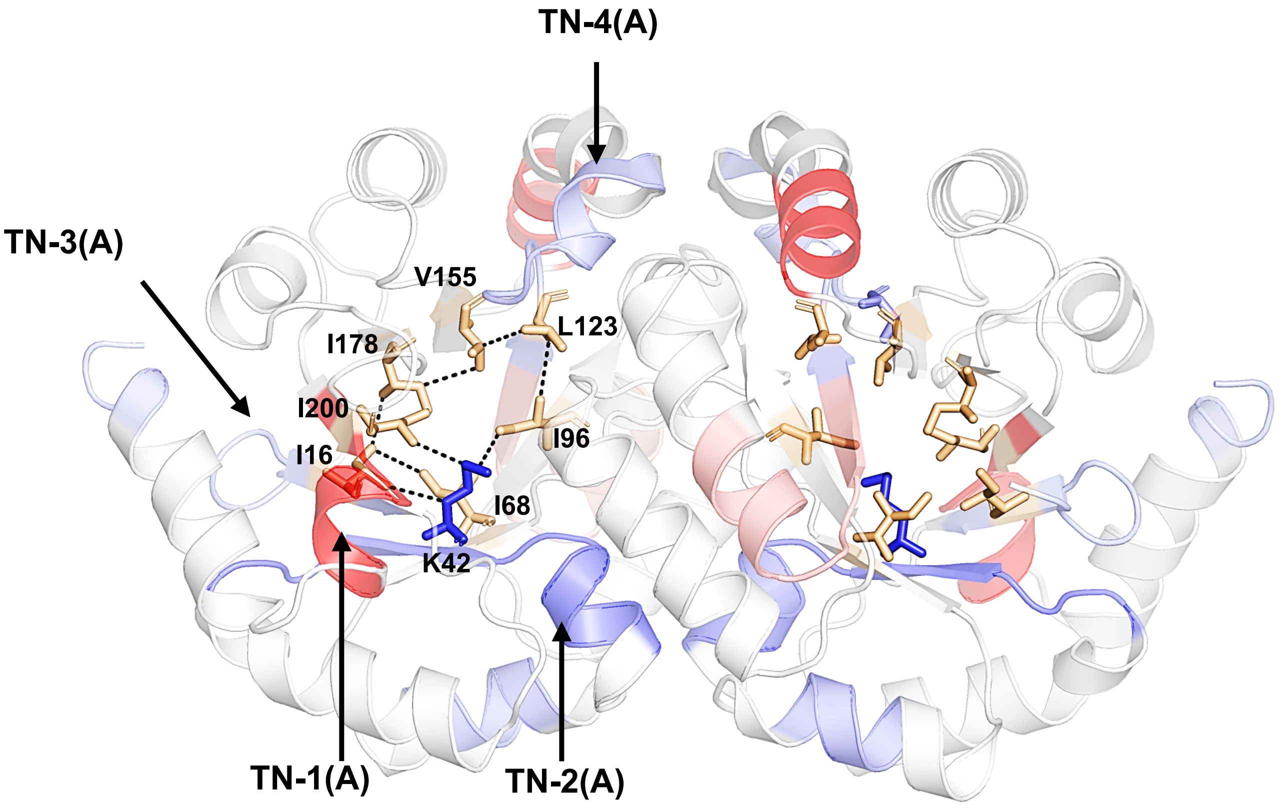

Figure S13. The ILV network, depicted in stick representation (gold), connects all four identified thermal networks, enabling their synergistic action. Van der Waals contacts between neighboring pairs are indicated by dotted lines. This network extends inward from the β-barrel, originating from seven β-sheets (excluding β2, which contains the catalytic residue K42). Notably, I200 is part of TN-1, I16 is part of TN-3, and L123 is part of TN-4. K42, a component of TN-2, establishes contacts with both I16 and I200

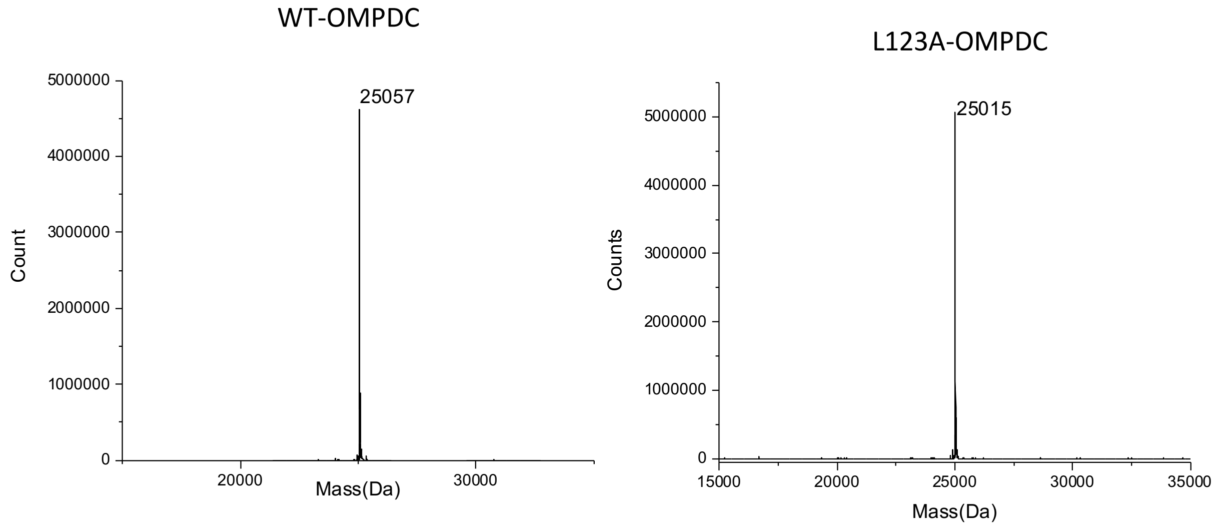

Figure S14. Intact mass spectra of wild-type and L123A mutants of Mt-OMPDC

Table S15. List of Primers used

Mutation	Forward	Reverse
L123A	GTCTTCCTCGCGACAGAGATG	CTCACGTCCCATTTCCTCTGC
L123I	GTCTTCCTCATTACAGAGATGTCAC	CTCACGTCCCATTTCCTCTGC
L123V	GTCTTCCTCGTGACAGAGATG	CTCACGTCCCATTTCCTCTGC
F71I	ATAGCCGACATCAAGGTTGCAG	GATTCTGCAGCCAAATCTCTTTC
F71V	ATAGCCGACGTTAAGGTTGCAG	GATTCTGCAGCCAAATCTCTTTC
F71G	ATAGCCGACGGTAAGGTTGCAG	GATTCTGCAGCCAAATCTCTTTCTG
L123G	GTCTTCCTCGTGACAGAGATGTCAC	CTCACGTCCCATTTCCTCTGC
M126A	CTGACAGAGGCGTCACACCC	GAGGAAGACCTCACGTCCC
M126L	CTGACAGAGCTGTCACACCC	GAGGAAGACCTCACGTCCC
M126V	CTGACAGAGGTGTCACACCC	GAGGAAGACCTCACGTCCC
M126I	CTGACAGAGATCTCACACCCAG	GAGGAAGACCTCACGTCCC
I83V	AATGAAAAGGTTTGCCGGGCC	GGTCTCGGGTATATCTGCAACC
V167A	GATGGTGTGGCGTGTTCTG	AAGGCCACATTTTTGCGTC
V167I	GATGGTGTGATCTGTTCTGC	AAGGCCACATTTTTGCGTC
V167L	GATGGTGTGCTGTGTTCTGC	AAGGCCACATTTTTGCGTC
V167G	GATGGTGTGGGCTGTTCTG	AAGGCCACATTTTTGCGTC
V187A	CAAACTGGCTACGCCGG	AACTCCTGACCGAATACCTG
V187I	CAAACTGATTACGCCGGG	AACTCCTGACCGAATACCTG
V187L	CAAACTGCTTACGCCGGG	AACTCCTGACCGAATACCTG
V187G	CAAACTGGGTACGCCGG	AACTCCTGACCGAATACCTG
I68L	CTGCAGAATCCTGGCCGACTTCAAG	CCAAATCTCTTTCTGAACTC
I68A	CTGCAGAATCGCGGCCGACTTCAAG	CCAAATCTCTTTCTGAACTC
I68G	CTGCAGAATCGGCGCCGACTTCAAG	CCAAATCTCTTTCTGAACTC
I68V	CTGCAGAATCGTGGCCGACTTCA	CCAAATCTCTTTCTGAACTCAG
I178L	TTCATTTCTCCTGTCCCCCGGTG	TCCTGACCTATGATTTCC
I178V	TTCATTTCTCGTTTCCCCCGGTG	TCCTGACCTATGATTTCC
I178A	TTCATTTCTCGCGTCCCCCGGTGTG	TCCTGACCTATGATTTCC
I178G	TTCATTTCTCGGCTCCCCCGGTGTG	TCCTGACCTATGATTTCC
I178A	TTCATTTCTCGCATCCCCCGGTG	TCCTGACCTATGATTTCCCTCAGC
I178L	TTCATTTCTCCTGTCCCCCGGTG	TCCTGACCTATGATTTCCCTCAGC
I178V	TTCATTTCTCGTGTCCCCCGGTG	TCCTGACCTATGATTTCCCTCAGC
I178G	TTCATTTCTCGGTTCCCCCGGTG	TCCTGACCTATGATTTCCCTCAGC

V155A	CAAAAATTATGCGGGCCCATCCAC	ACACCAAGATCGACCCCC
V155I	CAAAAATTATATCGGCCCATCCACAAGACC	ACACCAAGATCGACCCCC
V155L	CAAAAATTATCTGGGCCCATCCACAAGAC	ACACCAAGATCGACCCCC
V155G	CAAAAATTATGGTGGCCCATCCAC	ACACCAAGATCGACCCCC
I96A	GATGCCATAGCAGTCCACGGATTC	AGCCCCCGCCTTGAAG
I96L	TGATGCCATACTGGTCCACGGATTC	GCCCCCGCCTTGAAG
I96V	TGATGCCATAGTTGTCCACGGATTC	GCCCCCGCCTTGAAG

References

1. Toth, Krisztina; Amyes, Tina L.; Wood, B. Mc Kay; Chan, Kui K.; Gerlt, John A.; Richard, J. P. An examination of the relationship between active site loop size and thermodynamic activation parameters for orotidine 5′-monophosphate decarboxylase from mesophilic and thermophilic organisms. *Biochemistry* **48**, 8006–8013 (2009).
2. Wood, B. M. K. *et al.* Conformational changes in orotidine 5′-monophosphate decarboxylase: ‘remote’ residues that stabilize the active conformation. *Biochemistry* **49**, 3514–3516 (2010).
3. Offenbacher, Adam R.; Hu, Shenshen; Poss, Erin M.; Carr, Cody A. M.; Scouras, Alexander D.; Prigozhin, Daniil M.; Iavarone, Anthony T.; Palla, Ali; Alber, Tom; Fraser, James S.; Klinman, J. P. Hydrogen–Deuterium Exchange of Lipoxygenase Uncovers a Relationship between Distal, Solvent Exposed Protein Motions and the Thermal Activation Barrier for Catalytic Proton-Coupled Electron Tunneling. *ACS Cent. Sci.* **3**, 570–579 (2017).
4. Pascal, B. D. *et al.* HDX workbench: software for the analysis of H/D exchange MS data. *J. Am. Soc. Mass Spectrom.* **23**, 1512–1521 (2012).
5. Gao, Shuaihua; Thompson, Emily J.; Barrow, Samuel L.; Zhang, Wenju; Iavarone, Anthony T.; Klinman, J. P. Hydrogen–Deuterium Exchange within Adenosine Deaminase, a TIM Barrel Hydrolase, Identifies Networks for Thermal Activation of Catalysis. *J. Am. Chem. Soc.* **142**, 19936–19949 (2020).
6. Thompson, E. J., Paul, A., Iavarone, A. T. & Klinman, J. P. Identification of Thermal Conduits That Link the Protein–Water Interface to the Active Site Loop and Catalytic Base in Enolase. *J. Am. Chem. Soc.* **143**, 785–797 (2021).
7. Zhang, J., Balsbaugh, J. L., Gao, S., Ahn, N. G. & Klinman, J. P. Hydrogen deuterium exchange defines catalytically linked regions of protein flexibility in the catechol O-methyltransferase reaction. *Proc. Natl. Acad. Sci.* **117**, 10797–10805 (2020).
8. Shandilya, S., Vertrees, J. & Holder, T. ColorByRMSD. (2012).
